# Generation of Site‐Specifically Labeled Affinity Reagents via Use of a Self‐Labeling Single Domain Antibody

**DOI:** 10.1002/advs.202417160

**Published:** 2025-02-18

**Authors:** Stanley Fayn, Swarnali Roy, Chino C. Cabalteja, Woonghee Lee, Hima Makala, Kwamena Baidoo, Divya Nambiar, Julia Sheehan‐Klenk, Joon‐Yong Chung, Jesse Buffington, Mitchell Ho, Freddy E. Escorcia, Ross W. Cheloha

**Affiliations:** ^1^ Molecular Imaging Branch Center for Cancer Research National Cancer Institute National Institutes of Health Bethesda MD 20892 USA; ^2^ Oxford Institute for Radiation Oncology Department of Oncology University of Oxford Oxford OX3 7DQ UK; ^3^ Chemical Biology in Signaling Section National Institutes of Diabetes and Digestive and Kidney Diseases National Institutes of Health Bethesda MD 20892 USA; ^4^ Antibody Engineering Program Center for Cancer Research National Cancer Institute National Institutes of Health Bethesda MD 20892 USA; ^5^ Laboratory of Molecular Biology Center for Cancer Research National Cancer Institute National Institutes of Health Bethesda MD 20892 USA; ^6^ Radiation Oncology Branch Center for Cancer Research National Cancer Institute National Institutes of Health Bethesda MD 20892 USA

**Keywords:** antibodies, conjugation, molecular imaging, nanobody, peptide

## Abstract

Several chemical and enzymatic methods have been used to link antibodies to moieties that facilitate visualization of cognate targets. Emerging evidence suggests that the extent of labeling, dictated by the type of chemistry used, has a substantial impact on performance, especially in the context of antibodies used for the visualization of tumors in vivo. These effects are particularly pronounced in studies using small antibody fragments, such as single‐domain antibodies, or nanobodies. Here, we leverage a new variety of conjugation chemistry, based on a nanobody that forms a crosslink with a specialized high‐affinity epitope analogue, to label target‐specific nanobody constructs with functionalities of choice, including fluorophores, chelators, and click chemistry handles. Using heterodimeric nanobody conjugates, comprised of an antigen recognition module and a self‐labeling module, enables us to attach the desired functional group at a location distal to the site of antigen recognition. Constructs generated using this approach bound to antigens expressed on xenograft murine models of liver cancer and allowed for non‐invasive diagnostic imaging. The modularity of our approach using a self‐labeling nanobody offers a novel method for site‐specific functionalization of biomolecules and can be extended to other applications for which covalent labeling is required.

## Graphical abstract

Several methods have been used to label antibodies to facilitate visualization of cognate targets. Here, we leverage a new variety of conjugation chemistry, based on a crosslinking nanobody, to label target‐specific nanobody constructs with functionalities of choice. Constructs generated using this approach bound to antigens expressed on xenograft murine models of liver cancer and allowed for non‐invasive diagnostic imaging.

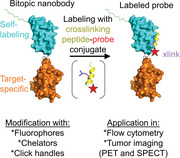

## 1 Introduction

Methods to analyze intact cells and proteins expressed within those cells are important for understanding biological function. Monoclonal antibodies (Abs) are deployed widely throughout biomedicine for the specific recognition and detection of targets of interest. Due to their high antigen specificity and affinity, Abs are often considered the gold standard for the characterization of biomolecules of interest. High fidelity and affinity recognition can also be achieved using antigen‐binding fragments of antibodies, with the smallest of these fragments deriving from the variable domain of camelid heavy chain only antibodies (V_H_Hs or nanobodies, Nbs).^[^ ^1^ ^]^ Abs and Nbs are routinely used for cellular biochemical analysis, interrogation of spatial organization using microscopy, and enrichment techniques for more detailed analysis.^[^ ^1^ ^]^

The sensitive and robust detection of cells expressing antigens of interest has naturally enabled the application of Abs as reagents for visualizing and tracking cells within animals, including humans.^[^ ^2^, ^3^, ^4^ ^]^ Nbs are especially appealing as reagents for imaging because their rapid clearance from the bloodstream by kidney filtration offers the prospect for same‐day imaging, in contrast to conventional Abs, which circulate for prolonged periods and typically require several day intervals between administration and imaging to achieve useful signal‐to‐noise ratios in diagnostic imaging settings.^[^ ^5^ ^]^ Substantial effort has been extended in developing Ab‐based imaging reagents for the detection of tumor cells with the goal of providing information on tumor (or metastasis) localization and reporting on changes in cancer cell abundance and distribution over time in response to treatment.^[^ ^6^, ^7^ ^]^

Imaging applications typically require that Abs and Nbs be modified through attachment to moieties that permit visualization, such as fluorophores, radioisotopes, or other detectable groups. Abs typically contain several amino acids with side chains that can serve as sites for modification. Reagents that react with lysine or cysteine side chains are frequently used.^[^ ^8^ ^]^ Most lysine‐ or cysteine‐reactive labeling reagents are not specific for an individual residue, with some level of reactivity for many residues with an appropriately exposed side chain. Abs are large proteins and contain many lysine residues. Labeling with non‐specific lysine‐reactive reagents can result in an extensively and heterogeneously labeled product with a loss of performance in imaging applications, particularly in vivo. This effect is especially marked for small Ab fragments such as Nbs, which are comprised of a single domain and are susceptible to non‐specific modifications near the antigen binding site.^[^ ^9^, ^10^ ^]^ Alternatively, most cysteine residues in Abs are engaged in intra‐ and inter‐molecular disulfide bonds and are therefore unavailable for labeling applications. Although many groups have successfully deployed non‐specific labeling technologies to produce imaging reagents, growing evidence suggests that alternative chemistries that provide site‐specific labeling can offer imaging reagents with improved properties.^[^ ^9^, ^10^, ^11^ ^]^

Site‐specific labeling of Abs can be achieved through a variety of approaches. The most common involves the addition of a cysteine residue not involved in a structural disulfide bond, which can enable labeling using thiol‐reactive groups such as maleimide or haloacetamide moieties. A drawback of this approach is that such cysteine residues are prone to oxidation and incorporation into disulfide bonds, thereby requiring the incorporation of an additional reduction step prior to labeling. Other, more recently developed approaches rely on the site‐specific recognition and modification of sequence motifs engineered into antibodies by enzymes such as Sortase A, asparaginyl endopeptidases, and glycotransferases.^[^ ^12^, ^13^, ^14^, ^15^ ^]^ Enzymatic labeling provides straightforward modification chemistry, but commonly used protocols require prolonged conjugation reactions that use an excess (>5 equivalents) and high concentration (mM level) of labeling reagents, which can be prohibitive for exotic, expensive, or hydrophobic (insoluble) labeling reagents.

Herein, we describe a new method for labeling that relies on the genetic fusion of antigen‐specific Nb module with a self‐labeling Nb module.^[^ ^16^ ^]^ These heterodimeric constructs maintain high affinity and specificity for targeted antigens and can be labeled using a reagent that specifically modifies the self‐labeling Nb module. The reaction used for modification of the self‐labeling Nb module within the heterodimeric construct is operationally simple and rapid, requiring only mid‐micromolar levels of labeling reagent. We validated constructs amenable to labeling using this approach as useful biochemical reagents for detecting target binding to either purified protein or to antigens expressed on the cell surface. We further evaluated these heterodimeric constructs as reagents for the visualization of liver cancer in mice. Past work has shown that targeting the liver cancer‐selective marker glypican‐3 (GPC3) using a Nb‐based radiological imaging reagent (nanobody HN3) enables tumor imaging with positron emission tomography (PET) and the potential delivery of therapeutic cargo.^[^ ^10^, ^17^, ^18^ ^]^ Such tumor‐selective imaging is critical for diagnosis, assessing treatment response, and surveillance of patients with liver cancer. Application of a heterodimeric Nb construct, comprised of modules that bind GPC3 and facilitate self‐labeling, enabled the production of target‐selective imaging agents that visualized liver cancer tumors in mice. This approach was used to generate agents compatible with both single photon emission computed tomography (SPECT) and PET/CT modalities.

## 2 Results

**Figure 1:**
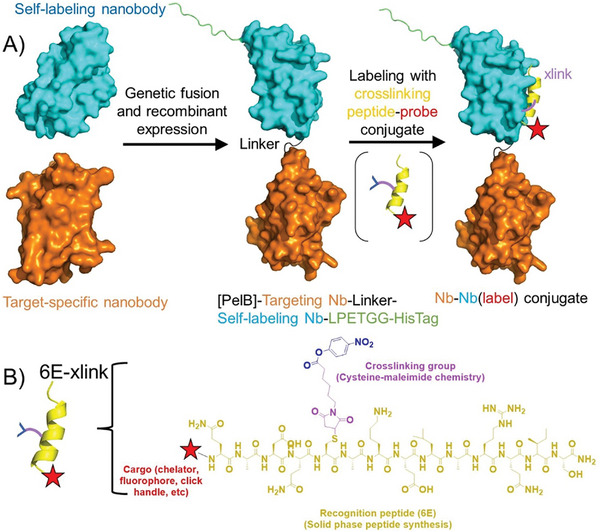
Approach for application of self‐labeling Nb dimer constructs. A) Previously described target‐specific Nbs^[^ ^19^, ^20^ ^]^ were genetically fused to an Nb that can engage in a crosslinking reaction with an epitope peptide^[^ ^16^ ^]^ modified to contain an electrophilic crosslinking group. Models were generated using Alphafold2. B) Purified dimeric Nb constructs were labeled using a crosslinking peptide probe (6E‐xlink) linked to a moiety of interest such as chelators, fluorophores, or click chemistry handles. These probes were synthesized as described in Supporting Methods (Supporting Information).

Bispecific Nb‐based constructs designed to target antigens of interest and enable chemical modification via the action of a self‐labeling Nb module were generated through recombinant expression. Previously described Nbs that target either the mouse immunoglobulin light chain‐kappa (Ig‐κ bound by Nb_Kappa_)^[^ ^19^ ^]^ or human GPC3 (bound by HN3)^[^ ^20^ ^]^ were genetically fused to a Nb raised against the protein UBC6e (Nb_6E_)^[^ ^21^, ^22^ ^]^ (**Figure** **1**a) that can bind to and undergo a crosslinking reaction with a 14‐mer peptide epitope equipped with an electrophilic phenolic ester crosslinking group (Figure 1b).^[^ ^16^ ^]^ Each construct contained an N‐terminal pelB leader sequence for periplasmic trafficking and folding, a short peptide linker between each Nb module, and a C‐terminal sortase A recognition motif (LPETG) followed by a hexahistidine tag.

**Figure 2:**
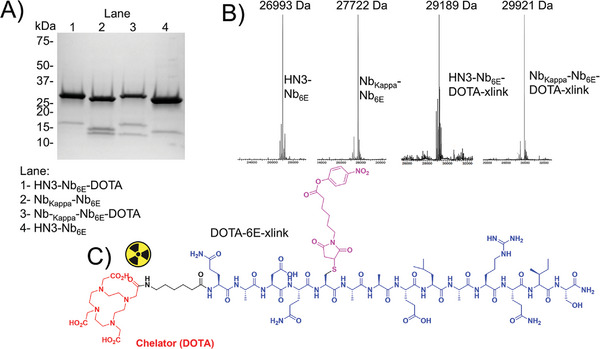
Demonstration of crosslinking for dimeric Nb constructs. Heterodimeric Nb constructs were produced via recombinant expression and purified as described in Experimental Section. A, B) The purity and identity of starting dimeric nanobody constructs and the products of crosslinking reactions were characterized via A) SDS‐PAGE and B) mass spectrometry as described in Experimental Section. Crosslinking was performed by exposure of purified Nb constructs (20 µm) in PBS with DOTA‐6E‐xlink (60 µm, structure in panel C). Crude reactions were analyzed by SDS‐PAGE and mass spectrometry. The uncropped gel for panel A and uncropped mass spectra for panel B are found in Supporting Information. Note that panel A, lane 4 is reproduced in Figure S8B (Supporting Information).

These heterodimeric constructs were produced in BL21 (DE3) *E. coli*. The desired proteins were purified from bacterial lysates by sequential Ni‐NTA metal ion affinity chromatography and size‐exclusion chromatography. Recovered proteins were characterized by mass spectrometry and sodium dodecyl sulfate‐polyacrylamide gel electrophoresis (SDS‐PAGE). SDS‐PAGE analysis revealed major bands that corresponded to expected construct sizes (≈30 kDa), along with weak bands at lower molecular weights (10–20 kDa) corresponding to impurities (**Figure** **2**). Mass spectrometry analyses were consistent with findings from SDS‐PAGE (Figure 2b, Supporting Information). Yields were adequate (0.5–5 mg L^−1^ of culture) for characterization in selected subsequent applications.

We sought to assess whether these purified proteins were competent to participate in the self‐labeling reaction previously described for the Nb_6E_ monomer.^[^ ^16^ ^]^ The synthetic method used to produce 6E‐xlink (Figure 1B) provides a straightforward route to access analogs with other functional groups appended to the N‐terminus of the crosslinking peptide (see Supporting Methods, Supporting Information for synthetic details). We prepared an analog of 6E‐xlink with DOTA (2,2′,2′′,2′′′‐(1,4,7,10‐Tetraazacyclododecane‐1,4,7,10‐tetrayl)tetraacetic acid) attached via an aminohexanoic (Ahx) linker at the N‐terminus (Supporting Methods, Supporting Information). Labeling of HN3‐Nb_6E_ and Nb_Kappa_‐Nb_6E_ with DOTA‐6E‐xlink was assessed with characterization by SDS‐PAGE and mass spectrometry (Figure 2a,b, Supporting Information). This labeling reaction proceeded to near completion upon exposure of HN3‐Nb_6E_ or Nb_Kappa_‐Nb_6E_ (20 µm) to 3 equivalents of (60 µm) DOTA‐6E‐xlink (Figure 2c) at 4 °C for 2.5 h. SDS‐PAGE analysis demonstrated major bands that correspond to the expected sizes. Impurities that ran in the range of 10–20 kDa changed migration upon treatment with DOTA‐6E‐xlink (compare low molecular weight bands in lanes 1 + 4 in Figure 2a), suggesting these impurities correspond to Nb_6E‐_containing fragments.

**Figure 3:**
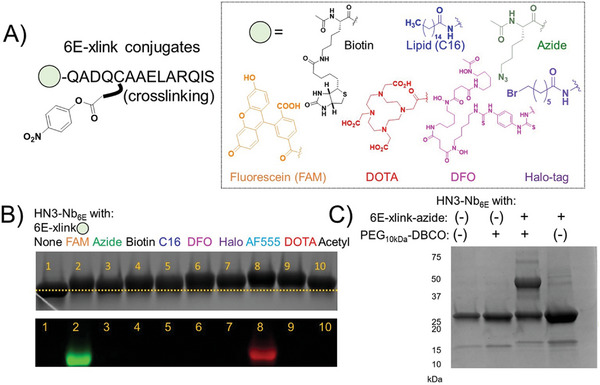
Evaluation of crosslinking chemistry for the appendage of varied cargos to HN3‐Nb_6E_. A) Analogues of 6E‐xlink were synthesized and characterized according to Experimental Section. B) Labeling was evaluated by a change in mobility on SDS‐PAGE (upper) or the appearance of fluorescent bands that result from covalent labeling with fluorophore‐labeled analogs of 6E‐xlink (lower). Crosslinking was performed by incubation of HN3‐Nb_6E_ (40 µm) with 6E‐xlink analog (60 µm) for 30 m at room temperature with quenching by addition of Laemmli sample buffer with DTT. C) An alternative assessment of crosslinking was performed using azide‐6E‐xlink with post‐crosslinking exposure of the product to PEG_10kDa_‐DBCO. HN3‐Nb_6E_ (40 µm) was incubated with azide‐6E‐xlink (60 µm) for 30 m at room temperature followed by the addition of PEG_10kDa_‐DBCO at a concentration of 250 µm for 1 h. Samples were treated with SDS containing sample buffer with DTT and analyzed by SDS‐PAGE. The uncropped gels for panels b and c are shown in Supporting Information.

To assess the generalizability of this approach, we synthesized several analogs of 6E‐xlink with moieties such as fluorophores, metal chelators, and click chemistry handles attached at the peptide N‐terminus (**Figure** **3**a; Figure S1, Supporting Information). Most conjugates were produced using standard solid phase‐peptide synthesis methodology, whereas others required more specialized synthetic approaches (Supporting Methods, Supporting Information). Many reactions resulted in the attachment of large moieties to HN3‐Nb_6E_ that are weakly ionized and poorly detected by mass spectrometry, complicating analysis using this method. We thus analyzed reactions by SDS‐PAGE. Relative to a negative control (unlabeled HN3‐Nb_6E_ in Figure 3b, Lane 1) all labeling reactions provided samples that migrated more slowly in SDS gel electrophoresis (Figure 3b, Lanes 2–10), consistent with an increase in molecular weight. Samples of HN3‐Nb_6E_ treated with analogs of 6E‐xlink containing fluorophores also showed in‐gel fluorescence of the expected sizes and color (Figure 3b, Supporting Information). A comparison of the kinetics of labeling HN3‐Nb_6E_ with fluorescein using a crosslinking peptide‐based chemistry to that observed via labeling with a Sortase A enzymatic approach (sortagging) showed that the crosslinking approach was substantially faster (Figure S2, Supporting Information). Labeling HN3‐Nb_6E_ with an azide‐containing analog of 6E‐xlink enabled subsequent reaction with PEG_10kDa_‐DBCO and the formation of a high molecular weight product (Figure 3c). Under these conditions, we confirmed that labeling of HN3‐Nb_6E_ was complete through analysis with mass spectrometry (see mass spectrum in Supporting Information), suggesting that the residual band just above 25 kDa was from an incomplete reaction of HN3‐Nb_6E_‐azide with PEG_10Kda_‐DBCO_._ The use of an analog of 6E‐xlink containing a chemical handle recognized by the self‐labeling Halo‐tag protein resulted in the formation of multiple high molecular weight complexes, which was dependent on the presence of HN3‐Nb_6E_ + Halo‐6E‐xlink and Halo tag protein (Figure S3, Supporting Information).^[^ ^23^ ^]^ The origin of the multiple crosslinked products observed in this reaction is unclear but is likely related to the bis‐electrophilic nature of the Halo‐6E‐xlink reagent. We confirmed that HN3‐Nb_6E_ is labeled with high specificity by FAM‐6E‐xlink even in the presence of a complex biological mixture (cell lysate), further supporting the specificity of 6E‐xlink reagents (Figure S4, Supporting Information). These findings confirm that Nb_6E_ maintains crosslinking capacity in the context of Nb heterodimer constructs. The facile preparation of crosslinking analogs with diverse moieties and their straightforward application to labeling thus offers a highly adaptable method to label heterodimeric Nb constructs.

**Figure 4:**
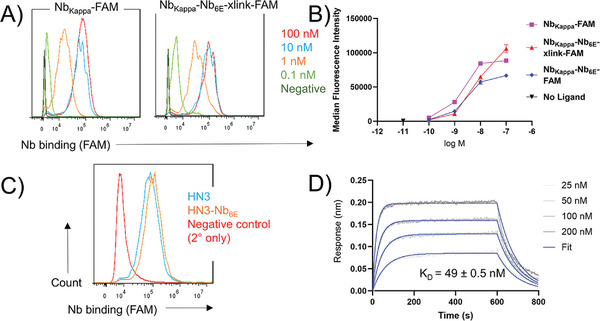
Assessment of target binding by labeled dimeric Nb constructs. A) Representative histograms of Nb_Kappa_‐Nb6_E_ (labeled using crosslinking) or Nb_Kappa_ (labeled using sortagging) to A20 cells. B) Representative, quantified dose‐response for the binding of labeled dimeric or monomeric Nb constructs to A20 cells. Data points correspond to mean ± SD from technical duplicates. Data from independent replicate experiments are shown in Figure S5 (Supporting Information). C) Histogram showing binding of dimeric or monomeric Nb constructs (1 µm) to A431 cells stably transfected to express GPC3 (G1 cell line). Binding was detected with an anti‐His tag secondary (2°) antibody as described in Supporting Methods (Supporting Information). D) Biolayer interferometry analysis of the binding of HN3‐Nb_6E_ labeled with DOTA using crosslinking chemistry. Gray lines correspond to experimental data and blue lines to the experimental models fit to this data. The binding of HN3‐Nb_6E_ analogs labeled with other moieties is shown in Figure S7 (Supporting Information). Biolayer interferometry binding experiments were performed as described in Experimental Section.

A different approach was used to assess whether the antigen binding module within heterodimeric Nb constructs maintained affinity for their targets. Nb_Kappa_ binds to Ig‐κ found within B‐cell receptors expressed on the surface of the murine B cells and the B‐cell lymphoma cell line A20.^[^ ^24^ ^]^ We compared the binding of Nb_Kappa_ monomer to that of Nb_Kappa_‐Nb_6E_. Nb_Kappa_ was produced as previously described^[^ ^24^ ^]^ and was site‐specifically labeled at its C‐terminus with fluorescein (Nb_Kappa_‐FAM) using Sortase A labeling (sortagging, **Figure** **4**a,b). Dimeric Nb_Kappa_‐Nb_6E_ was labeled with fluorescein either using sortagging (Nb_Kappa_‐Nb_6E_‐FAM) or with fluorescein‐6E‐xlink reagent (Nb_Kappa_‐Nb_6E_‐xlink‐FAM). These preparations were assessed by analysis of their binding to A20 cells using flow cytometry (Figure 4a,b; Figure S5, Supporting Information). The method of labeling had a negligible impact on the performance of the Nb constructs in staining A20 cells. The staining potency and maximal staining intensity were consistent between Nb_Kappa_‐FAM, Nb_Kappa_‐Nb_6E_‐FAM, and Nb_Kappa_‐Nb_6E_‐xlink‐FAM preparations (Figure 4a,b). This finding confirms that Nb_Kappa_ maintains affinity for Ig‐κ when produced in the context of Nb heterodimeric constructs and that labeling Nb_Kappa_‐Nb_6E_ either with sortagging or with crosslinking does not diminish binding.

We also tested the performance of HN3‐Nb_6E_ for binding to cognate target GPC3. We compared the binding of HN3‐Nb_6E_ to monomeric HN3 via assessment of staining a tumor cell line (A431) transfected to express GPC3 (G1) using flow cytometry (Figure 4c).^[^ ^20^ ^]^ HN3‐Nb_6E_ and HN3 stained the G1 cell line with similar intensity. In contrast, neither HN3‐Nb_6E_ nor HN3 stained A431 cells not expressing GPC3 (Figure S6, Supporting Information). We also assessed the binding of preparations of HN3‐Nb_6E_ labeled with 6E‐xlink analogs possessing azide, DOTA, Alexafluor 555, or palmitic acid (see Figure S1, Supporting Information for structures) to purified GPC3. Analysis with biolayer interferometry demonstrated that all analogs of HN3‐Nb_6E_ bound to immobilized GPC3 with K_d_ values in the range of 20–100 nm (Figure 4d; Figure S7, Supporting Information), similar to past measurements for other HN3 fusion proteins,^[^ ^25^ ^]^ thus validating that HN3‐based Nb heterodimeric constructs labeled with crosslinking reagents maintain binding.

Given the successful development of labeling chemistry for Nb heterodimeric constructs with retention of binding affinity and specificity, we applied these probes for in vivo applications. HN3‐Nb_6E_ for this application was produced at a large scale through recombinant expression in a mammalian expression system (see Experimental Section). Past work has shown that monomeric HN3 can be labeled to visualize implanted human liver cancer tumors in immunodeficient mice.^[^ ^10^ ^]^ We sought to evaluate the performance of HN3‐Nb_6E_ labeled using 6E‐xlink in this context. HN3‐Nb_6E_ was subjected to sortagging conditions using a sacrificial triglycine nucleophile to remove the C‐terminal hexahistidine tag to avoid complications arising from low‐affinity complexation of some radiometals by His‐tags (Figure S8, Supporting Information). His‐tag‐free HN3‐Nb_6E_ was then labeled with DOTA‐6E‐xlink and purified by size exclusion chromatography.

**Figure 5:**
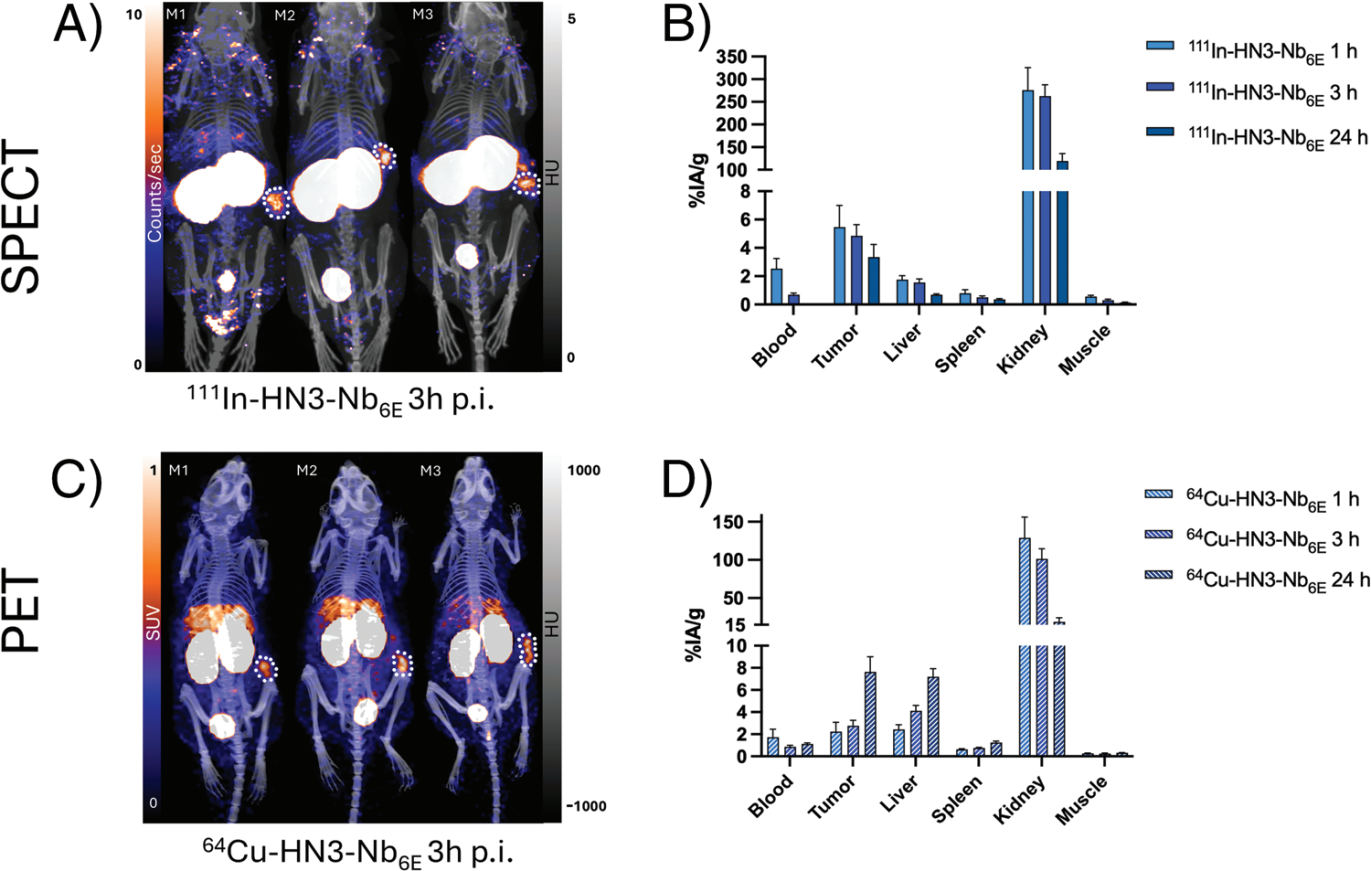
Radiolabeled HN3‐Nb_6E_ construct successfully images GPC3^+^ xenograft models of liver cancer. Mice bearing GPC3^+^ HepG2 tumors received ^111^In‐HN3‐Nb_6E_ or ^64^Cu‐HN3‐Nb_6E_ and underwent SPECT/CT A) with corresponding biodistribution studies B), or PET/CT C), with corresponding biodistribution studies D). Uptake was quantified as injected activity per gram tissue (% IA/g). Data show mean ± SD from analysis of 3–4 mice per group. Full biodistribution results, including the number of mice per group and analysis of other organs, can be found in Figures S12, S13 and Tables S1,S2 (Supporting Information).

DOTA‐conjugated HN3‐Nb_6E_ was successfully radiolabeled with either Indium‐111 (^111^In) (Figure S9, Supporting Information). We confirmed that radiolabeled conjugate maintained high affinity for GPC3 (Figure S10, Supporting Information). We also showed that HN3‐Nb_6E_ labeled with ^111^In ([^111^In]In‐HN3‐Nb_6E_) remained stable in human serum over 3 days (Figure S11, Supporting Information). Indium‐111‐ or Copper‐64‐radiolabeled HN3‐Nb_6E_ conjugates were intravenously injected into mice bearing HepG2 subcutaneous tumors for either single photon emission computed tomography (SPECT)/CT or PET/CT, respectively. We visualized tumor uptake by acquiring images of the probe distribution 3 h after injection (**Figure** **5**a,c). In addition, we performed a separate 12‐organ ex vivo biodistribution analysis, quantifying radio‐conjugate uptake into various tissues and organs at 1, 3, and 24 h (Figure 5b,d; Figures S12,S13, Tables S1,S2, Supporting Information).

Both imaging and biodistribution analyses demonstrated the accumulation of radioconjugates in tumors, suggesting that their target binding is preserved and successful application of the self‐labeling Nb module (Figure 5). SPECT/CT and PET/CT confirmed excellent tumor localization (Figure 5a,c), and we measured good tumor uptake (percent injected activity per gram, %IA/g) for ^111^In and ^64^Cu probes at 1 h (5.46 ± 1.54 %IA/g for ^111^In‐HN3‐Nb_6E_, 2.23 ± 0.85 %IA/g for ^64^Cu‐HN3‐Nb_6E_), 3 h (4.85 ± 0.80 %IA/g for ^111^In‐HN3‐Nb_6E_, 2.76 ± 0.49 %IA/g for ^64^Cu‐HN3‐Nb_6E_), and 24 h (3.34 ± 0.88 %IA/g for ^111^In‐HN3‐Nb_6E_, 7.64 ± 1.36 %IA/g for ^64^Cu‐HN3‐Nb_6E_) in biodistribution studies (Figure 5b,d). We observed a high uptake of both radioconjugates in kidneys, as is often observed for nanobody‐based imaging agents.^[^ ^5^ ^]^ Notably, while we did observe a higher level of uptake in tumors in animals receiving ^64^Cu‐HN3‐Nb_6E_ at 24 h, higher liver uptake was also observed, suggesting radioactive copper dissociated from DOTA and was transported into the tumor via copper transporters.^[^ ^26^ ^]^ It has been previously reported that the ^64^Cu‐DOTA complex can dissociate over time.^[^ ^27^ ^]^

To provide further evidence that HN3‐Nb_6E_ retained specificity for GPC3 we performed a biodistribution study with mice bearing a tumor formed from a HepG2 GPC3 knockout cell line (Figure S14, Table S3, Supporting Information) using ^111^In‐HN3‐Nb_6E_. Results confirmed minimal radio‐conjugate uptake (0.56 ± 0.07 IA/g at 3 h) in the GPC3 knockout tumors, relative to the levels of uptake observed in experiments with HepG2 wildtype tumors (4.85 ± 0.80% IA/g at 3 h) (Figure S14, Supporting Information). These studies confirm that our epitope‐based crosslinking strategy can be leveraged to yield effective tumor‐selective imaging agents.

## 3 Discussion

Here, we demonstrate that heterodimeric nanobody fusions can be expressed recombinantly, maintain native affinity toward cognate targets of interest, and can be modified with a variety of synthetic moieties using Nb_6E_ as a self‐labeling protein domain. This approach was effectively deployed to generate a new imaging agent that targets GPC3, which was used for the visualization of GPC3‐expressing xenograft models of liver cancer in mice. Although other methods have been reported for the site‐specific modification of Abs and Nbs at one or two sites,^[^ ^28^, ^29^, ^30^, ^31^ ^]^ the approach presented here provides more rapid labeling than enzymatic methods such as sortagging (see Figure S2, Supporting Information), which often requires overnight incubation.

There is substantial interest in the biomedical applications of Ab fragments, such as single‐domain antibodies.^[^ ^1^ ^]^ Nbs have proven valuable for bio‐engineering applications, including the generation of multi‐specific constructs, as their single domain architecture avoids difficulties related to pairing of Ab heavy and light chains for conventional Abs.^[^ ^32^ ^]^ Further, Nbs lack the constant (Fc) domain found in full‐sized antibodies, which can initiate antibody‐dependent cell‐mediated cytotoxicity responses that are unwanted in an imaging context. The small size of Nbs also facilitates rapid clearance from the bloodstream in humans and mice by renal filtration, especially compared to conventional Abs, which can circulate for days or weeks. Rapid clearance allows the use of Nbs as imaging agents with optimal signal‐to‐noise within hours instead of days, which aligns with existing clinical imaging workflows, encouraging many groups to explore this application of Nbs.^[^ ^33^ ^]^

It has become clear that non‐specific labeling methods can cause sub‐optimal or variable performance for Nbs in some contexts. Past work from our groups has shown that site‐specific labeling of a single‐domain antibody leads to improved tumor‐to‐background ratios.^[^ ^10^ ^]^ This realization has prompted the use of site‐specific labeling technologies to improve the performance and consistency of labeled Nbs. Some such approaches rely on the genetic incorporation of short sequences recognized by enzyme ligases such as Sortase A and AEPs. Enzymatic labeling allows for exquisite site‐specificity, but these reactions often require high concentrations (mm levels) of the probe used for labeling, complicating some applications. An elegant solution for labeling full‐sized Abs relies on proximity‐mediated labeling mediated by an electrophile‐linked peptide that binds to and labels the Ab constant region.^[^ ^34^ ^]^ However, this cannot be used with Nbs, which lack a constant region.

There are fewer examples of labeling Abs or Nbs through fusion with self‐labeling protein domains such as Halo‐tag (≈30 kDa),^[^ ^35^ ^]^ SNAP‐tag (≈20 kDa),^[^ ^36^ ^]^ or SpyCatcher (≈15 kDa).^[^ ^37^ ^]^ The SpyCatcher/SpyTag system, in which fragments of a split bacterial protein spontaneously reassemble and form a covalent bond, has been used to build a variety of multivalent antibody conjugates.^[^ ^38^, ^39^ ^]^ Protein engineering has allowed site‐specific modification of Abs with cytotoxic cargoes or fluorophores using SpyCatcher.^[^ ^40^, ^41^ ^]^ However, the bacterial origin of this protein poses the possibility of immunogenic responses when applied in vivo.^[^ ^42^ ^]^ Halo‐tag labeling is based on the chemistry of an archaeal dehalogenase enzyme that forms a covalent bond with alkyl halides at exceptionally rapid rates.^[^ ^23^ ^]^ Halo‐tag‐fused Abs have been applied as pre‐targeting reagents, in which the binding of Ab‐HaloTag fusion proteins is allowed to proceed in vivo before administration of labeled probes that react with HaloTag and allow detection. This approach has enabled the visualization of tumors bound by Ab‐HaloTag conjugates with high signal‐to‐noise ratios,^[^ ^43^ ^]^ but the non‐eukaryotic origin of HaloTag again raises immunogenicity concerns. The SNAP tag is derived from an engineered variant of human alkylguanine‐DNA transferase, which reacts with benzylguanine derivatives to form a covalent bond. SNAP‐tag has been used as a genetically encoded tag to track cellular localization in vivo, either using PET imaging^[^ ^44^ ^]^ or fluorescent methods.^[^ ^45^ ^]^

The self‐labeling domain based on Nb_6E_ described here possesses some favorable characteristics compared to previously reported self‐labeling domains. Unlike SpyCatcher and Halo‐tag, Nb_6E_ is derived from a mammalian source with homology to human immunoglobulin variable domains.^[^ ^21^ ^]^ Past work has demonstrated the humanization of Nbs,^[^ ^46^ ^]^ alleviating, to some extent, concerns about immunogenicity. The SNAP tag has not been used for the modification of Abs or Nbs applied in vivo, in contrast to the results reported here for Nb_6E_. Nb_6E_ is also smaller (≈12 kDa) than each of the three self‐labeling domains discussed above, allowing for improved tissue penetration for imaging or therapeutic uses. Several bivalent Nb fusion proteins have shown specialized and useful biological activities,^[^ ^47^, ^48^, ^49^ ^]^ further encouraging application.

The use of Nbs, including the heterobivalent Nb construct described here, offers promise for imaging liver cancer. Hepatocellular carcinoma (HCC), is the most common type of liver cancer and has a poor prognosis.^[^ ^50^ ^]^ Several studies have demonstrated that antibody conjugates that target GPC3, which is expressed in ≈75% of HCC, can be used to successfully engineer PET imaging and radiopharmaceutical therapy agents.^[^ ^3^, ^10^, ^17^, ^18^ ^]^ Efforts to use such imaging agents to assess tumor status following treatment are underway. Notably, past work using HN3‐based agents has demonstrated low levels of toxicity, encouraging further exploration of this area.^[^ ^10^, ^51^ ^]^

In summary, the application of the Nb_6E_ self‐labeling reaction in the context of heterodimeric Nb constructs offers an approach that is flexible, robust, and has several potential biomedical applications. Future avenues include additional exploration of such constructs for in vivo imaging, including pre‐targeting approaches,^[^ ^43^, ^52^ ^]^ and the labeling of T‐cells engineered for cancer immunotherapy.^[^ ^53^ ^]^ Rapid self‐labeling using a single domain building block derived from a mammalian source opens possibilities for translational bioengineering efforts.

## 4 Experimental Section

### Synthesis

Peptides were synthesized using solid‐phase peptide synthesis with Fmoc protection of backbone amines and standard side chain protecting groups. Crosslinking groups were attached using cysteine‐maleimide chemistry as previously described and illustrated in Supporting Methods (Supporting Information).^[^ ^16^ ^]^ Most labels (chelators, fluorophores, click handles, etc.) attached at the N‐terminus were attached via reaction with peptide still attached to the solid phase, with exceptions and details provided in Supporting Methods (Supporting Information). All peptidic compounds were purified with reverse‐phase HPLC, and compound identity was confirmed by mass spectrometry. Mass spectrometry characterization of purified compounds and purified proteins is summarized in Table S4 (Supporting Information) and Supporting Information. Synthetic details are described in Supporting Methods (Supporting Information).

### Nanobody Labeling Using Crosslinking Peptide Reagents

Conditions for individual labeling reactions vary slightly as described in figure captions. A generic reaction setup was described here. Dimeric nanobody (10–40 µm) in PBS was mixed with an excess (1.5–3 molar equivalents) of 6E‐xlinking peptide for 0.5–3 h at either 4 °C or room temperature. Crude reactions were analyzed by SDS‐PAGE or mass spectrometry to confirm conversion to product (see Supporting Information for mass spectrometry characterizations). For crosslinking reactions involving DOTA‐6E‐xlink, higher‐than‐expected molecular weights were observed in mass spectrometry analyses using some commercial buffers. In these cases, the exchange of proteins into buffers pretreated with metal chelating resin (Sigma–Aldrich, # C7901) before reaction with DOTA‐6E‐xlink provided reaction products of the expected molecular weight. Labeled dimeric nanobody products were purified using either a PD10 desalting column (Cytiva #17085101) or HiLoad 16/600 Superdex 200 pg column with an isocratic gradient of the buffer of choice (Flow rate 1 mL min^−1^). Crosslinking reactions were also successfully executed in tris‐buffered saline (pH 7.4) or ammonium acetate (pH 5.5) buffers. Labeling with azide‐6E‐xlink was further assessed by exposure to PEG_10KDa_‐DBCO (Vector Labs, CCT‐A119).

### Protein Labeling Using Sortase A and Click Chemistry

Purified heterodimeric Nb constructs were site‐specifically labeled using sortagging and purified as previously described^[^ ^22^ ^]^ or produced commercially using Chinese hamster ovary expression as detailed in Supporting Methods (Supporting Information).

### Nanobody Purification from CHO Cells

Large‐scale production of HN3‐Nb_6E_ was performed using the TurboCHO protein expression service from Genscript followed by purification using His tag Ni‐NTA affinity chromatography. Protein preparations provided by Genscript were analyzed using in‐house mass spectrometry and SDS‐PAGE characterizations.

### Gel Electrophoresis

SDS‐PAGE was run either using 15% acrylamide gels prepared in‐house or commercial gradient (4%–20%) gels (Biorad, #4561093). All samples were mixed with 4x Laemmli buffer (Biorad #1610747) modified with dithiothreitol (100 mm) and boiled before analysis. Gel‐based fluorescence imaging (when relevant) was performed using a Biorad ChemiDoc before staining with PAGE‐Blue (Thermo Fisher). Total protein staining following PAGE‐blue staining was also performed using Biorad ChemiDoc. Visualization of fluorophores was performed before staining with PAGE‐Blue using automated settings on Biorad ChemiDoc for the detection of fluorescein and tetramethylrhodamine. Uncropped gels are shown in Supporting Information.

### Cell Culture

Cell‐based experiments were run with the murine B Cell lymphoma line A20 (ATCC TIB‐208), HepG2 human hepatoblastoma (GPC3^+^ wildtype, and GPC3 knockout), A431 human epithelioid cancer cell lines, and A431‐GPC3+ cells, a transfected cell line engineered to overexpress GPC3.^[^ ^10^ ^]^ A20 was grown in RPMI1640 supplemented with 10% fetal bovine serum and 0.05 mm β‐mercaptoethanol and passaged every 3–5 days. All other cell lines were used within 10 passages and cultured in Dulbecco‐modified Eagle medium (Life Technologies) with 10% FetaPlex (Gemini Bio‐Products).

### Flow Cytometry

Staining was performed by incubation of cells in a buffer containing Nb constructs, followed by washing and staining with a secondary antibody to facilitate analysis by flow cytometry. Details are provided in Supporting Methods (Supporting Information).

### Biolayer Interferometry

HN3‐Nb_6E_ variants’ binding kinetics were evaluated using an Octet RED96e biolayer interferometry system (FortéBio), with methods previously described.^[^ ^10^ ^]^ Briefly, biotinylated human GPC3 was immobilized on streptavidin biosensor tips, and association and dissociation with various concentrations (25, 50 100, 200 nm) of HN3‐Nb_6E_ protein were measured.

### Radiolabeling and Characterization of ^111^In‐HN3‐Nb_6E_

Sodium acetate (53 µL, 0.4 m, pH 5) was added to a solution of ^111^In‐chloride (17 µL, 216 MBq, Cardinal Health). A solution of HN3‐Nb_6E_ nanobody in ammonium acetate (80 µL, 2.5 mg mL^−1^) was added to the mixture, and the reaction (pH 5) was heated for 1 h at 40 °C. The reaction was quenched with ethylenediaminetetraacetic acid (EDTA) (10 µL, 100 mm) and purified using a PD‐10 gel filtration column with phosphate‐buffered saline containing bovine serum albumin (1 mg mL^−1^) as the mobile phase. Radio‐instant thin‐layer chromatography with silica gel‐impregnated glass microfiber paper strips (Varian) was used to determine radiochemical yield and purity. This was done using a buffer containing EDTA (50 mm) and ammonium acetate (100 mm, pH 5.5) as the mobile phase. To calculate the percentage of total activity at the origin, a radio‐thin‐layer chromatography scanner was used (AR‐2000, Eckert‐Ziegler).

### Radiolabeling and Characterization of ^64^Cu‐HN3‐Nb_6E_

Gentisic acid (10 µL, 5 mg mL^−1^) was added to a solution of ^64^Cu‐chloride (5 µL, 129.5 MBq, University of Wisconsin Cyclotron) in hydrochloric acid (1 m). Ammonium acetate (30 µL, 1 m, pH 7) was then added to the mixture, followed by the HN3‐Nb_6E_ nanobody in sodium acetate (118 µL, 0.85 mg mL^−1^). The reaction (pH 7) was heated for 1 h at 40 °C and quenched with EDTA (10 µL, 100 mm). Purification was done, and radiochemical yield and purity were determined using the methods above.

### Murine Subcutaneous Xenograft Models and Animal Protocols

Female athymic nu/nu 8–10‐week‐old mice (NCI CCR Animal Resource Program/NCI Biological Testing Branch) were implanted with 5 × 10^6^ GPC3^+^ HepG2 or HepG2‐GPC3^‐^ (knockout) liver cancer cells in a 100 µL solution of Dulbecco modified Eagle medium (Gibco). Biodistribution and imaging experiments were performed once tumors reached ≈100 mm^3^. Mouse experiments were approved by the Institutional Animal Care and Use Committee at the National Institutes of Health under protocol ROB‐105.

### SPECT/CT Imaging

HepG2 tumor‐bearing mice (n = 4) were injected with 15.0 ± 0.041 MBq (405.5 ± 1.12 µCi,17.5 µg) of ^111^In‐HN3‐Nb_6E_ and imaged at 1, 3, and 24 h post‐injection using SPECT/CT (NanoSPECT/CT; Bioscan by Mediso). The mice were anesthetized using 2% isoflurane, and whole‐body CT scans (2 min, 45 kV, 177 µA), followed by SPECT scans (20 min) were acquired. MIM software (MIM Software Inc.) was used to analyze the images. Post 24‐h imaging, mice were euthanized by CO_2_ asphyxiation, and 12 tissues were collected. All samples were weighed and counted on a gamma counter (2480 Wizard^[^ ^3^ ^]^: Perkin Elmer Inc.), after which percentage injected activity per gram (%IA/g) was calculated (counts were converted to percentage injected activity (%IA) using a standard solution of known activity prepared from the injection solution, followed by dividing the activity of each organ by its weight).

### PET/CT Imaging

Mice bearing HepG2 xenografts (n = 3) were injected with 6.61 ± 0.19 MBq (178.7 ± 5.09 µCi, 10.5 µg) of ^64^Cu‐HN3‐Nb_6E_, and PET/CT scans (MRS*PET/CT; MR Solutions) were obtained at 1, 3, and 24 h post‐injection. 2% isoflurane was used to anesthetize the mice, and whole‐body CT scans (2.5 min, 60 kV, 300 µA), followed by static PET scans (10 min). Images were normalized, decay‐corrected, and dead‐time‐corrected and analyzed using MIM.

### Ex vivo Biodistribution Studies

Mice (n = 4) were injected via tail vein with either ^111^In or ^64^Cu labeled nanobody (0.548 ± 0.051 MBq (14.8 ± 1.39 µCi, 0.56 µg) and 3.83 ± 0.09 MBq (103.4 ± 2.47 µCi, 6 µg), respectively). Tissues were collected at 1, 3, and 24 h post‐injection following the protocol detailed in the previous section, and conjugate uptake was measured.

### Data Calculations and Display

Data were analyzed and prepared for display using GraphPad Prism v10, FlowJo v10.10, and MIM Software v7.3.5. Flow cytometry data were quantified by measuring median fluorescence of intensity (MFI) measurements using FlowJo software.

### Statistical Analysis

Pre‐processing (normalization) of data was performed as described in figure legends. Data with replicates are shown as mean ± SD with sample sizes (n) listed. For statistical testing, a 2‐tailed, unpaired, parametric Student *t*‐test (without *post hoc* adjustment) was used. Exact *p* values are listed where applicable. Statistical comparisons were performed using GraphPad Prism.

## Conflict of Interest

MH holds the patent (WO2012145469) assigned to the NIH for the HN3 single domain antibody specific for GPC3. The other authors declare no conflicts of interest.

## Supporting information



Supporting Information

## Data Availability

The data that support the findings of this study are available in the supplementary material of this article.

## References

[1] R. W. Cheloha , T. J. Harmand , C. Wijne , T. U. Schwartz , H. L. Ploegh , J. Biol. Chem. 2020, 295, 15307. 32868455 10.1074/jbc.REV120.012960 PMC7650250

[2] N. Dammes , D. Peer , Theranostics 2020, 10, 938. 31903161 10.7150/thno.37443 PMC6929980

[3] J. A. Carrasquillo , J. A. O'Donoghue , V. Beylergil , S. Ruan , N. Pandit‐Taskar , S. M. Larson , P. M. Smith‐Jones , S. K. Lyashchenko , N. Ohishi , T. Ohtomo , G. K. Abou‐Alfa , EJNMMI Res. 2018, 8, 20. 29508107 10.1186/s13550-018-0374-8 PMC5838028

[4] S. Tendler , M. P. Dunphy , M. Agee , J. O'Donoghue , R. G. Aly , N. J. Choudhury , A. Kesner , A. Kirov , A. Mauguen , M. K. Baine , H. Schoder , W. A. Weber , N. Rekhtman , S. K. Lyashchenko , L. Bodei , M. J. Morris , J. S. Lewis , C. M. Rudin , J. T. Poirier , Lancet Oncol. 2024, 25, 1015. 38950555 10.1016/S1470-2045(24)00249-3 PMC11656522

[5] T. J. Harmand , A. Islam , N. Pishesha , H. L. Ploegh , RSC Chem. Biol. 2021, 2, 685. 34212147 10.1039/d1cb00023c PMC8190910

[6] W. Wei , Z. T. Rosenkrans , J. Liu , G. Huang , Q.‐Y. Luo , W. Cai , Chem. Rev. 2020, 120, 3787. 32202104 10.1021/acs.chemrev.9b00738 PMC7265988

[7] B. Shuch , A. J. Pantuck , J.‐C. Bernhard , M. A. Morris , V. Master , A. M. Scott , C. Van Praet , C. Bailly , B. Önal , T. Aksoy , R. Merkx , D. M. Schuster , S. T. Lee , N. Pandit‐Taskar , A. C. Fan , P. Allman , K. Schmidt , L. Tauchmanova , M. Wheatcroft , C. Behrenbruch , C. R. W. Hayward , P. Mulders , Lancet Oncol. 2024, 25, 1277. 39270701 10.1016/S1470-2045(24)00402-9

[8] P. Adumeau , S. K. Sharma , C. Brent , B. M. Zeglis , Mol. Imaging Biol. 2016, 18, 1. 10.1007/s11307-015-0919-4 PMC4722084 26754790

[9] P. Debie , N. Devoogdt , S. Hernot , Antibodies 2019, 8, 12. 31544818 10.3390/antib8010012 PMC6640687

[10] S. Fayn , A. P. King , N. T. Gutsche , Z. Duan , J. Buffington , C. P. Olkowski , Y. Fu , J. Hong , D. Sail , K. E. Baidoo , R. E. Swenson , R. W. Cheloha , M. Ho , P. Choyke , F. Escorcia , J. Nucl. Med. 2023, 64, 1017. 36997331 10.2967/jnumed.122.265171 PMC10315705

[11] P. Adumeau , S. K. Sharma , C. Brent , B. M. Zeglis , Mol. Imaging Biol. 2016, 18, 153. 26754791 10.1007/s11307-015-0920-y PMC4842939

[12] N. Pishesha , J. R. Ingram , H. L. Ploegh , A. Sortase , Annu. Rev. Cell Dev. Biol. 2018, 34, 163. 30110557 10.1146/annurev-cellbio-100617-062527

[13] F. B. H. Rehm , T. J. Tyler , J. Xie , K. Yap , T. Durek , D. J. Craik , J. Chem. Biol. 2021, 22, 2079. 10.1002/cbic.202100071 33687132

[14] F. Basuli , J. Shi , E. Lindberg , S. Fayn , W. Lee , M. Ho , D. A. Hammoud , R. W. Cheloha , R. E. Swenson , F. E. Escorcia , Bioconjug. Chem. 2024, 35, 1335. 39172920 10.1021/acs.bioconjchem.4c00264 PMC12781894

[15] B. M. Zeglis , C. B. Davis , R. Aggeler , H. C. Kang , A. Chen , B. J. Agnew , J. S. Lewis , Bioconjug. Chem. 2013, 24, 1057. 23688208 10.1021/bc400122c PMC3714844

[16] C. C. Cabalteja , S. Sachdev , R. W. Cheloha , Bioconjug. Chem. 2022, 33, 1867. 36107739 10.1021/acs.bioconjchem.2c00334 PMC10200341

[17] M. M. Bell , N. T. Gutsche , A. P. King , K. E. Baidoo , O. J. Kelada , P. L. Choyke , F. E. Escorcia , Molecules 2020, 26, 4. 33374953 10.3390/molecules26010004 PMC7792624

[18] K. P. Labadie , D. K. Hamlin , A. Kenoyer , S. K. Daniel , A. F. Utria , A. D. Ludwig , H. L. Kenerson , L. Li , J. G. Sham , D. L. Chen , J. J. Orozco , R. S. Yeung , C. Orvig , Y. Li , D. S. Wilbur , J. O. Park , J. Nucl. Med. 2022, 63, 1033. 34772791 10.2967/jnumed.121.262562 PMC9258570

[19] T. Pleiner , M. Bates , D. Görlich , J. Cell Biol. 2018, 217, 1143. 29263082 10.1083/jcb.201709115 PMC5839796

[20] M. Feng , W. Gao , R. Wang , W. Chen , Y.‐G. Man , W. D. Figg , X. W. Wang , D. S. Dimitrov , M. Ho , Proc. Natl. Acad. Sci. 2013, 110, e1083. 23471984 10.1073/pnas.1217868110 PMC3607002

[21] J. Ling , R. W. Cheloha , N. McCaul , Z. J. Sun , G. Wagner , H. L. Ploegh , Mol. Immunol. 2019, 114, 513. 31518855 10.1016/j.molimm.2019.08.008 PMC6774866

[22] C. C. Cabalteja , S. Sachdev , R. W. Cheloha , ACS Chem. Biol. 2022, 17, 2296. 35930411 10.1021/acschembio.2c00407 PMC10200313

[23] J. Wilhelm , S. Kühn , M. Tarnawski , G. Gotthard , J. Tünnermann , T. Tänzer , J. Karpenko , N. Mertes , L. Xue , U. Uhrig , J. Reinstein , J. Hiblot , K. Johnsson , Biochemistry 2021, 60, 2560. 34339177 10.1021/acs.biochem.1c00258 PMC8388125

[24] R. W. Cheloha , Z. Li , D. Bousbaine , A. W. Woodham , P. Perrin , J. Volarić , H. L. Ploegh , ACS Chem. Biol. 2019, 14, 1836. 31348637 10.1021/acschembio.9b00493 PMC7408311

[25] W. Gao , Z. Tang , Y.‐F. Zhang , M. Feng , M. Qian , D. S. Dimitrov , M. Ho , Nat. Commun. 2015, 6, 6536. 25758784 10.1038/ncomms7536 PMC4357278

[26] R. J. Stockert , P. S. Grushoff , A. G. Morell , G. E. Bentley , H. A. O'Brien , I. H. Scheinberg , I. Sternlieb , Hepatology 1986, 6, 60. 3002940 10.1002/hep.1840060112

[27] C. J. Anderson , R. Ferdani , Cancer Biother. Radiopharm. 2009, 24, 379. 19694573 10.1089/cbr.2009.0674 PMC2794299

[28] M. D. Lee , W. Y. Tong , T. Nebl , L. A. Pearce , T. M. Pham , A. Golbaz‐Hagh , S. Puttick , S. Rose , T. E. Adams , C. C. Williams , Bioconjug. Chem. 2019, 30, 2539. 31560523 10.1021/acs.bioconjchem.9b00639

[29] F. B. H. Rehm , T. J. Harmand , K. Yap , T. Durek , D. J. Craik , H. L. Ploegh , J. Am. Chem. Soc. 2019, 141, 17388. 31573802 10.1021/jacs.9b09166 PMC7372569

[30] T. J. Harmand , D. Bousbaine , A. Chan , X. Zhang , D. R. Liu , J. P. Tam , H. L. Ploegh , Bioconjug. Chem. 2018, 29, 3245. 30231608 10.1021/acs.bioconjchem.8b00563 PMC6429940

[31] T. Journeaux , M. B. Geeson , T. V. Murray , M. A. Papworth , M. Gothard , A. V. Vasco , J. G. Kettle , G. J. L. Bernardes , Angew. Chem., Int. Ed. 2024, 64, e202417620. 10.1002/anie.202417620 PMC11773117 39423140

[32] J. Wang , G. Kang , H. Yuan , X. Cao , H. Huang , A. de Marco , Front. Immunol. 2022, 12, 838082. 35116045 10.3389/fimmu.2021.838082 PMC8804282

[33] E. R. Verhaar , A. W. Woodham , H. L. Ploegh , Semin. Immunol. 2021, 52, 101425. 33272897 10.1016/j.smim.2020.101425 PMC8164649

[34] D. Yuan , Y. Zhang , K. H. Lim , S. K. P. Leung , X. Yang , Y. Liang , W. C. Y. Lau , K. T. Chow , J. Xia , J. Am. Chem. Soc. 2022, 144, 18494. 36167521 10.1021/jacs.2c07594

[35] G. V. Los , L. P. Encell , M. G. McDougall , D. D. Hartzell , N. Karassina , C. Zimprich , M. G. Wood , R. Learish , R. F. Ohana , M. Urh , D. Simpson , J. Mendez , K. Zimmerman , P. Otto , G. Vidugiris , J. Zhu , A. Darzins , D. H. Klaubert , R. F. Bulleit , K. V. H. A. Wood , ACS Chem. Biol. 2008, 3, 373. 18533659 10.1021/cb800025k

[36] A. Keppler , S. Gendreizig , T. Gronemeyer , H. Pick , H. Vogel , K. Johnsson , Nat. Biotechnol. 2003, 21, 86. 12469133 10.1038/nbt765

[37] B. Zakeri , J. O. Fierer , E. Celik , E. C. Chittock , U. Schwarz‐Linek , V. T. Moy , M. Howarth , Proc. Natl. Acad. Sci. USA 2012, 109, E690. 22366317 10.1073/pnas.1115485109 PMC3311370

[38] M. K. Alam , C. Gonzalez , W. Hill , A. El‐Sayed , H. Fonge , K. Barreto , C. R. Geyer , J. Chem. Biol. 2017, 18, 2217. 10.1002/cbic.201700411 28891272

[39] C. Hentrich , S.‐J. Kellmann , M. Putyrski , M. Cavada , H. Hanuschka , A. Knappik , F. Ylera , Cell Chem. Biol. 2021, 28, 813. 33529581 10.1016/j.chembiol.2021.01.011

[40] V. Siegmund , B. Piater , B. Zakeri , T. Eichhorn , F. Fischer , C. Deutsch , S. Becker , L. Toleikis , B. Hock , U. A. K. Betz , H. Kolmar , Sci. Rep. 2016, 6, 39291. 27982100 10.1038/srep39291 PMC5159917

[41] M. K. Alam , A. El‐Sayed , K. Barreto , W. Bernhard , H. Fonge , C. R. Geyer , Mol. Imaging Biol. 2019, 21, 54. 29948640 10.1007/s11307-018-1222-y

[42] Z. Liu , H. Zhou , W. Wang , W. Tan , Y.‐X. Fu , M. Zhu , Sci. Rep. 2014, 4, 7266. 25434527 10.1038/srep07266 PMC4248271

[43] J. C. Knight , M. Mosley , H. T. Uyeda , M. Cong , F. Fan , S. Faulkner , B. Cornelissen , Mol. Pharm. 2017, 14, 2307. 28505463 10.1021/acs.molpharmaceut.7b00172 PMC5499097

[44] X. Li , X. Yang , Z. Li , X. Zheng , Y.‐J. Peng , W. Lin , L. Zhou , D. Cao , M. Situ , Q. Tu , H. Huang , W. Fan , G. Feng , X. Zhang , ACS Omega 2022, 7, 7550. 35284707 10.1021/acsomega.1c05856 PMC8908366

[45] G. Yang , F. de Castro Reis , M. Sundukova , S. Pimpinella , A. Asaro , L. Castaldi , L. Batti , D. Bilbao , L. Reymond , K. Johnsson , P. A. Heppenstall , Nat. Methods 2015, 12, 137. 25486061 10.1038/nmeth.3207

[46] Z. Sang , Y. Xiang , I. Bahar , Y. Shi , Struct. Lond. Engl. 2022, 30, 418. 10.1016/j.str.2021.11.006 PMC11698024 34895471

[47] S. Sachdev , B. A. Creemer , T. J. Gardella , R. W. Cheloha , Nat. Commun. 2024, 15, 4687. 38824166 10.1038/s41467-024-49068-5 PMC11144202

[48] N. Braga Emidio , R. W. Cheloha , Protein Sci. 2024, 33, e4866. 38088474 10.1002/pro.4866 PMC10806929

[49] N. Braga Emidio , B. M. Small , A. R. Keller , R. W. Cheloha , L. M. Wingler , Mol. Pharmacol. 2023, 105, 260. 10.1124/molpharm.123.000797 PMC10877709 38164609

[50] SEER*Explorer: An Interactive Website for SEER Cancer Statistics [Internet]. Surveillance Research Program, National Cancer Institute, https://Seer.Cancer.Gov/Statistics‐Network/Explorer/ (Accessed: November 2024).

[51] B. D. Fleming , D. J. Urban , M. D. Hall , T. Longerich , T. F. Greten , I. Pastan , M. Ho , Hepatol. Baltim. Md 2020, 71, 1696. 10.1002/hep.30949 PMC7069773 31520528

[52] B. E. Cook , P. Adumeau , R. Membreno , K. E. Carnazza , C. Brand , T. Reiner , B. J. Agnew , J. S. Lewis , B. M. Zeglis , Bioconjug. Chem. 2016, 27, 1789. 27356886 10.1021/acs.bioconjchem.6b00235 PMC5102008

[53] A. R. Sutherland , M. N. Owens , C. R. Geyer , Int. J. Mol. Sci. 2020, 21, 7222. 33007850 10.3390/ijms21197222 PMC7582510

