# Supplementary material for: Generation of Site‐Specifically Labeled Affinity Reagents via Use of a Self‐Labeling Single Domain Antibody
Source: Adv Sci (Weinh). 2025 Feb 18;12(14):2417160. doi: 10.1002/advs.202417160 (PMC11984916; doi:10.1002/advs.202417160)
Supplement: Supplementary file 1 — Supporting Information [file ADVS-12-2417160-s001.pdf]

ADVANCED SCIENCE
Open Access

Supporting Information

for *Adv. Sci.*, DOI 10.1002/advs.202417160

Generation of Site-Specifically Labeled Affinity Reagents via Use of a Self-Labeling Single Domain Antibody

Stanley Fayn, Swarnali Roy, Chino C. Cabalteja, Woonghee Lee, Hima Makala, Kwamena Baidoo, Divya Nambiar, Julia Sheehan-Klenk, Joon-Yong Chung, Jesse Buffington, Mitchell Ho, Freddy E. Escorcia and Ross W. Cheloha**

Supporting Information: Generation of Site-Specifically Labeled Affinity Reagents via Use of a Self-Labeling Single Domain Antibody

Supporting Tables.

Supporting Table S1. Tabulation of biodistribution data for [^{111}In]-DOTA-HN3Nb$_{6E}$ in mice with HepG2 tumors expressing GPC3. Data correspond to the graphic shown in Figure 5b and Supporting Figure 8.

	**[^{111}In]-DOTA-HN3Nb$_{6E}$ 1h**			**[^{111}In]-DOTA-HN3Nb$_{6E}$ 3h**			**[^{111}In]-DOTA-HN3Nb$_{6E}$ 24h**		
	Mean	SD	N	Mean	SD	N	Mean	SD	N
**Blood**	2.53	0.71	4	0.69	0.13	4	0.02	0.01	4
**HepG2 Tumor**	5.46	1.54	4	4.85	0.80	4	3.34	0.88	4
**Heart**	1.24	0.28	4	0.39	0.07	4	0.18	0.07	4
**Lungs**	2.28	0.81	4	0.96	0.12	4	0.38	0.05	4
**Liver**	1.76	0.28	4	1.55	0.25	4	0.68	0.06	4
**Spleen**	0.8	0.24	4	0.5	0.11	4	0.34	0.06	4
**Kidney**	275.79	49.76	4	262.77	25.06	4	118.85	16.29	4
**Stomach**	0.59	0.27	4	0.34	0.09	4	0.1	0.02	4
**Small Intestine**	0.85	0.23	4	0.36	0.05	4	0.13	0.03	4
**Large Intestine**	0.69	0.15	4	0.5	0.09	4	0.19	0.14	4
**Muscle**	0.56	0.09	4	0.3	0.08	4	0.13	0.02	4
**Femur**	0.43	0.06	4	0.33	0.05	4	0.18	0.04	4

Supporting Table S2. Tabulation of biodistribution data for [^{64}Cu]-DOTA-HN3Nb$_{6E}$ in mice with HepG2 tumors expressing GPC3. Data correspond to the graphic shown in Figure 5d and Supporting Figure 9.

	**[^{64}Cu]-DOTA-HN3Nb$_{6E}$ 1h**			**[^{64}Cu]-DOTA-HN3Nb$_{6E}$ 3h**			**[^{64}Cu]-DOTA-HN3Nb$_{6E}$ 24h**		
	**Mean**	**SD**	**N**	**Mean**	**SD**	**N**	**Mean**	**SD**	**N**
**Blood**	1.72	0.73	4	0.86	0.13	4	1.12	0.09	3
**HepG2 Tumor**	2.23	0.85	4	2.76	0.49	4	7.64	1.36	3
**Heart**	0.79	0.18	4	0.69	0.02	4	1.75	0.20	3
**Lungs**	1.39	0.36	4	1.45	0.10	4	2.86	0.36	3
**Liver**	2.43	0.43	4	4.12	0.49	4	7.2	0.74	3
**Spleen**	0.63	0.06	4	0.76	0.06	4	1.25	0.13	3
**Kidney**	128.89	27.26	4	101.3	13.27	4	18.78	5.38	3
**Stomach**	0.38	0.18	4	0.69	0.14	4	1.15	0.69	3
**Small Intestine**	0.81	0.18	4	1.8	0.72	4	2.3	0.38	3
**Large Intestine**	0.46	0.16	4	0.67	0.08	4	3.96	0.61	3
**Muscle**	0.27	0.04	4	0.25	0.06	4	0.32	0.03	3
**Femur**	0.46	0.10	4	0.46	0.11	4	0.81	0.25	3

Supporting Table S3. Tabulation of biodistribution data for [^{111}In]-DOTA-HN3Nb$_{6E}$ in mice with HepG2 tumors lacking GPC3. Data correspond to the graphic shown in Supporting Figure 10.

	[^{111}In]-DOTA-HN3Nb$_{6E}$ 1h			[^{111}In]-DOTA-HN3Nb$_{6E}$ 3h			[^{111}In]-DOTA-HN3Nb$_{6E}$ 24h		
	**Mean**	**SD**	**N**	**Mean**	**SD**	**N**	**Mean**	**SD**	**N**
**Blood**	2	0.56	4	0.66	0.23	4	0.05	0.02	4
**HepG2 GPC3^{-} Tumor**	1.03	0.29	4	0.56	0.07	4	0.43	0.12	4
**Heart**	1.23	0.41	4	0.48	0.12	4	0.26	0.06	4
**Lungs**	1.83	0.64	4	1.18	0.30	4	0.56	0.11	4
**Liver**	1.32	0.21	4	1.46	0.26	4	1.06	0.18	4
**Spleen**	0.71	0.17	4	0.54	0.05	4	0.47	0.10	4
**Kidney**	144.5	27.77	4	168.19	10.19	4	161.51	21.68	4
**Stomach**	0.31	0.09	4	0.18	0.05	4	0.15	0.02	4
**Small Intestine**	0.67	0.16	4	0.27	0.05	4	0.2	0.07	4
**Large Intestine**	0.38	0.05	4	0.36	0.10	4	0.24	0.07	4
**Muscle**	0.33	0.07	4	0.21	0.04	4	0.18	0.03	4
**Femur**	0.43	0.06	4	0.32	0.05	4	0.42	0.15	4

Supporting Table S4: Tabulation of mass spectrometry characterization data. Mass spectra are found below.

**Peptides**	**M/z calc**	**M/z obs**
FAM 6E C5	1860.8	1861.8
FAM-6E-C5 xlink	2192.9	2193.9
Bio-6E-C5	1898.9	1899.9
Bio-6E-C5 xlink	2231	2230
DOTA-Ahx-6E-C5	2002	2003
DOTA-Ahx-6E-C5-xlink	2334.1	2334.2
C16-6E-C5	1741	1742
C16-6E-C5-xlink	2073	2073
DFO-6E-C5	2255.1	2255
DFO-6E-C5-xlink	2608.2 (+Na)	2610.2 (+Na)
Azide-6E-C5	1698.8	1698.8
Azide-6E-C5-xlink	2030.9	2031.9
Halo-6E-C5	1805.8	1806.8
Halo-6E-C5-xlink	2137.9	2138.9
AZDye555-6E-xlink	3120.2	3121.2

Supporting Figures.

FAM-6E-C5

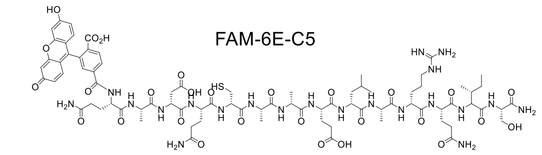

Chemical Formula: $C_{81}H_{113}N_{21}O_{28}S$
Exact Mass: 1859.78
Molecular Weight: 1860.97

FAM-6E-xlink

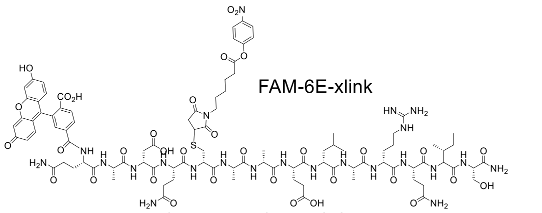

Chemical Formula: $C_{97}H_{129}N_{23}O_{34}S$
Exact Mass: 2191.88
Molecular Weight: 2193.29

Biotin-6E-C5

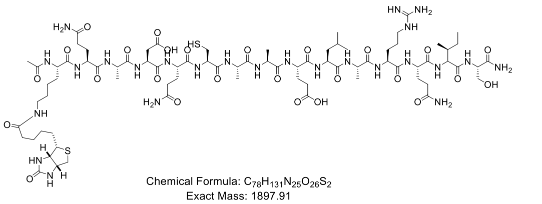

Chemical Formula: $C_{78}H_{131}N_{25}O_{26}S_2$
Exact Mass: 1897.91
Molecular Weight: 1899.17

Biotin-6E-xlink

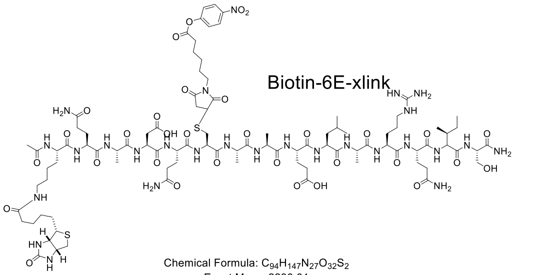

Chemical Formula: $C_{94}H_{147}N_{27}O_{32}S_2$
Exact Mass: 2230.01
Molecular Weight: 2231.49

DOTA-6E-C5

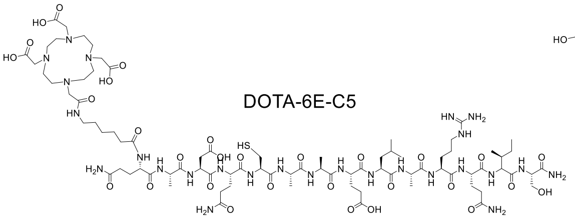

Chemical Formula: $C_{82}H_{140}N_{26}O_{30}S$
Exact Mass: 2000.99
Molecular Weight: 2002.23

DOTA-6E-xlink

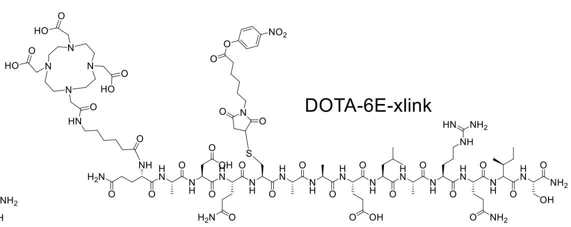

Exact Mass: 2333.10
Molecular Weight: 2334.55

C16-6E-C5

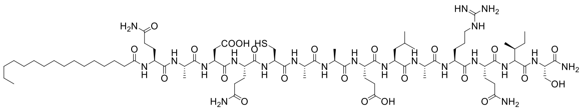

Chemical Formula: $C_{76}H_{133}N_{21}O_{23}S$
Exact Mass: 1739.96
Molecular Weight: 1741.08

C16-6E-xlink

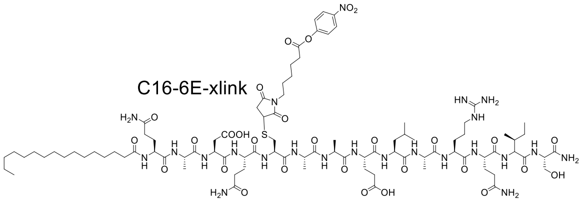

Chemical Formula: $C_{92}H_{149}N_{23}O_{29}S$
Exact Mass: 2072.06
Molecular Weight: 2073.40

DFO-6E-C5

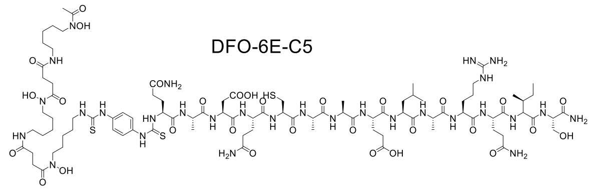

Chemical Formula: $C_{93}H_{155}N_{29}O_{30}S_3$
Exact Mass: 2254.07
Molecular Weight: 2255.62

DFO-6E-xlink

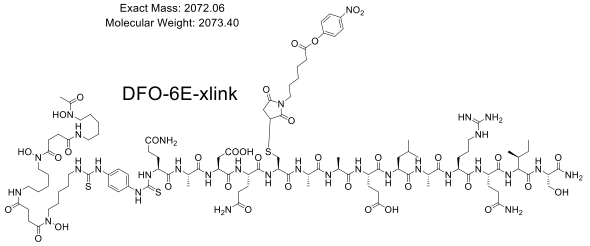

Chemical Formula: $C_{109}H_{171}N_{31}O_{36}S_3$
Exact Mass: 2586.17
Molecular Weight: 2587.93

Azide-6E-C5

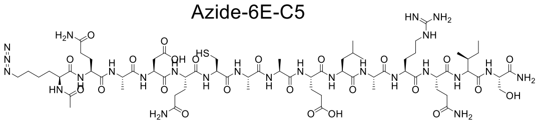

Chemical Formula: $C_{68}H_{115}N_{25}O_{24}S$
Exact Mass: 1697.83
Molecular Weight: 1698.88

Azide-6E-xlink

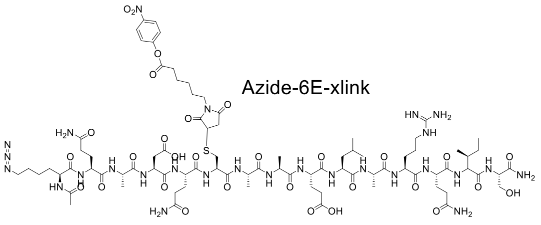

Chemical Formula: $C_{84}H_{131}N_{27}O_{30}S$
Exact Mass: 2029.93
Molecular Weight: 2031.19

AZdye555-6E-xlink

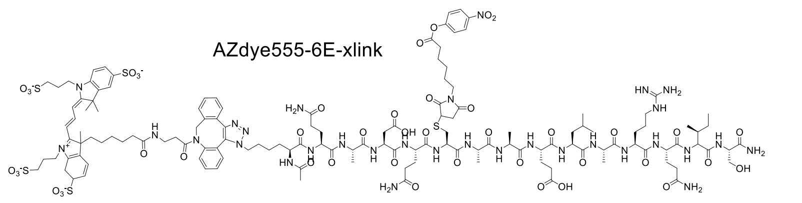

Chemical Formula: $C_{136}H_{188}N_{31}O_{44}S_5^{3-}$
Exact Mass: 3119.20
Molecular Weight: 3121.47

Halo-6E-C5

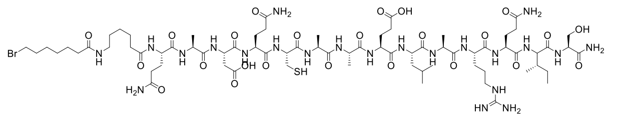

Chemical Formula: $C_{73}H_{125}BrN_{22}O_{24}S$
Exact Mass: 1804.81
Molecular Weight: 1806.90

Halo-6E-xlink

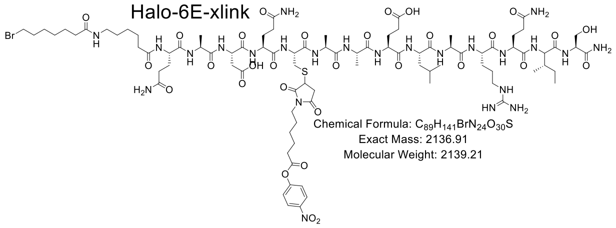

Chemical Formula: $C_{89}H_{141}BrN_{24}O_{30}S$
Exact Mass: 2136.91
Molecular Weight: 2139.21

Supporting Figure S1: Structures of synthesized 6E-xlink analogues. Mass spectrometry characterization is found below**.**

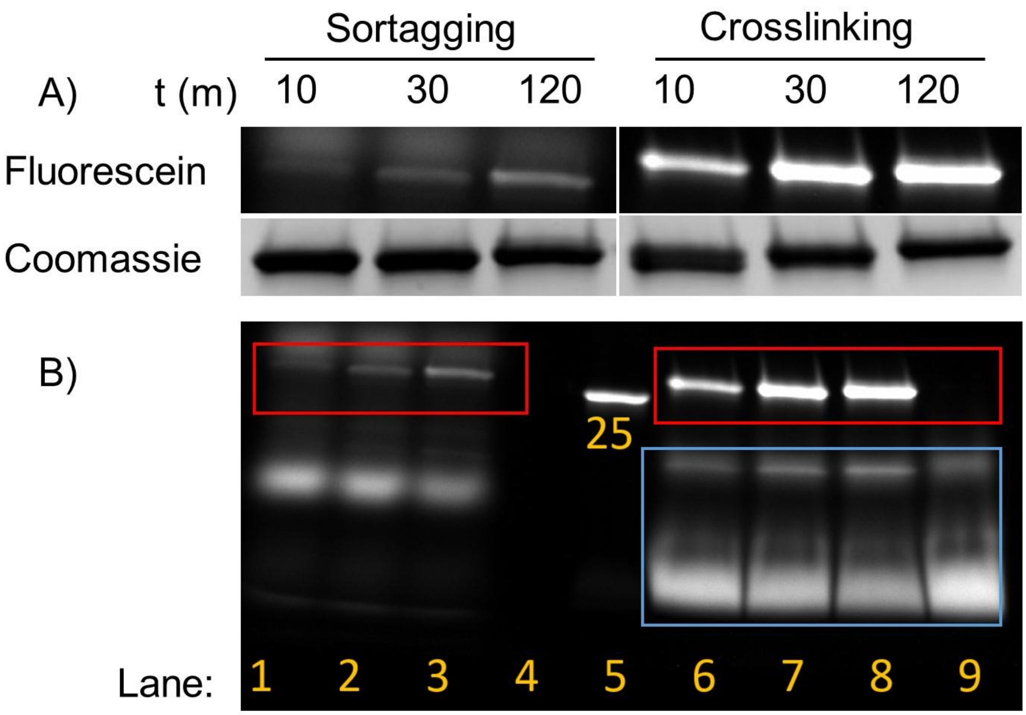

Lane#1 HN3-Nb_{6E}+G_3-FAM 10 min
Lane#2 HN3-Nb_{6E}+G_3-FAM 30 min
Lane#3 HN3-Nb_{6E}+G_3-FAM; 120 m
Lane#4 HN3-Nb_{6E}
Lane#5 Ladder
Lane#6 HN3-Nb_{6E}+FAM-6E-xlink 10 min
Lane#7 HN3-Nb_{6E}+FAM-6E-xlink 30 min
Lane#8 HN3-Nb6E+FAM-6E-xlink 120 m
Lane#9 FAM-6E--xlink alone

Supporting Figure S2: Comparison of the kinetics of labeling HN3-Nb_{6E} using Sortase A- or 6E-xlink peptide-based methods. A) A labeling reaction consisting of HN3-Nb_{6E} (10 µM) and 1.5 equivalents of fluorescein-labeled peptide (G_3-fluorescein or FAM-6E-xlink; 15 µM) were mixed in PBS followed by addition of Sortase 7M (20 µM) for sortagging reactions. Reactions were incubated for the indicated durations prior to the addition of SDS-PAGE sample buffer containing DTT to quench further reactivity. Reactions were then resolved using electrophoresis on a 4-20% acrylamide gradient gel (Biorad). The gel was first scanned to detect fluorescein fluorescence, followed by total protein staining (Coomassie). Panel A shows cropped fragments of gel images. **B)** Expanded section of fluorescence scan of section shown in panel A. The bands in the red rectangle correspond to a fluorophore-labeled HN3-Nb_{6E} whereas the bands in the blue rectangle correspond to a species of FAM-6E-xlink peptide that shows an unexpectedly high apparent molecular weight. Uncropped gel images (fluorescence scan and Coomassie stain) are shown below.

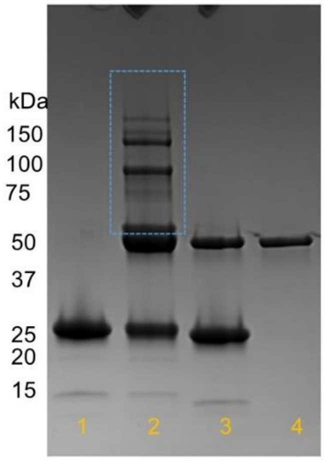

Lanes:
1 – HN3-Nb$_{6E}$ + Halo-6E-xlink
2 – HN3-Nb$_{6E}$ + Halo-6E-xlink + Halo-GST
3 – HN3-Nb$_{6E}$ + Halo-GST
4 – Halo-GST

Supporting Figure S3: Assessment of multiple labeling reactions mediated by 6E-xlinking and Halo-tag reactivity. Dimeric nanobody (HN3-Nb$_{6E}$, 40 µM) was incubated in PBS with halo-6E-xlink (60 µM) for 30 m at room temperature. After incubation, HaloTag-GST protein (50 µM, Promega, G4491) was added for an additional 30 m at which time SDS-PAGE sample buffer with dithiothreitol was added. Samples were analyzed by SDS-PAGE as described in Methods. All bands within the blue dashed box correspond to crosslinked products formed exclusively upon mixing of all three reaction components. The uncropped gel is shown below.

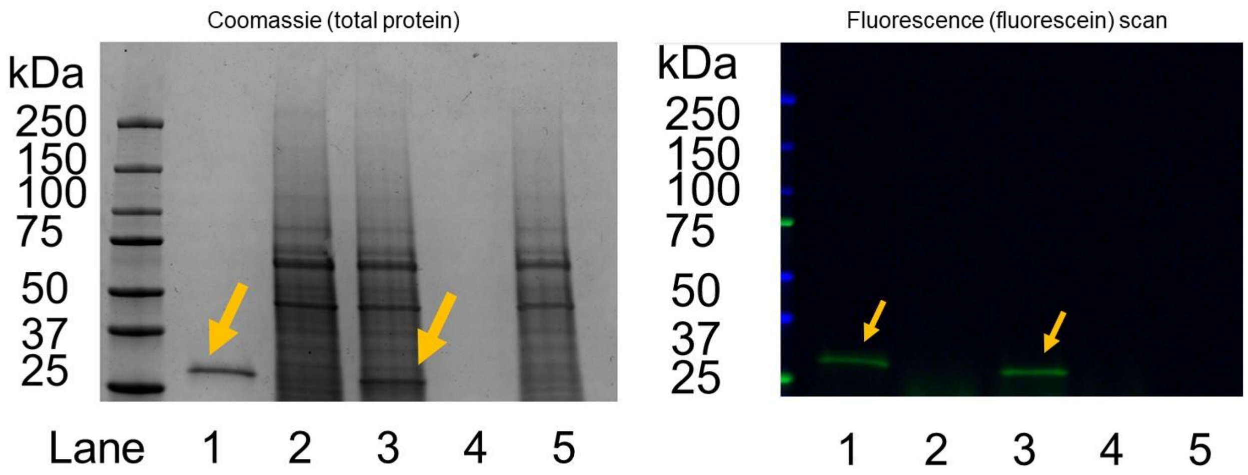

Lane:
1: HN3-Nb_{6E} + FAM-6E-xlink
2: Lysate+ FAM-6E-xlink
3: HN3-Nb6E +Lysate + FAM-6E-xlink
4: FAM-6E-xlink only
5: Lysate only

Supporting Figure S4: Specific labeling of HN3-Nb_{6E} by FAM-6E-xlink in the presence of cell lysate. HN3-Nb_{6E} (10 µM) was mixed with FAM-6E-xlink (15 µM) in the presence or absence of 25 µg of cell lysate (from wild type HEK293 cells) for 60 m at 25° C. Reactions were quenched through the addition of SDS-PAGE sample buffer with DTT (100 mM) and resolved on a 4-20% gradient acrylamide gel (Biorad). The gel was imaged using fluorescence scanning to detect fluorescein (right) and Coomassie to detect total protein (left). The left and right images come from the same imaged with different methods. Orange arrows point to bands corresponding to HN3-Nb_{6E}. Uncropped gel images are shown below.

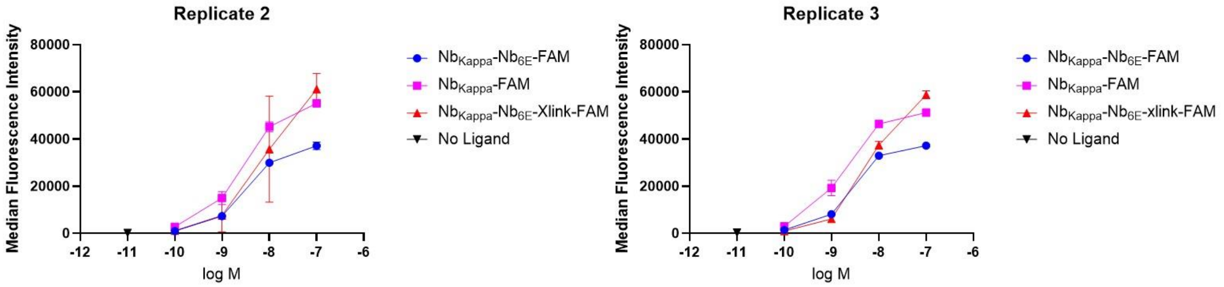

Supporting Figure S5: Quantified dose-response for the binding of labeled dimeric or monomeric Nb constructs to the A20 cell line. Binding assessments were performed using flow cytometry as described in Methods. Data shown here are independent replicate experiments analogous to those shown in main Figure 4. Data points correspond to mean ± SD from technical duplicate measurements.

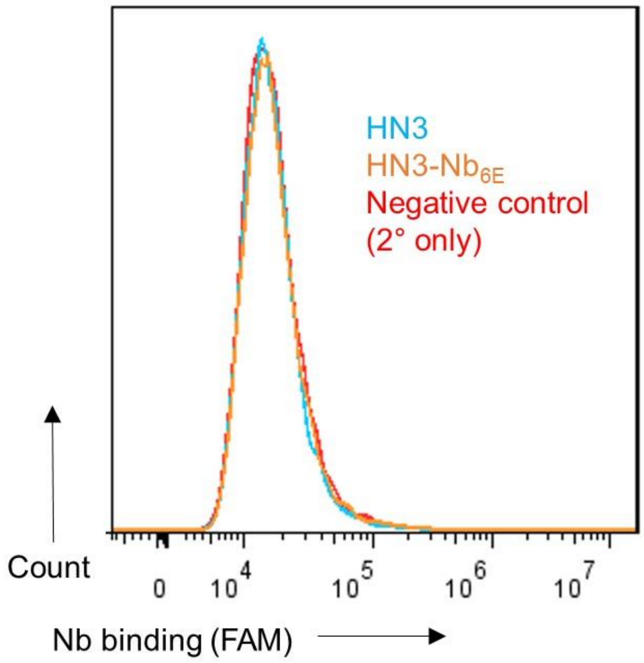

Supporting Figure S6: Analysis of dimeric nanobody specificity for GPC3 using flow cytometry. Histogram showing binding of dimeric or monomeric Nb constructs (1 µM) to A431 cells not expressing GPC3. Binding was detected with an anti-His tag secondary (2°) antibody as described in Supporting Methods.

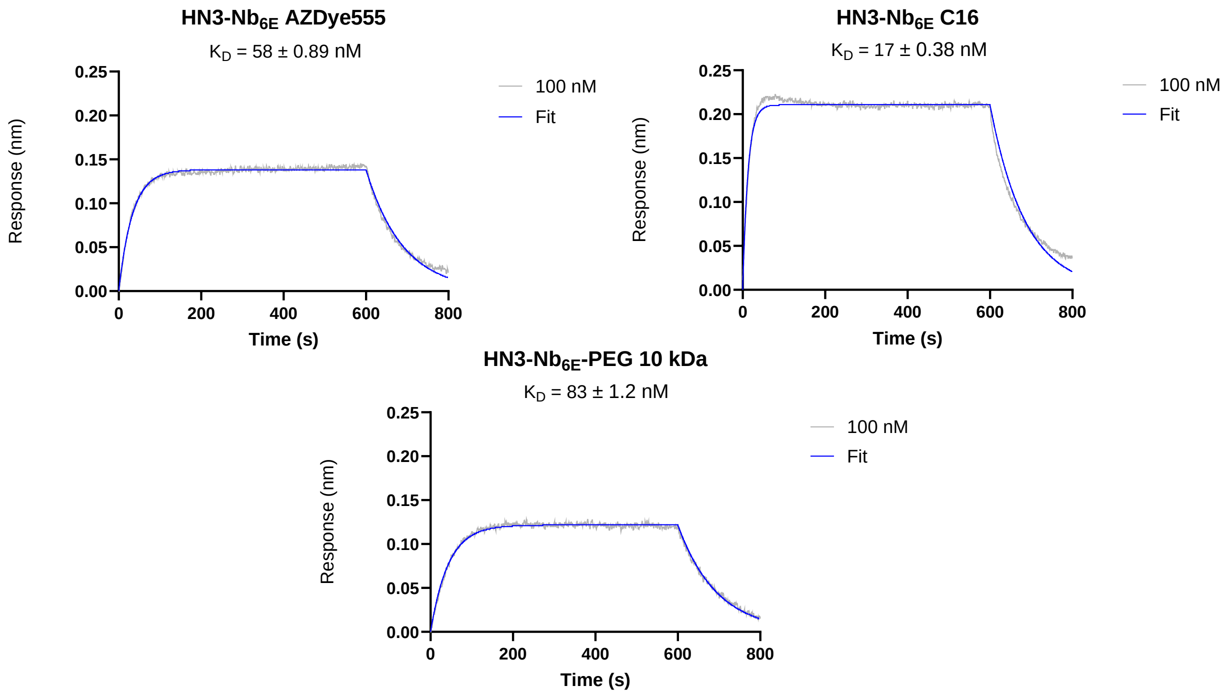

Supporting Figure S7. Assessment of the binding of HN3-Nb$_{6E}$ conjugates produced by crosslinking chemistry to GPC3 using biolayer interferometry analysis. Octet binding experiments were performed as described in Methods. Gray lines correspond to experimental data and blue lines to the experimental models fit to this data.

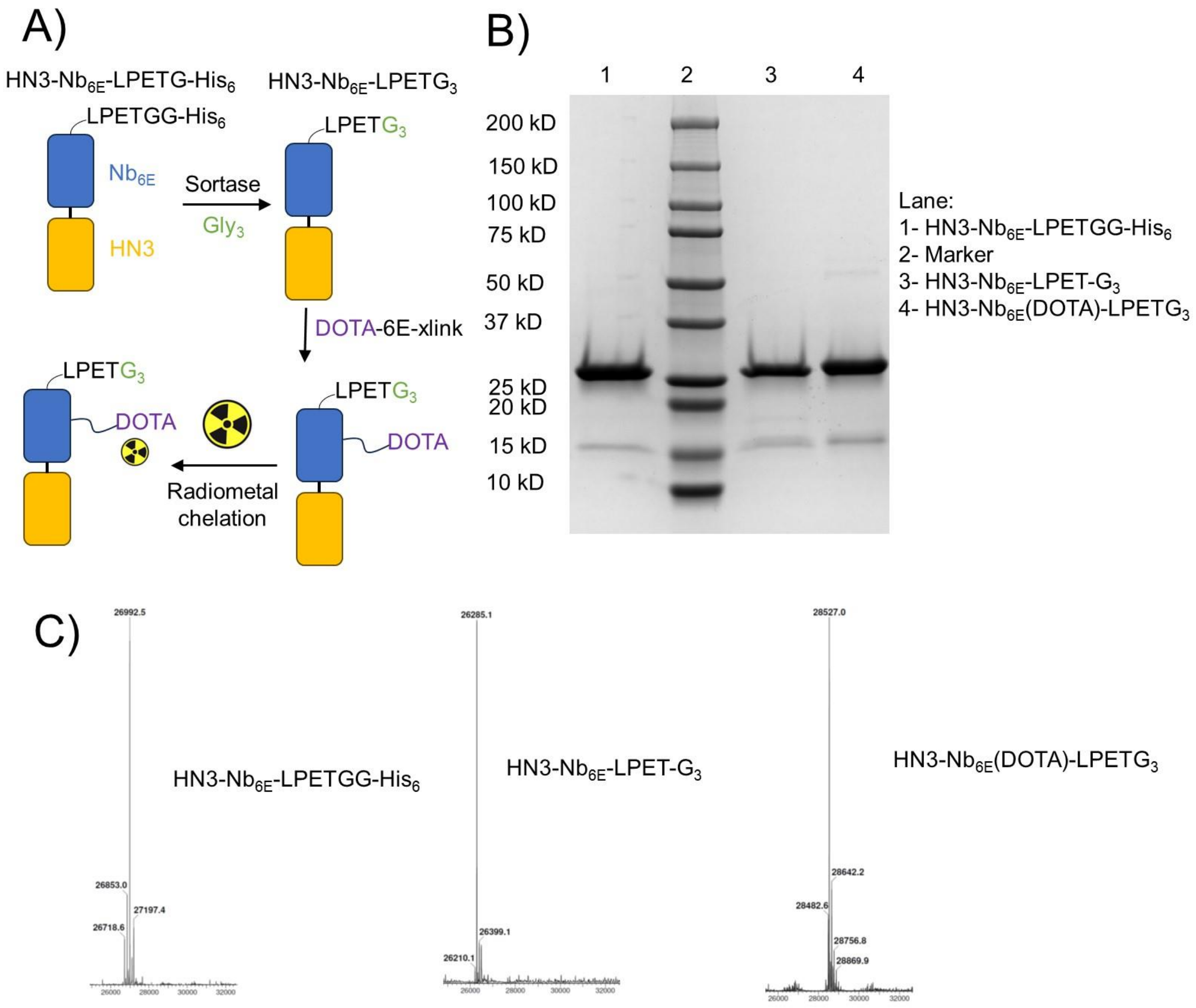

Supporting Figure S8: Assessment of labelling of HN3-Nb$_{6E}$ with DOTA-6E-xlink for use in imaging experiments. A) Schematic of labeling chemistry used for preparation of constructs used for in vivo experiments. HN3-Nb$_{6E}$-LPETGG-His$_6$ was used as generated by a commercial manufacturer (GenScript, TurboCHO production). HN3-Nb$_{6E}$-LPET-G$_3$ was produced through a Sortase A mediated labeling reaction, as described in Methods. HN3-Nb$_{6E}$(DOTA)-LPET-G$_3$ was produced by exposure of HN3-Nb$_{6E}$-LPET-G$_3$ (20 µM) to DOTA-6E-xlink (25 µM) for 2 h at 4°C. **B**) Crude reactions were analyzed by SDS-PAGE and **C)** mass spectrometry as described in Methods. An uncropped image of the SDS-PAGE gel (panel b) and mass spectra (panel c) are shown below. Note that panel b, lane 1 is reproduced in Figure 2b in the main text. The final product (HN3-Nb$_{6E}$(DOTA)-LPET-G$_3$) was purified by size exclusion chromatography (HiLoad 16/600 Superdex 75 pg) in ammonium acetate (50 mM, pH 5.5) buffer. The typical overall yield for the two-step reaction prior to radiometal chelation was 50%. Material purified using this method was used for *in vivo* application.

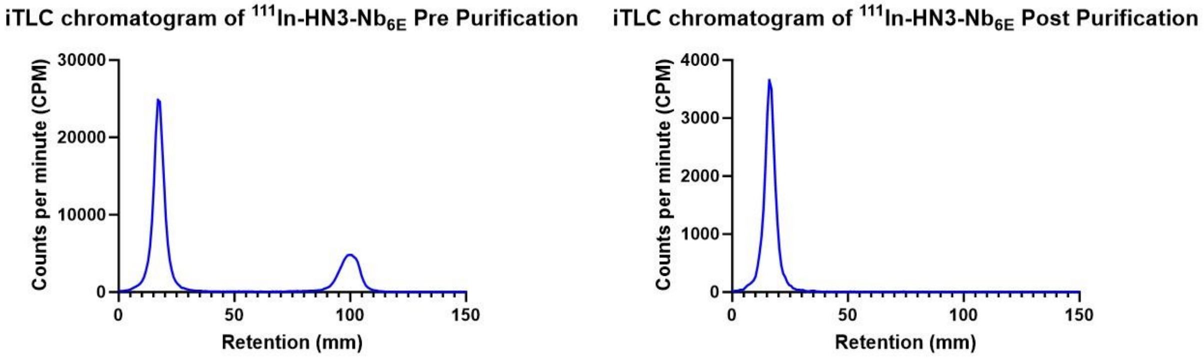

Supporting Figure S9: Characterization of radiometal (^{111}In) complexation with HN3-Nb_{6E}(DOTA)-LPET-G_3 using instant thin layer chromatography (iTLC). Analysis of the chelation reaction between Nb_{6E}(DOTA)-LPET-G_3 and ^{111}In was performed using iTLC either prior to (left) or after (right) purification of the reaction using a miniature size exclusion column (PD10 desalting column, Cytiva #17085101). The radioactivity peak with low mobility corresponds to the Nb_{6E}(DOTA)-LPET-G_3 - ^{111}In complex. See Methods for experimental details.

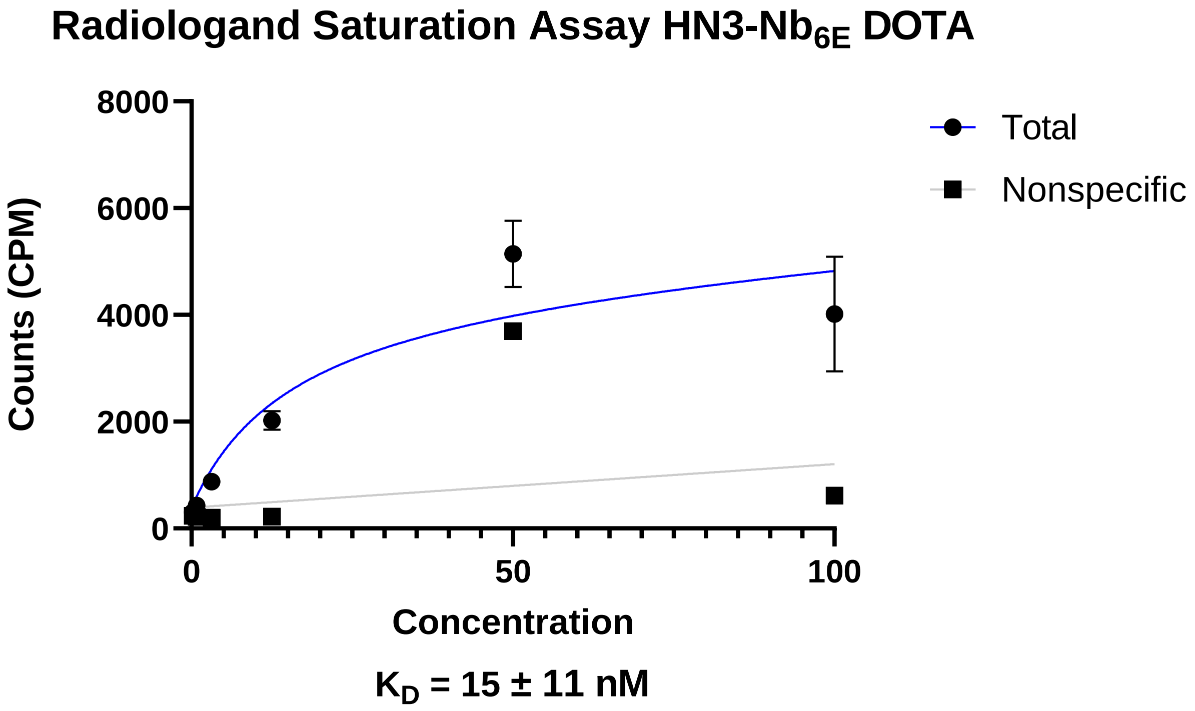

Supporting Figure S10: Binding of HN3-Nb_{6E}(DOTA)-^{111}In to GPC3. Recombinant GPC3 was immobilized onto high binding multiwell plates prior to addition to radiolabeled HN3-Nb_{6E}. Bound radioactivity was measured by gamma counting. Data corresponds to mean ± SD from technical replicate measurements. See Supporting Methods for details.

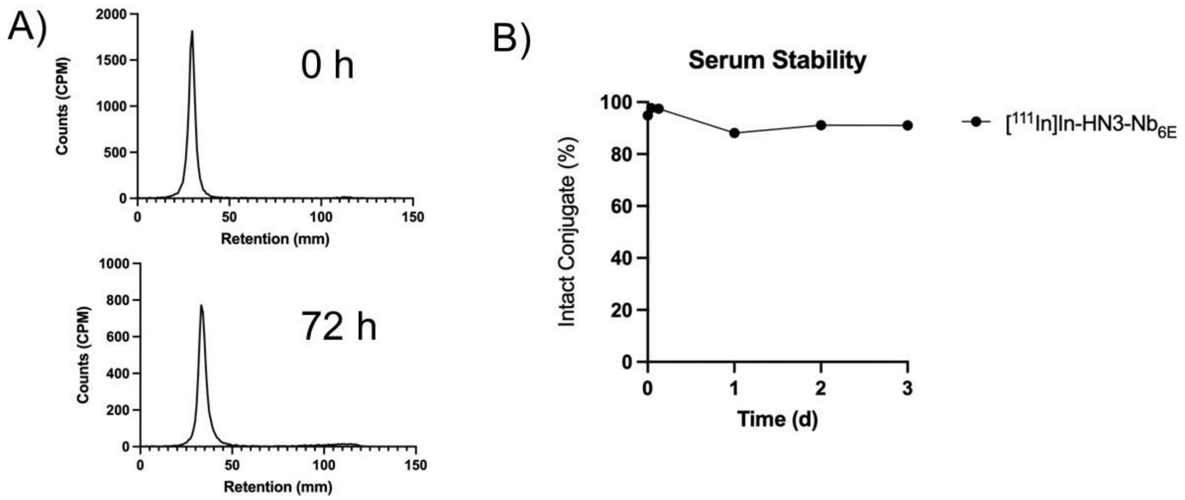

Supporting Figure S11: Demonstration of the stability radiolabeled nanobody conjugates in human serum. Radiolabeled conjugates [^{111}In]In-HN3-Nb$_{6E}$ was incubated with reconstituted human serum at 37° C for the indicated durations prior to analysis by iTLC (described in Methods). A) iTLC analyses of sample composition at indicated time points. The signal with low mobility (low retention distance) corresponds to intact [^{111}In]In-HN3-Nb$_{6E.}$ B) Time course analysis conjugate stability.

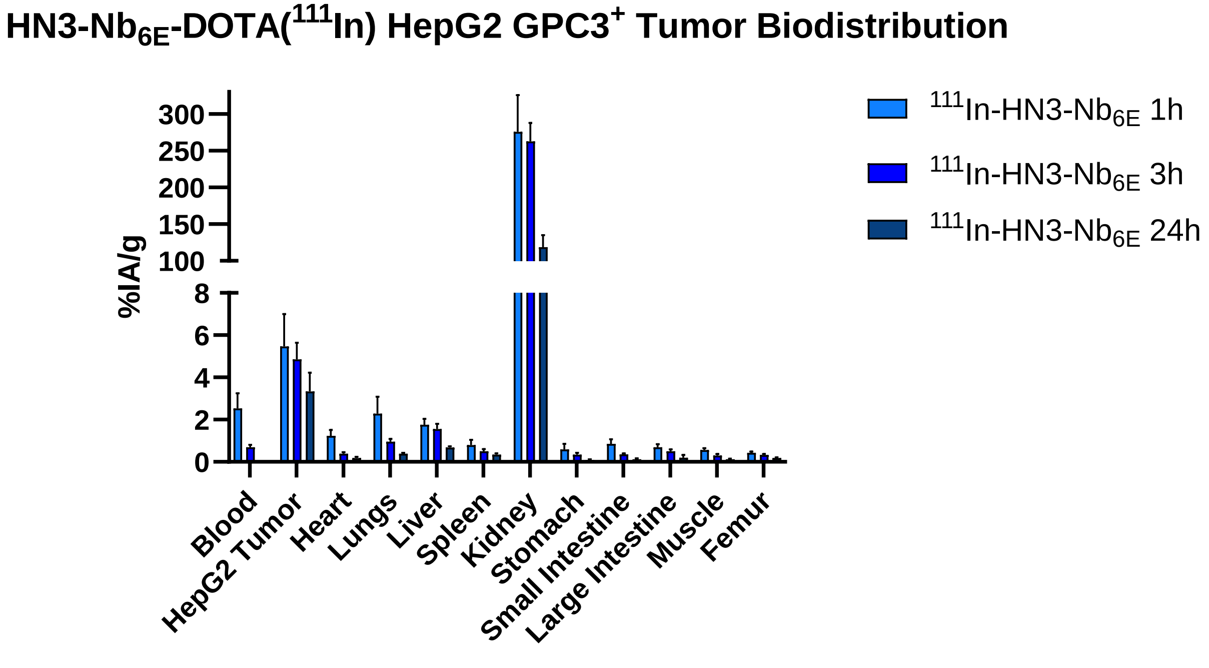

Supporting Figure S12: Biodistribution of HN3-Nb$_{6E}$-DOTA(^{111}In)- in HepG2 tumors expressing GPC3. Data correspond to mean ± SD from measurements in three separate mice. Experiments were performed as described in Methods.

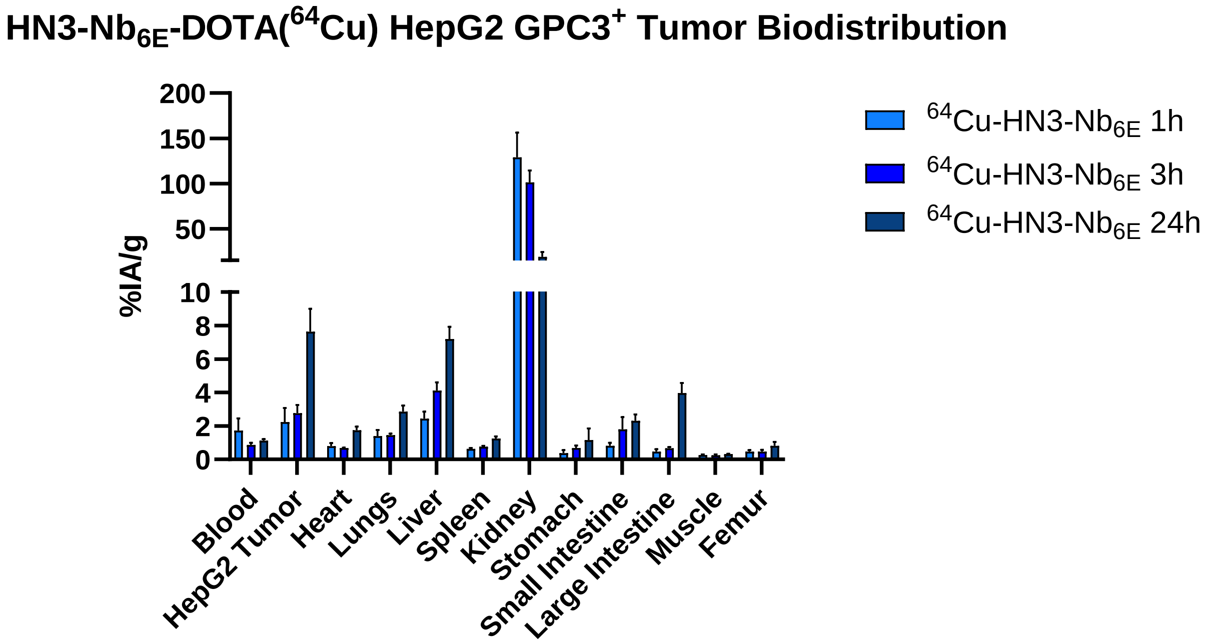

Supporting Figure S13: Biodistribution of HN3-Nb6E-DOTA(^{64}Cu)- in HepG2 tumors expressing GPC3. Data correspond to mean ± SD from measurements three separate mice. Experiments were performed as described in Methods.

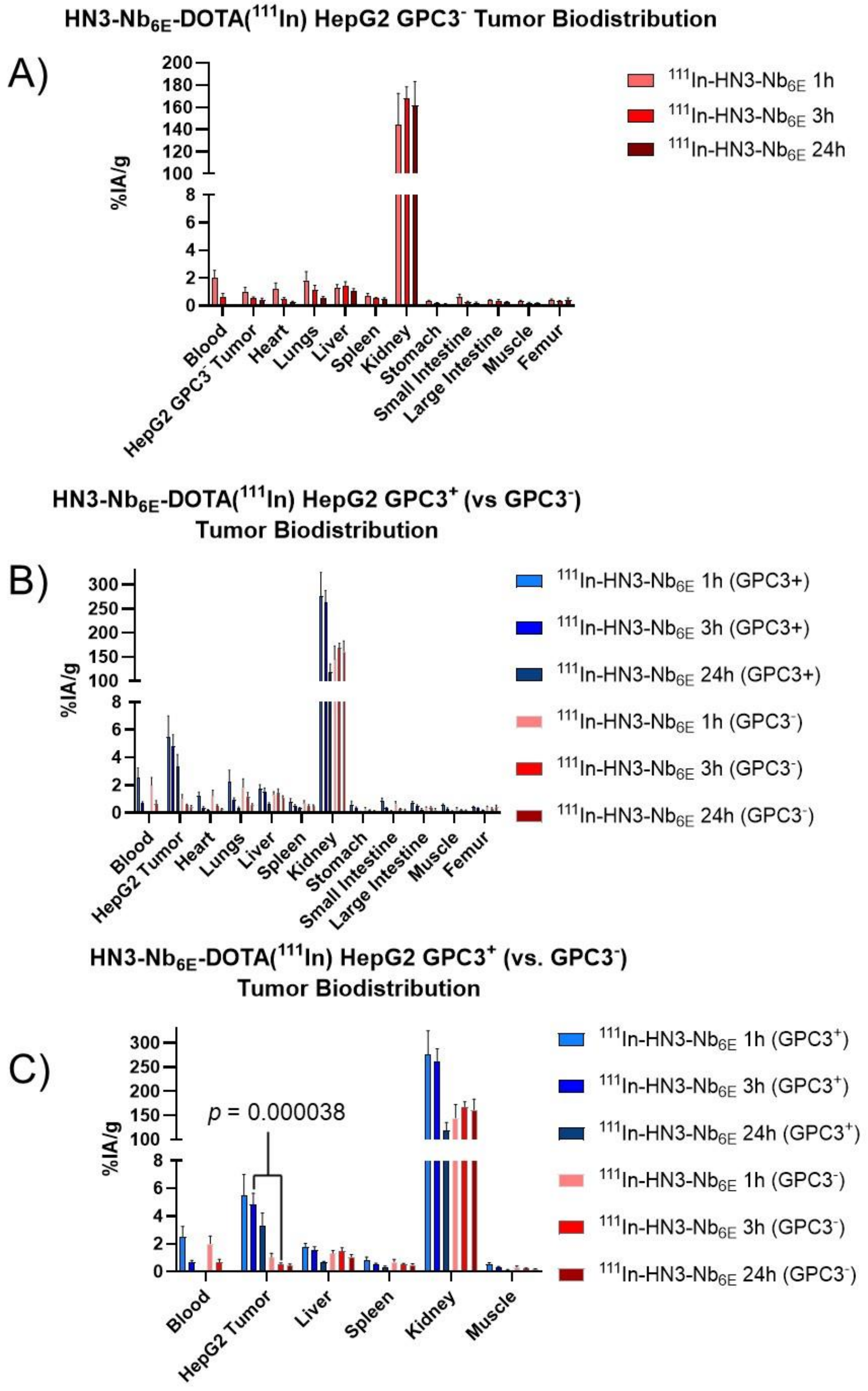

Supporting Figure S14: Biodistribution of HN3-Nb6E-DOTA(^{111}In)- in HepG2 tumors lacking GPC3. A) Time course biodistribution data for HepG2 tumors lacking GPC3. Data correspond to mean ± SD from measurements four separate mice. Experiments were performed as described in Methods. **B**) Overlay of data from Supporting Figure 12 and panel A. **C**) Plot of a subset of data from panel b. Students *t* test (2 tailed, unpaired, parametric) was used to assess statistical differences.

Supporting Methods:

Peptide Synthesis, Cleavage, and Purification. All peptides were synthesized via Fmoc solid phase peptide synthesis on a Gyros PurePep Chorus Automated Peptide Synthesizer or Liberty Blue 2.0 Microwave Peptide Synthesizer. Protected amino acids were purchased from ChemImpex or ChemPep. Peptide assembly was performed on Rink Amide resin (0.05 mmol scale) to afford a C-terminal carboxamide. For syntheses on the Gyros PurePep, Fmoc-amino acids (8 equivalents) were dissolved in dimethylformamide (DMF) and added to resin () with PyAOP (7-Azabenzotriazol-1-yloxy)trispyrrolidinophosphonium hexafluorophosphate) and N,N-diisopropylethylamine (DIPEA, 16 equivalents). Fmoc groups were deprotected using 20% piperidine in DMF. For syntheses on the Liberty Blue, Fmoc amino acids were dissolved in DMF and added to resin (5 equivalents) with diisopropylcarbodiimide (10 equivalents) and Oxyma Pure (5 equivalents, Ethyl cyano(hydroxyimino)acetate). Coupling steps were performed at 90°C for 2 m. Fmoc groups were deprotected using 20% piperidine in DMF at 90°C for 1 m.

Cleavage of peptides was performed using a cleavage cocktail comprised of trifluoroacetic acid(TFA)/H_2O/triisopropylsilane(TIS) (92.5:5:2.5% by volume). Product was precipitated using chilled diethyl ether and pelleted by centrifugation (3,000 RPM for 2 minutes). Diethyl ether was decanted, and the pellet was dried under N_2 prior to being dissolved in DMSO and purified by HPLC. Peptides were purified via preparative-scale HPLC using a Phenomenex Aeris Peptide XB-C18 Prep column (particle size 5 µM, 100 Å pore size) with a linear gradient of solvent A (0.1% TFA in H_2O) and solvent B (0.1% TFA in acetonitrile). Fractions of interest were combined and lyophilized. Lyophilized peptides are then dissolved in DMSO at desired concentrations and frozen.

Analogues of 6E-xlink modified at the N-terminus with moieties of interest were synthesized by conjugation of appropriately functionalized building blocks to the N-terminus of peptide on resin, with exceptions. Commercially available building blocks were used for the attachment of FAM [5(6)-carboxyfluorescein, AnaSpec], Biotin [N-α-Fmoc-N-ε-Biotinyl-L-lysine, ChemPep], DOTA [DOTA-tris (t-Bu ester), Macrocyclics], Azide [Nα-Fmoc-Nε-azide-L-Lysine, ChemImpex], and Halo [7-Bromoheptanoic Acid, Cayman chemical]. 10 equivalents of these building blocks were dissolved in DMF, activated with HATU (1-[Bis(dimethylamino)methylene]-1H-1,2,3-triazolo[4,5-b]pyridinium 3-oxid hexafluorophosphate, 10 equivalents), and DIPEA (20 equivalents). Coupling reactions were carried out over night at room temperature, followed by washing and exposure to standard peptide cleavage conditions (described above).

C16 was attached to the peptide *N*-terminus through a reaction of resin-bound peptide (exposed amino terminus) with palmitoyl chloride (Sigma Aldrich). Peptide was treated with a mixture of palmitoyl chloride/diisopropylethylamine/dichloromethane (1:2:8, v/v) for 20 minutes at room temperature. The resin was washed with dichloromethane and then treated with piperidine in DMF (20% v/v) to degrade and residual palmitoyl chloride. Peptide was then cleaved from resin and purified as described above.

Desferroxamine (DFO) was attached to the peptide N-terminus through a reaction of p-SCN-Bn-Deferoxamine (Macrocyclics, #B-705) with purified H_2N-6E-C5 peptide. p-SCN-Bn-Deferoxamine was dissolved in DMF at a stock concentration of 10 mM. p-SCN-Bn-Deferoxamine (5 equivalents) was then added to a H_2N-6E-C5 (1 equivalent) with DIPEA (3 equivalents). The reaction was mixed at room temperature overnight then purified by HPLC.

AZDye555-6E-xlink

To a solution of Azide-6E-xlink (7 mM) in DMSO was added DBCO-AF555 (Vector #CCT-1290) at a final concentration of 15 mM. The reaction was mixed at room temperature for 3 h and then purified by reverse phase HPLC. Product identity was confirmed by mass spectrometry.

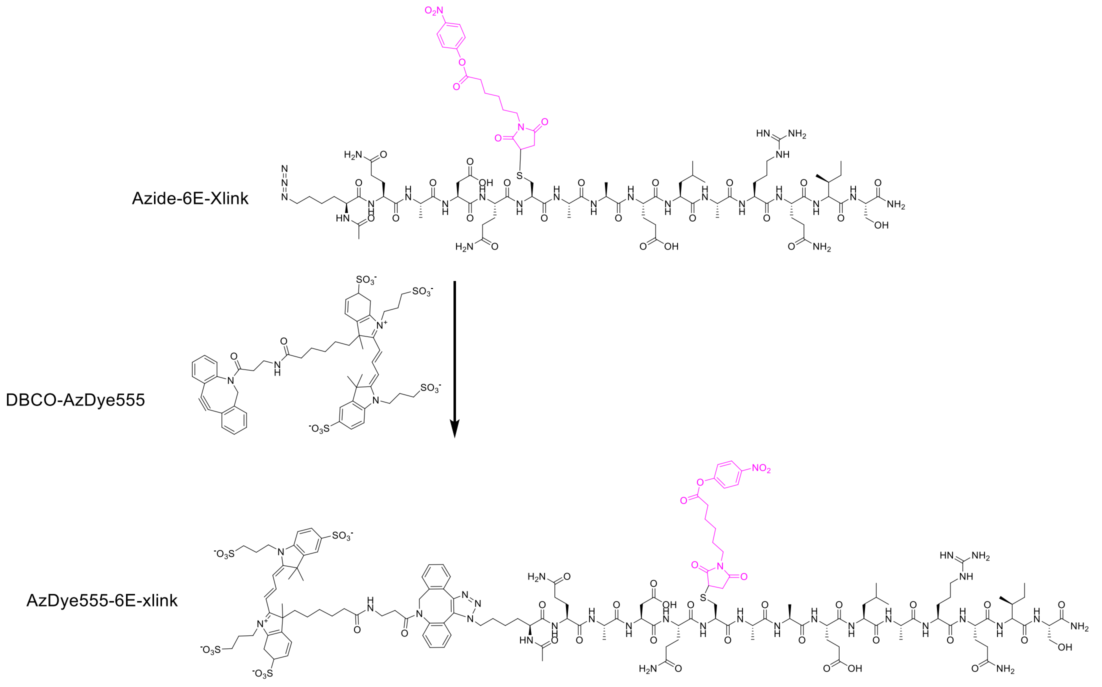

Azide-6E-xlink (representative example)

Synthesis of crosslinking peptides was carried out as previously described.[1] Azide-6E-C5 was dissolved in DMSO at a concentration of 2 mM. To this solution was added maleimide-phenol-NO_2 at a final concentration of 8 mM and 1 M pH 7.4 phosphate buffer at a final concentration of 50 mM. The reaction was mixed at room temperature for 2 h followed by purification on reverse phase HPLC. Product identity was confirmed via mass spectrometry (see **Supporting Table 1**).

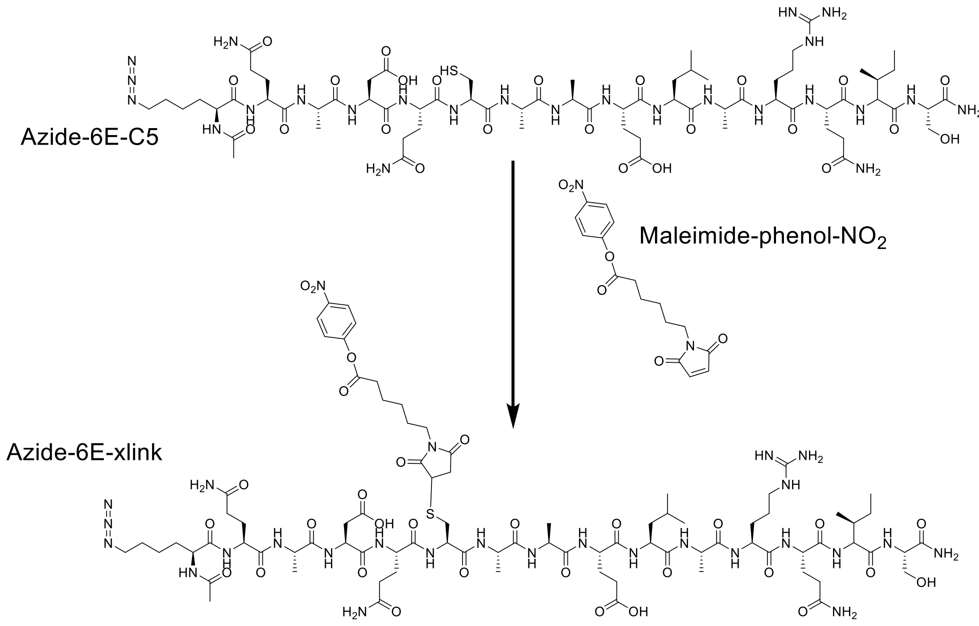

G_3-FAM

To a solution of G_3-Cys (purchased from GenScript, custom synthesis) at 5 mM in DMSO was added FAM-maleimide (AnaSpec, AS-81405) at a final concentration of 15 mM and 1 M pH 7.4 phosphate buffer at a final concentration of 50 mM. The reaction was shaken at room temperature for 2 h and purified by reverse phase HPLC.

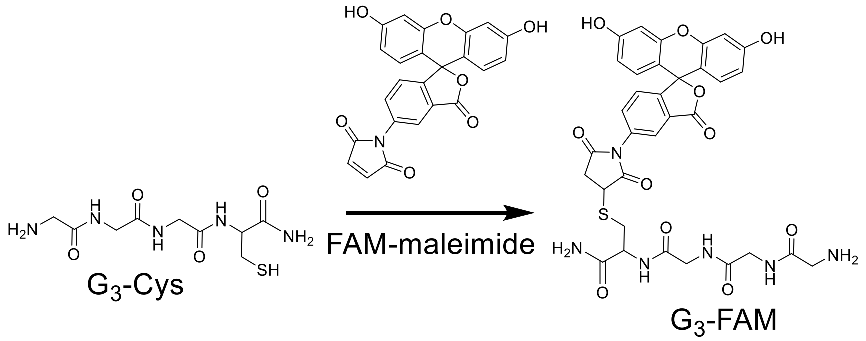

Nanobody purification from *E. coli.* BL21(DE3) *E. coli* were transfected via heat shock with pET26b(+) plasmids encoding nanobodies of interest (HN3-Nb_{6E}, Nb_{Kappa}-Nb_{6E}) and grown in medium (Terrific Broth) containing kanamycin (50 µg/mL). Transformed bacteria were used to generate a starter culture, which was used to inoculate full-size cultures (1-4 L) containing kanamycin. This culture was grown at 37°C with growth monitored through measurement of the optical density at 600 nm (OD_{600}). When OD_{600} values between 0.3 and 0.8 were observed, protein expression was induced by addition of Isopropyl β-d-1-thiogalactopyranoside (IPTG, 1 mM). The induced culture was then shaken 16°C for 2 days.

Bacteria were harvested via centrifugation for 30 min at 6,000 RPM (Avanti J Series centrifuge). Cells were resuspended in 30 mL of NTA wash buffer (tris buffered saline + 10 mM imidazole, pH 7.5) containing protease inhibitor (Pierce Protease Inhibitor Tablets, ThermoFisher A32953).

Cells were then lysed via sonication and the lysate was centrifuged at 15,000 RPM for 45 min. The supernatant was then passed through a fritted column containing nickel NTA beads (His Pur™ Ni-NTA Resin) equilibrated with Nickel NTA wash buffer. After initial flowthrough, beads were washed 3x with Nickel NTA wash buffer. Subsequently, bound protein of interest was eluted using 10 mL of Nickel NTA elution buffer (TBS + 150 mM imidazole, pH 7.5). Sample was subjected to size exclusion chromatography (Cytiva Akta / Pure) using a HiLoad 16/600 Superdex 200 pg column with an isocratic flow of TBS (Flow rate 1 mL/min). Fractions of interest were collected and concentrated via centrifugation using spin filtration columns (Amicon Ultra-15, regenerated cellulose, 10,000 nominal molecular weight limit). Protein concentrations were determined by measuring absorption at 280 nm measured on a Nanodrop spectrophotometer.

Monomeric HN3 human single-domain antibody was produced by the Antibody Engineering Program at NCI Center for Cancer Research following a published protocol.[2]

Flow cytometry (A20): A20 cells were transferred to a round bottom 96 well plate, pelleted by centrifugation (500 rpm for 3 min.), and resuspended in PBS containing 2% BSA (w/v) (PBS/BSA), and pelleted a second time. The washed cell pellets were then resuspended in PBS/BSA with varying concentrations of fluorescein-labeled Nb constructs for 30 minutes on ice. Cells were then washed with PBS/BSA, centrifuged, and resuspended in PBS/BSA containing Alexafluor647 conjugated anti-fluorescein antibody (1:1000 dilution in PBS/BSA) and incubated for 30 min on ice prior to washing. Washed cells were then resuspended in PBS/BSA for analysis by flow cytometry on a CytoFlex flow cytometer (Beckman Coulter). Live cells were identified based on forward scatter/side scatter profile and staining intensity was monitored in the APC channel. A minimum of 2,000 events corresponding to live cells were recorded.

Flow cytometry (A431). A431 and A431-GPC3+ (G1 cells) were lifted using Trypsin 0.25% EDTA and centrifuged for 5 min at 1700 X g, 4 °C. The supernatant was discarded, and the cell pellets were resuspended in DMEM media. Cells were then aliquoted into experimental and control vials at a concentration of 500,000 cells per tube. Control vials consisted of unstained and fluorescently stained samples, while experimental vials included HN3-Nb_{6E} variants and native HN3. Vials were spun down and washed once with ice-cold FACS buffer (1% bovine serum albumin in 1X phosphate buffered saline). Experimental tubes were then incubated for 45 minutes with a 1 µM concentration of his-tagged nanobodies on ice in the dark. Samples were then washed with FACS buffer, and designated groups were stained with 6x-His Tag Monoclonal Antibody, Alexa Fluor 488 (Thermo Fisher) for 30 min on ice in the dark. Data was collected using a Beckman Coulter Cytoflex cytometer running CytExpert Software (v2.4), and results were analyzed with FlowJo (v10.10.0).

Liquid chromatography/mass spectrometry (LC/MS) analysis of peptides and proteins. Mass spectrometry data was acquired on a Waters Xevo qTOF LC/MS or an Agilent Affinity II 6130 quadrupole LC/MS instrument. Samples were analyzed in positive ion mode. For large proteins analyzed by mass spectrometry singly charged ions were not observed, so protein intact mass was calculated from analysis of multiply charged ions using the MaxENT algorithm on MassLynx software. For smaller peptides, the neutral mass (M) was determined from most abundant isotopic peak (p) and its charge (z) using the equation $M = (p*z) - z$. This value was compared to the calculated M (isotopic) of the compound, derived from molecular formula.

Protein labeling via sortagging. Sortagging reactions were comprised of the following components: Protein bearing a sortase recognition motif (LPETGG) followed by a hexa histidine

tag at the C-terminus (20-200 µM final concentration), triglycine-probe conjugates (500-2000 µM final concentration), and Sortase 5M or Sortase 7M (10-20 µM final concentration). Triglycine probes were either purchased commercially (Gly_3, ChemImpex #04555), synthesized using Fmoc-based solid phase peptide synthesis (G_3-azide, peptide synthesis methods described above), or produced using cysteine-maleimide chemistry (G3-FAM, see above). Reactions were performed sortase buffer (10 mM $CaCl_2$, 50 mM Tris, 150 mM NaCl, pH 7.5 for Sortase 5M) or PBS (for Sortase 7M) and shaken overnight at 10°C overnight. After incubation, the reaction was incubated with nickel NTA beads to capture Sortase 5M and unreacted starting protein. Uncaptured material was further purified using disposable desalting columns to remove triglycine conjugates (Cytiva PD-10 Sephadex™ G-25M). Fractions containing product were combined then concentrated by spin filtration (Amicon Ultra 0.5 mL Centrifugal Filters 10,000 NMWL).

Radioligand Saturation Binding Assay. The binding affinity of the radiolabeled nanobody was determined via a radioligand saturation assay using methods previously described.[3] Briefly, immobilized recombinant GPC3 was incubated with varying concentrations (0.2-100 nM) of radiolabeled HN3-Nb_{6E} for 2 hours on a 96-well high binding plate (Corning), and samples were run on a gamma counter (Perkin Elmer) to determine total and specific binding.

Evaluation of radiolabeled HN3-Nb_{6E} stability in serum. [^{111}In]In-HN3-Nb_{6E} was incubated in human serum (Millipore). Aliquots of radiolabeled nanobody (880 kBq) were incubated in 1 mL plasma on a thermomixer (Eppendorf) at 37 °C, pH 7.4. At discrete time points, a sample was removed and analyzed by iTLC (see main **Methods**).

Mass spectral characterization of peptides and conjugates.

FAM-6E-C5

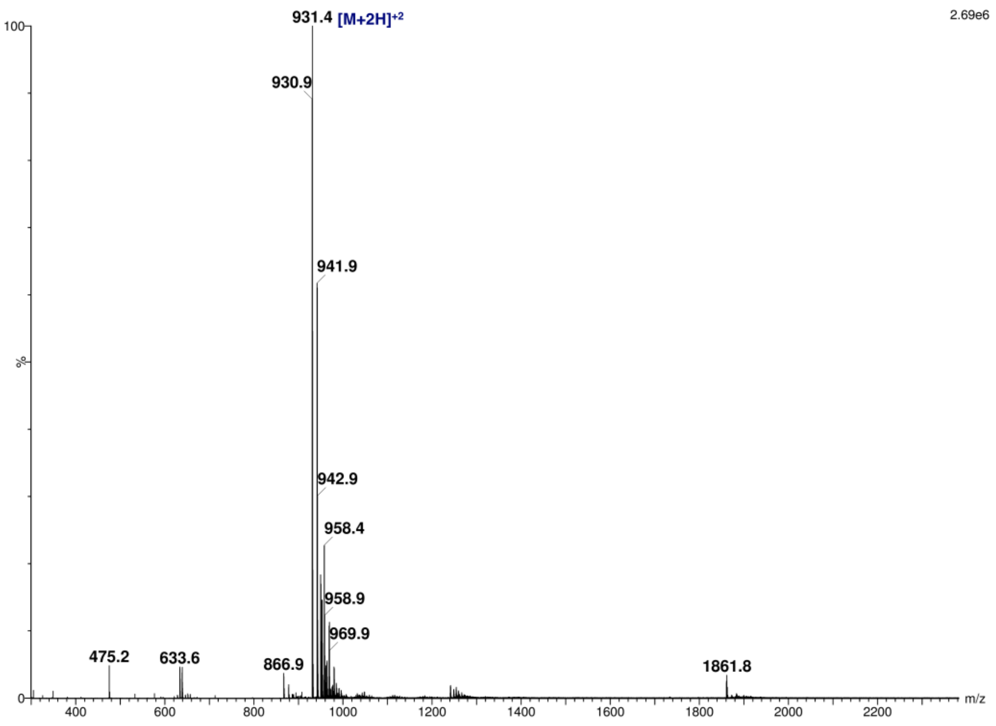

FAM-6E-C5-xlink

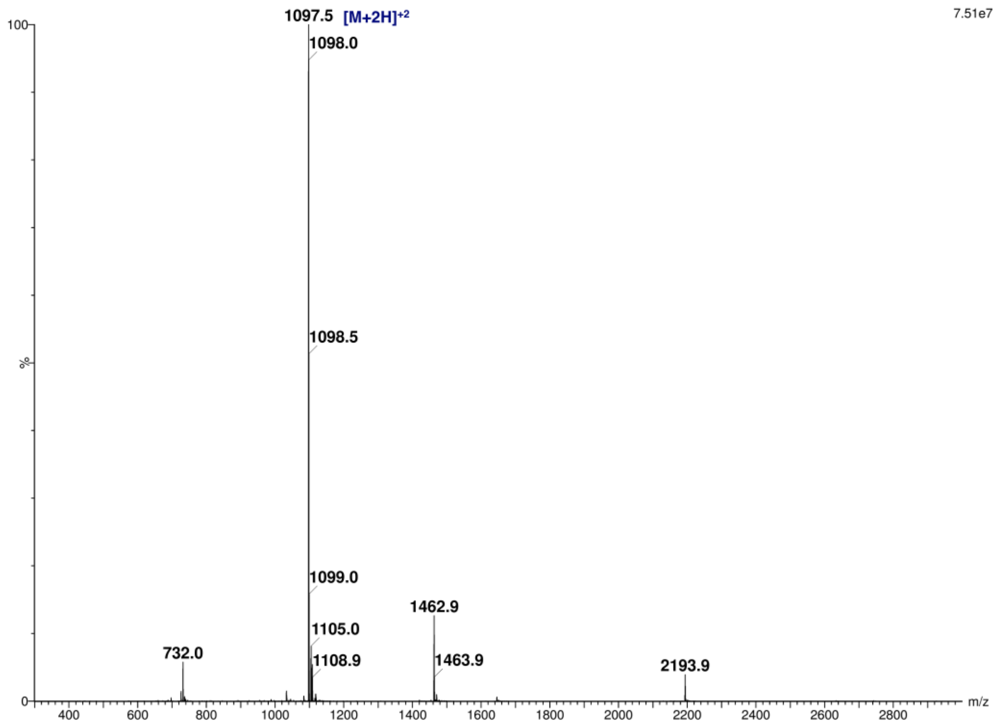

Biotin-6E-C5

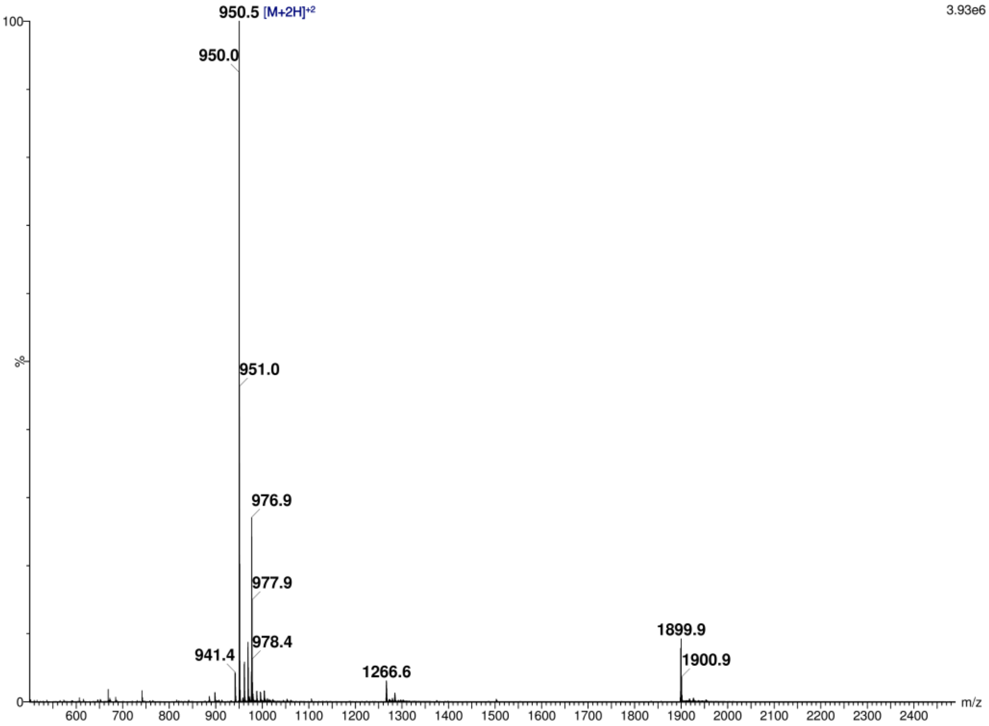

Biotin-6E-xlink

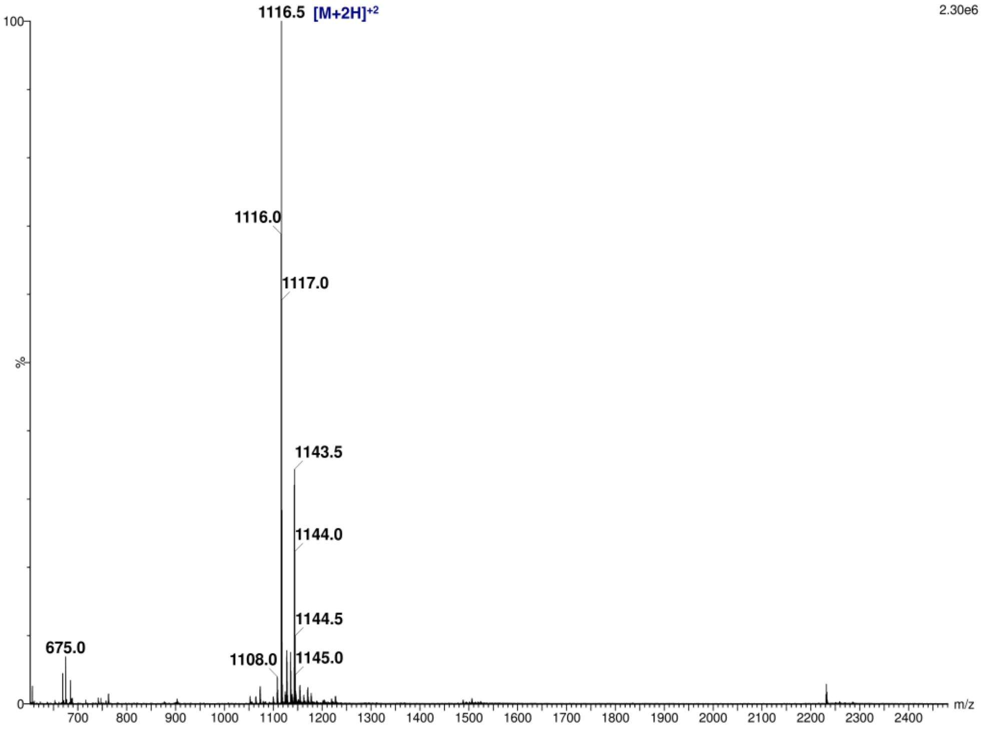

DOTA-Ahx-6E-C5

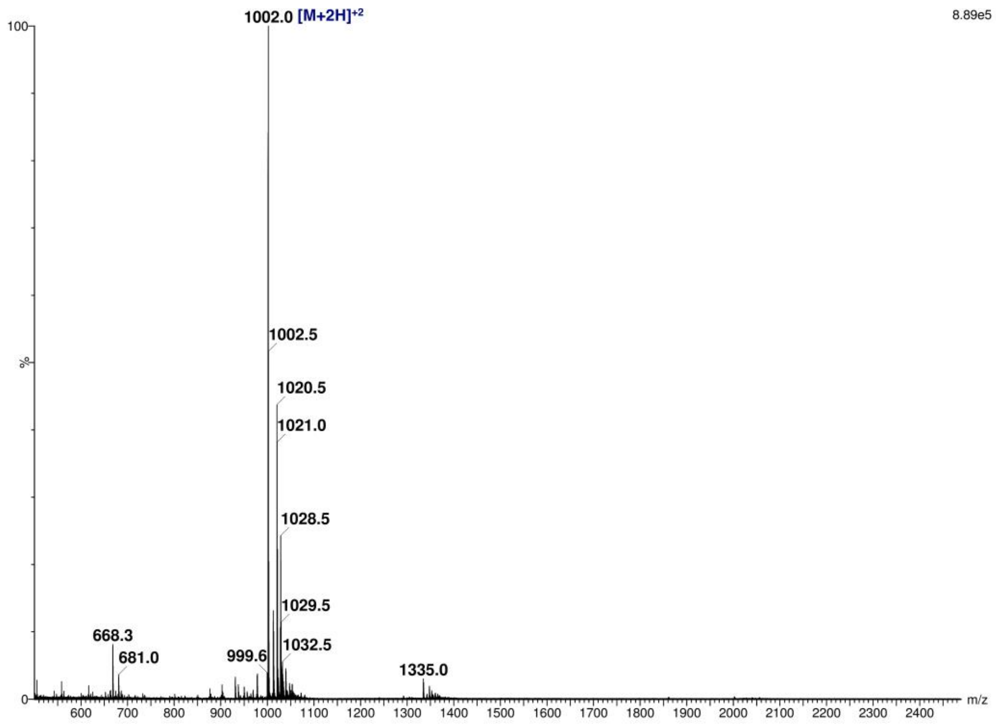

DOTA-Ahx-6E-xlink

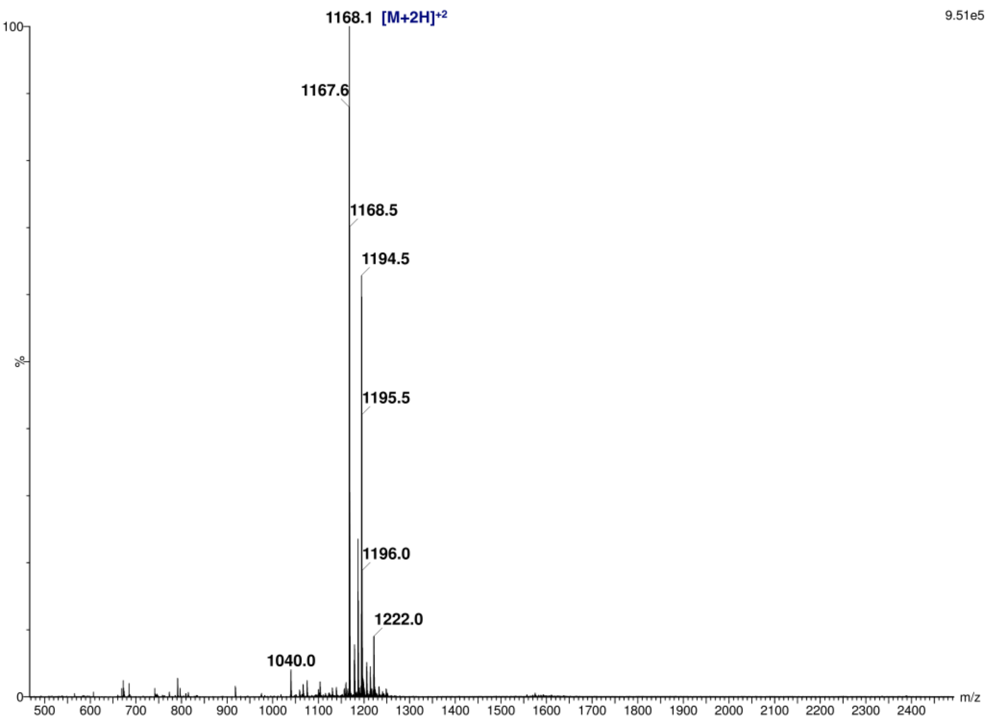

C16-6E-C5

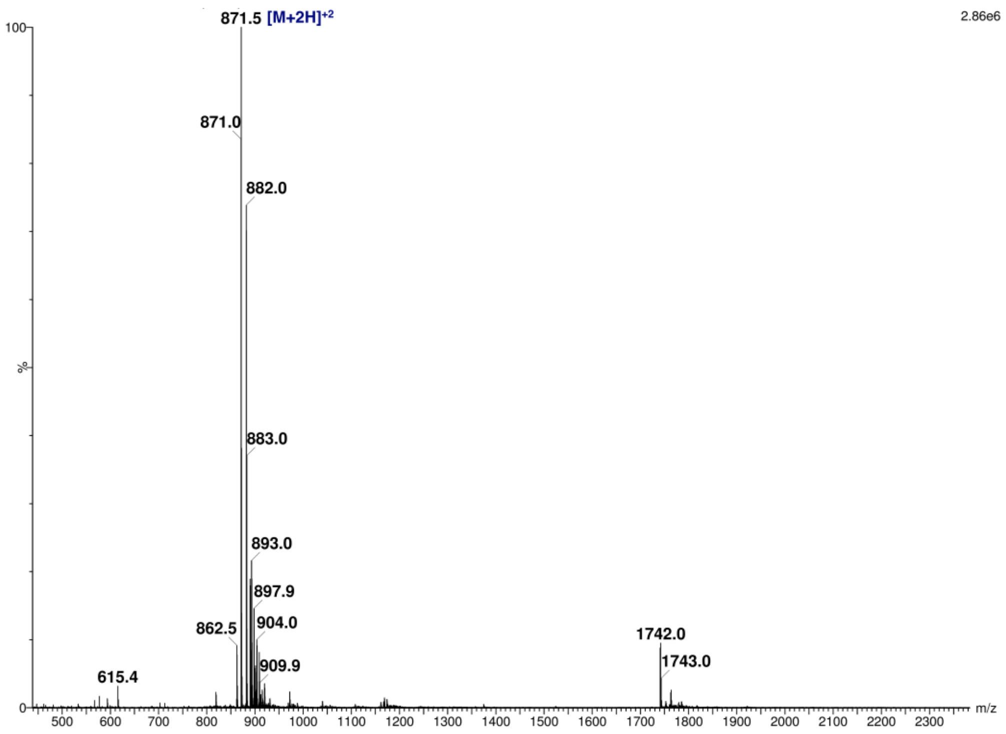

C16-6E-C5-xlink

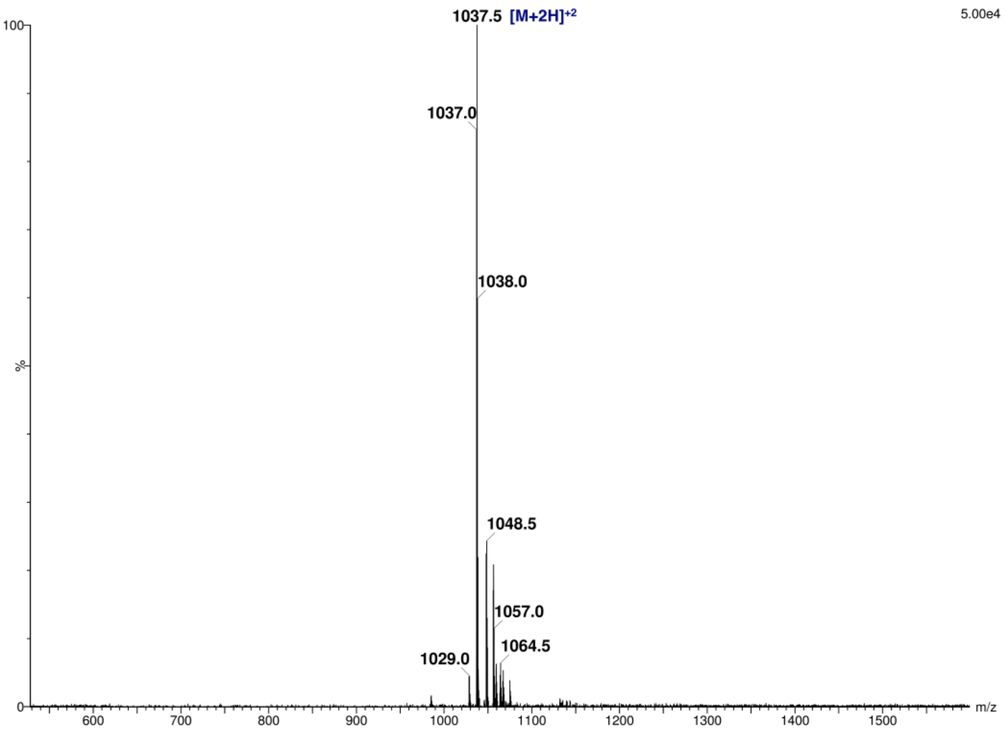

DFO-6E-C5

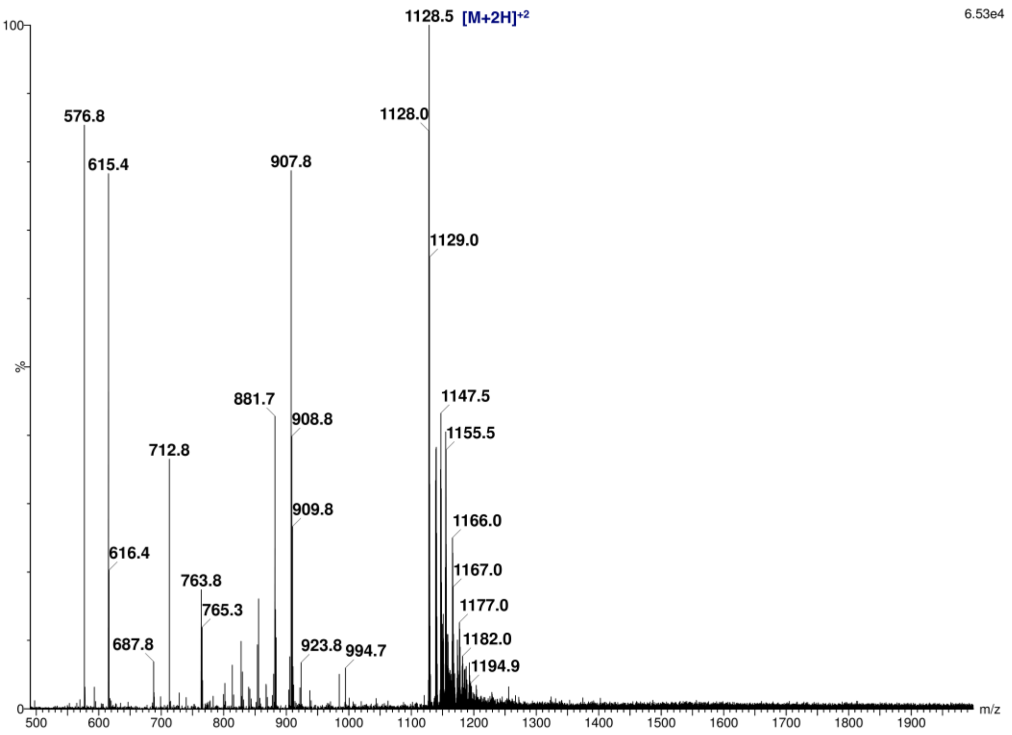

DFO-6E-xlink

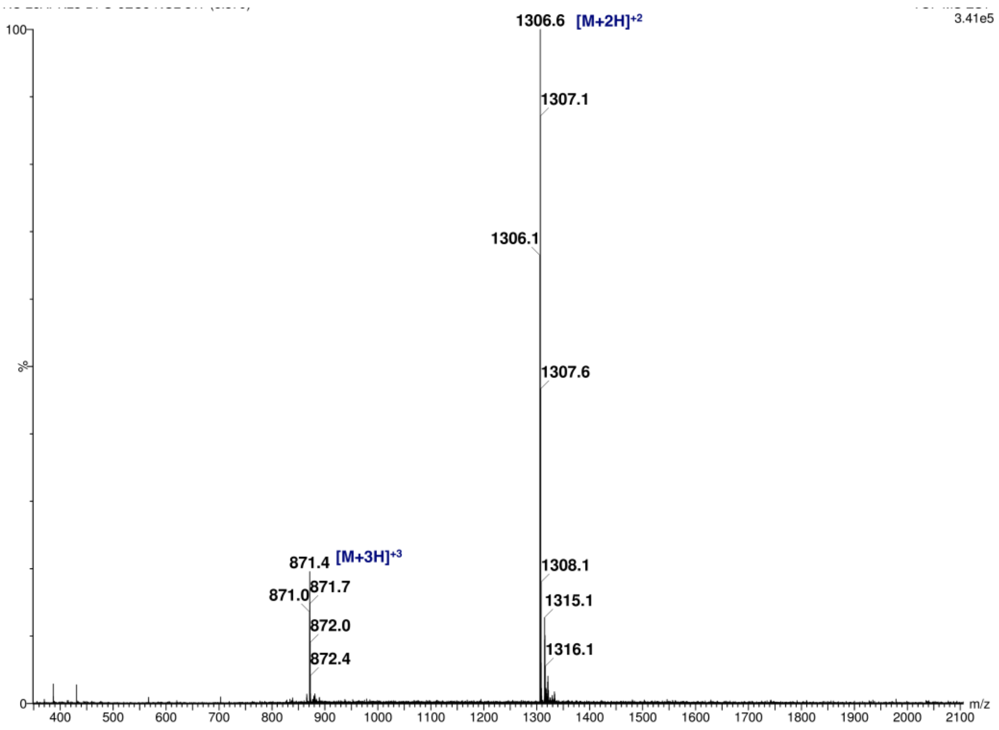

Azide-6E-C5

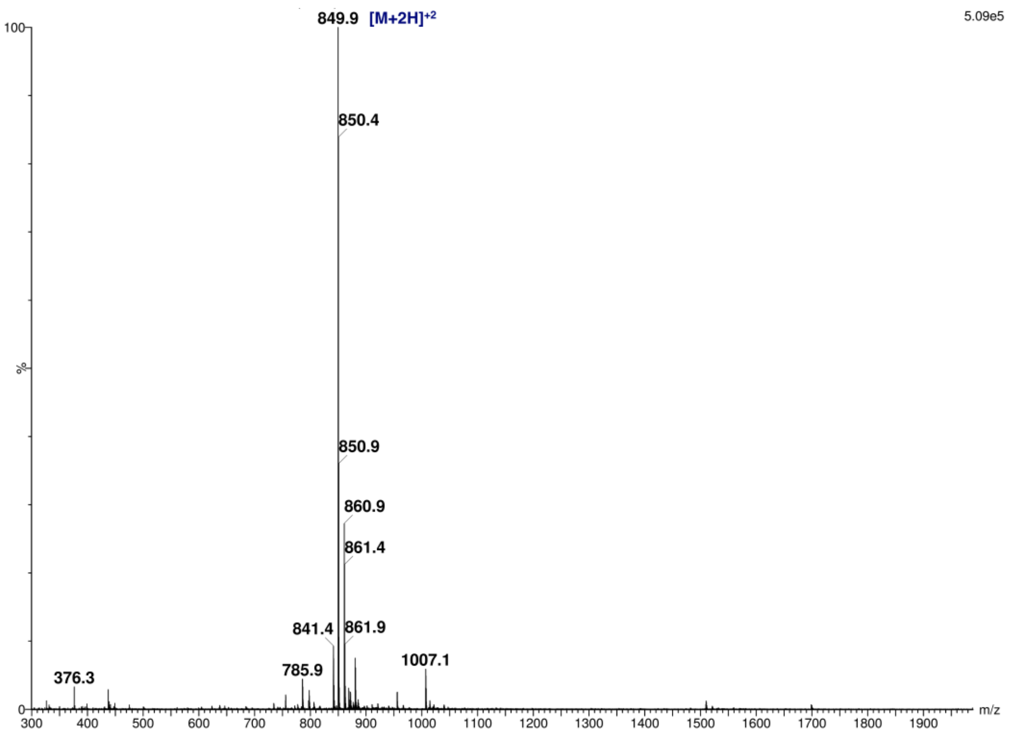

Azide-6E-xlink

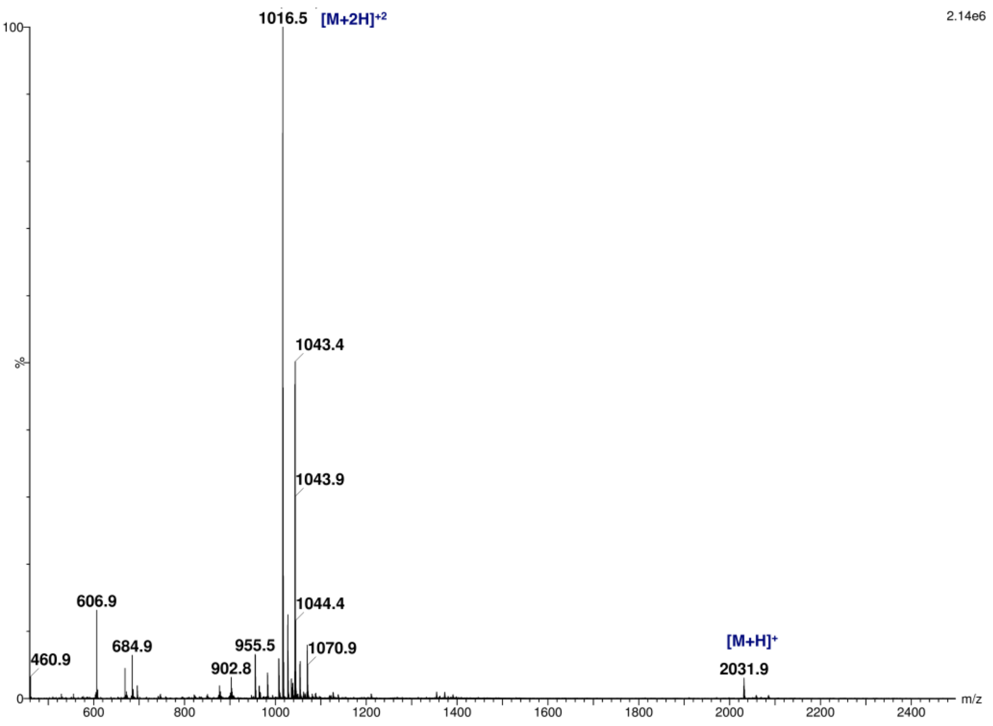

Halo-Ahx-6E-C5

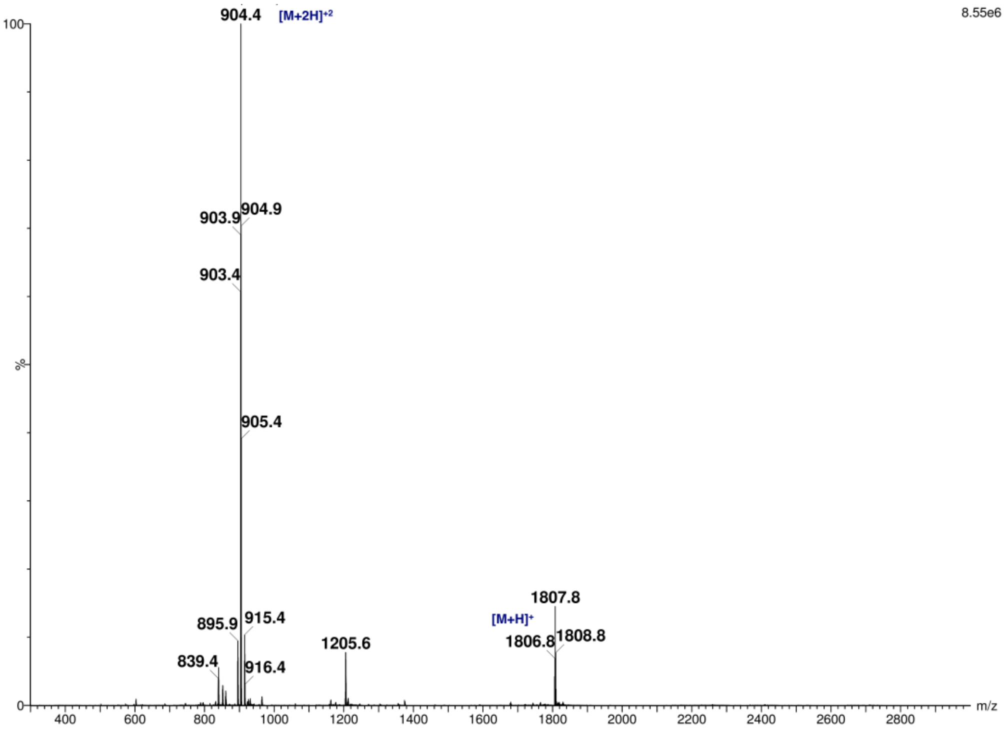

Halo-Ahx-6E-xlink

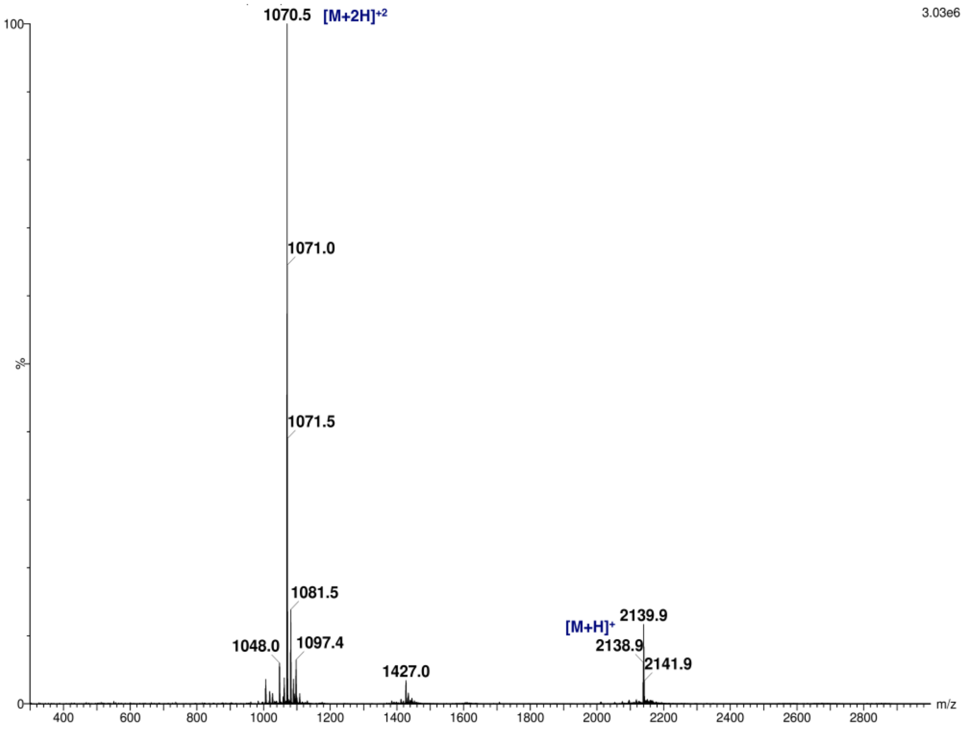

AZ-dye555-6E-xlink

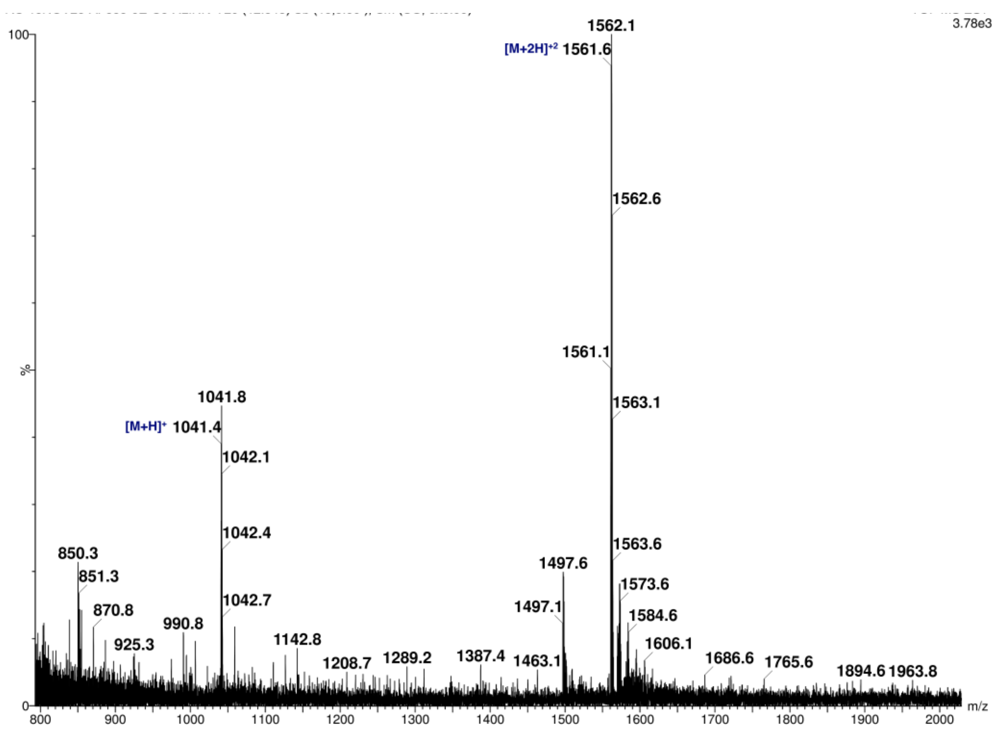

Purity checks of the crosslinking peptides:

All purity check were performed using an Aeris 5µm PEPTIDE XB-C18 100 Å LC column (250 x 4.6 mm) with a water-acetonitrile solvent system containing 0.1% TFA added. Purity checks were performed with a gradient of 20-80% acetonitrile in water over 25 m.

FAM-6E-C5-xlink

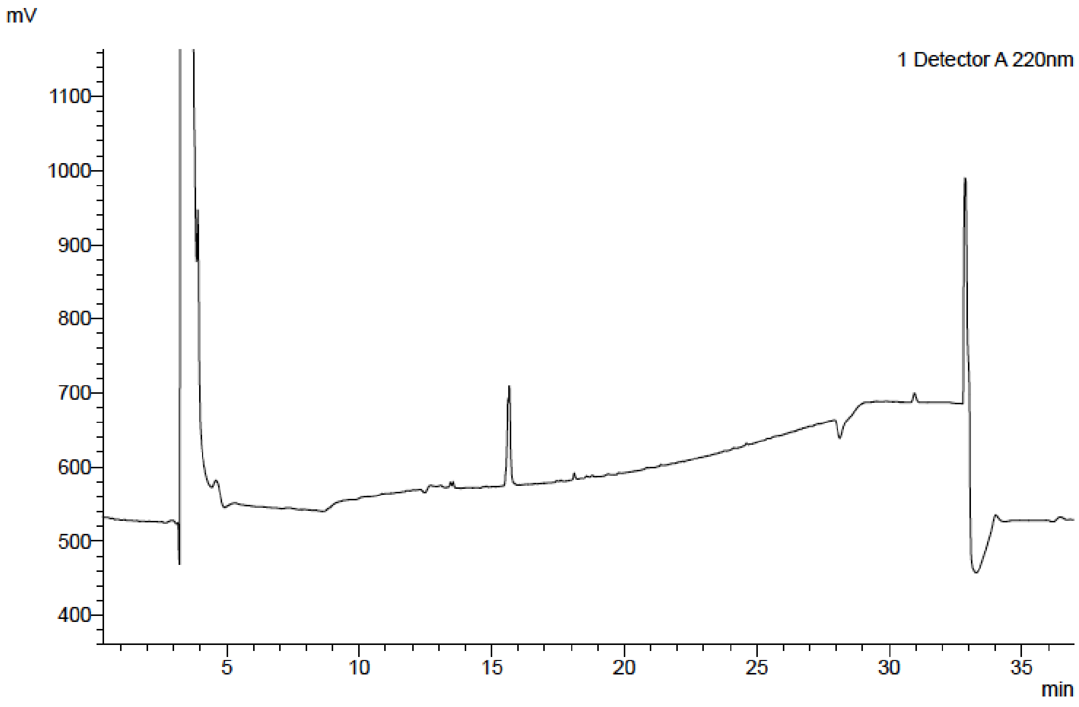

Bio-6E-C5-xlink

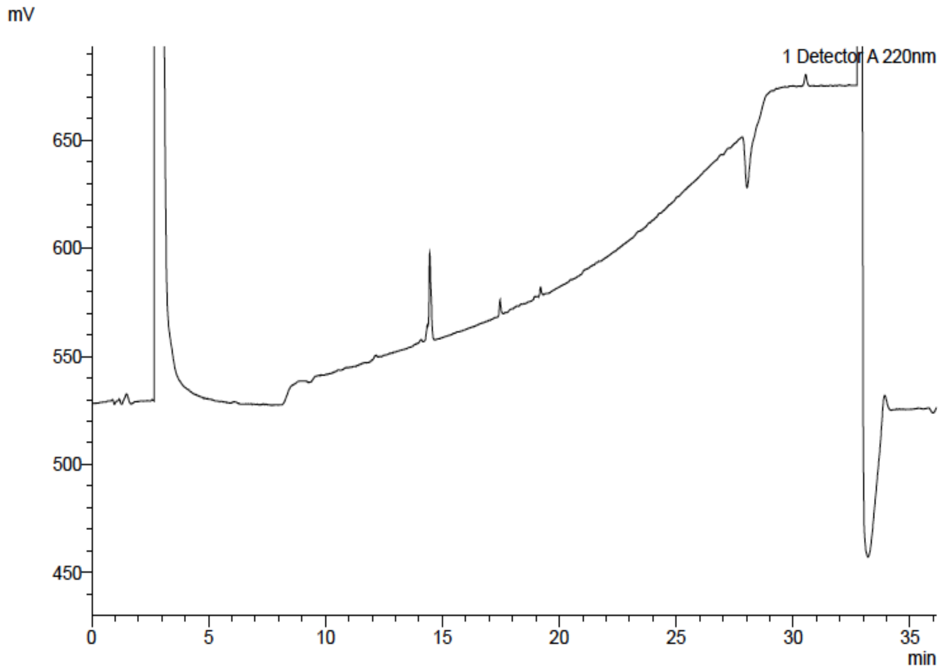

DOTA-Ahx-6E-C5-xlink

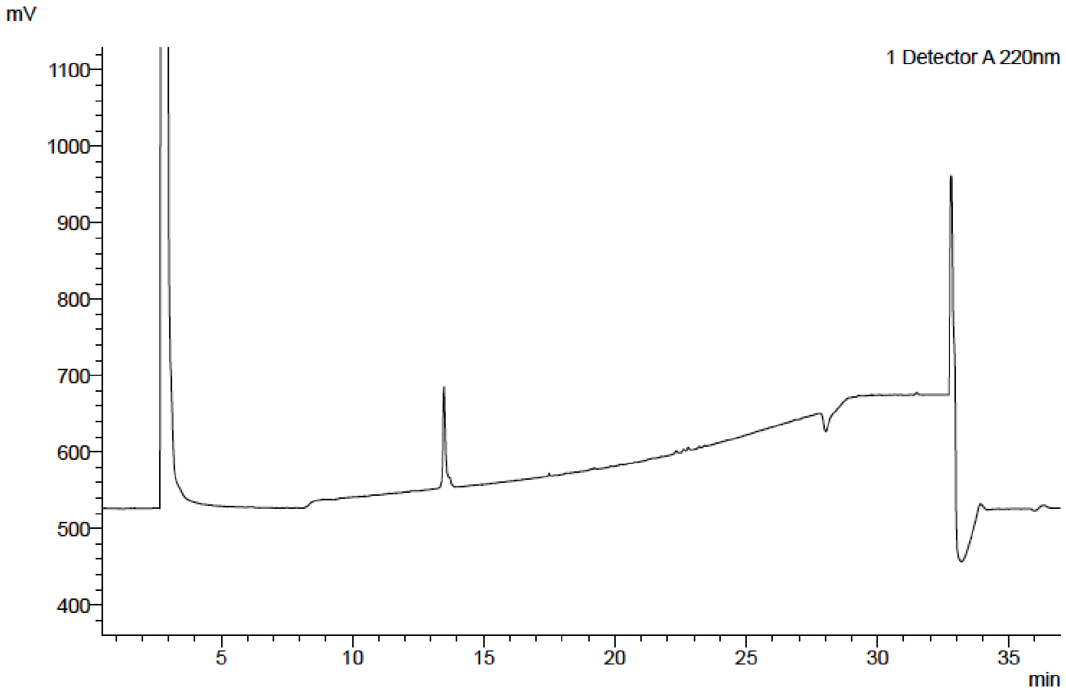

C16-6E-C5-xlink

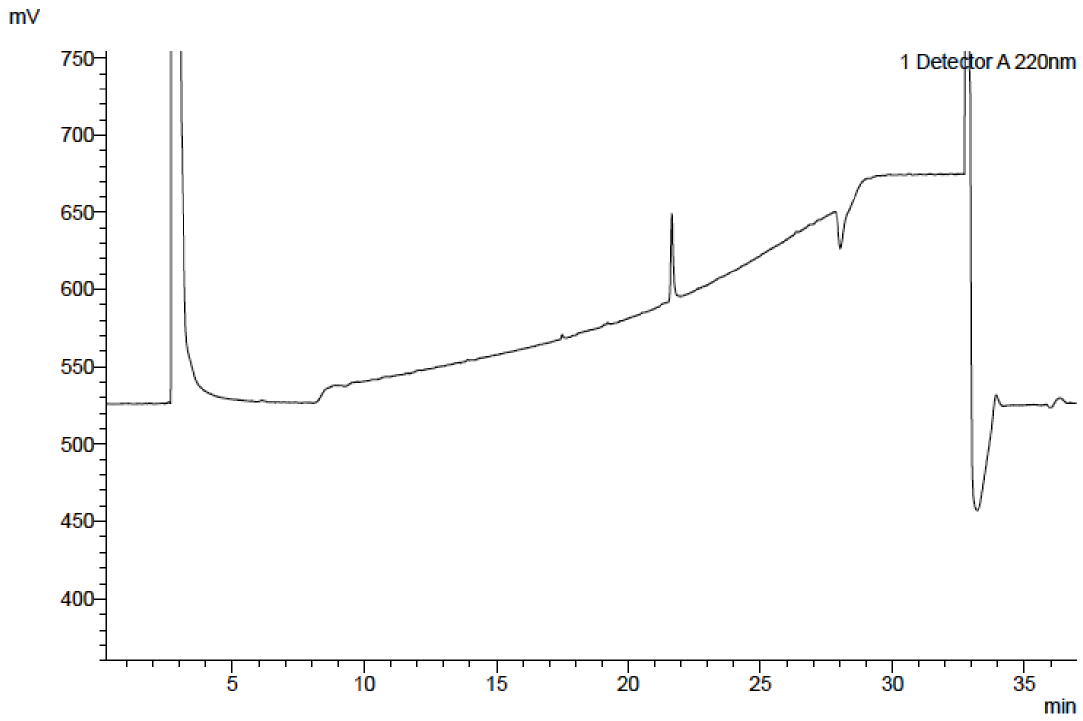

DFO-6E-C5-xlink

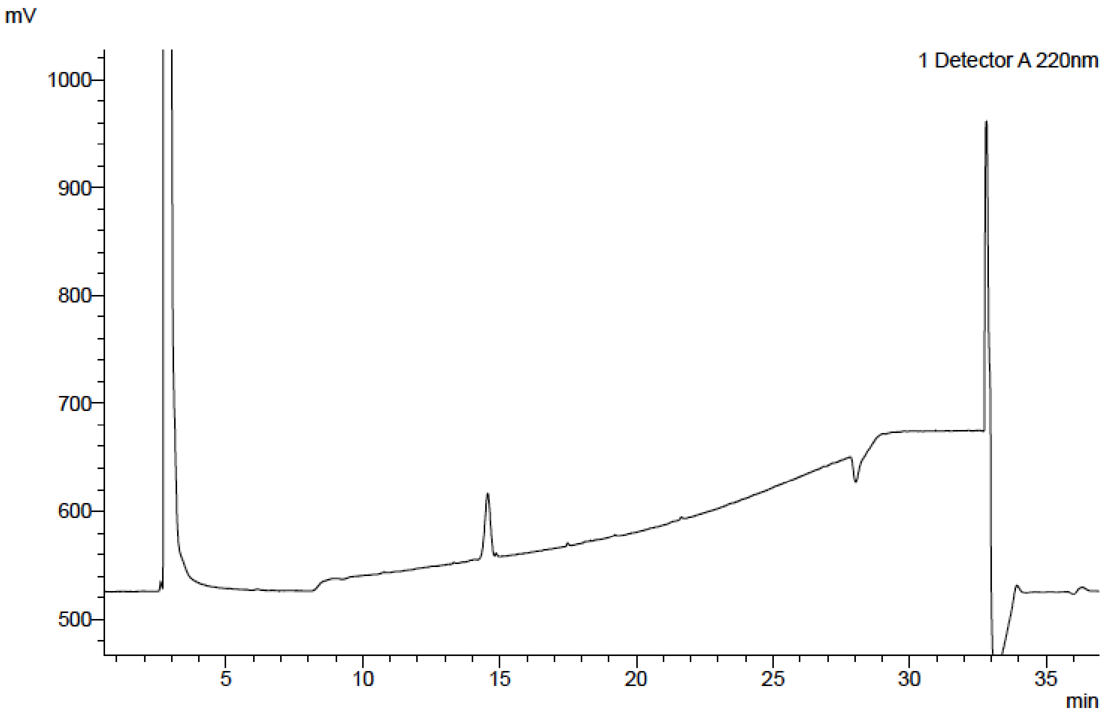

Azide-6E-C5-xlink

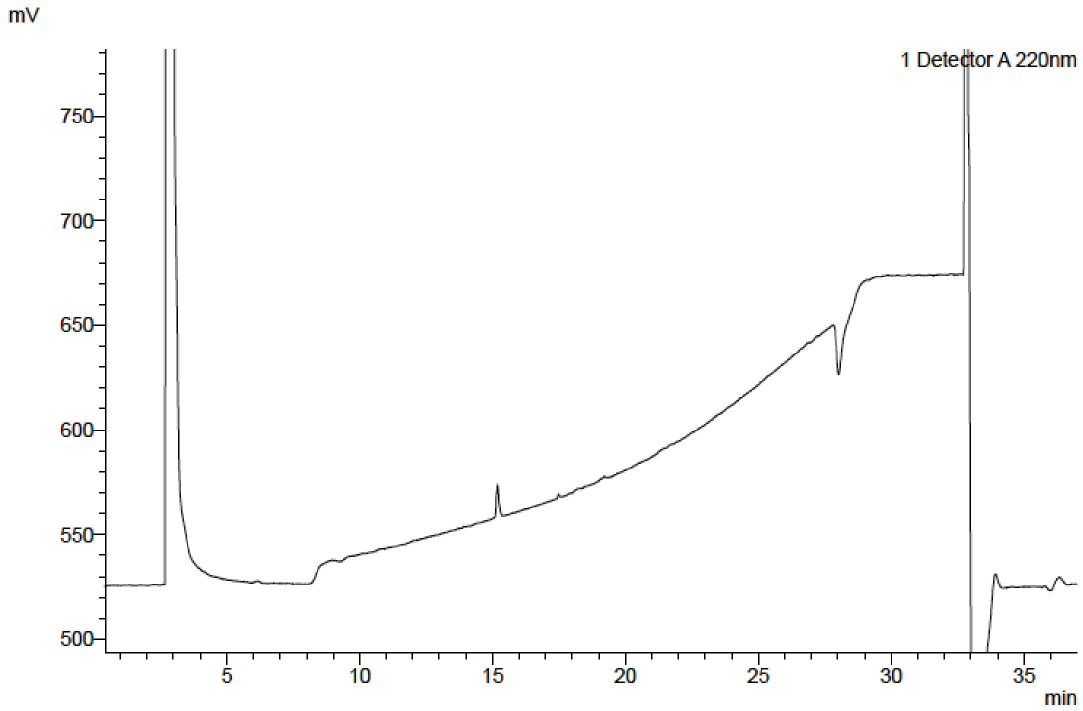

Halo-6E-C5-xlink

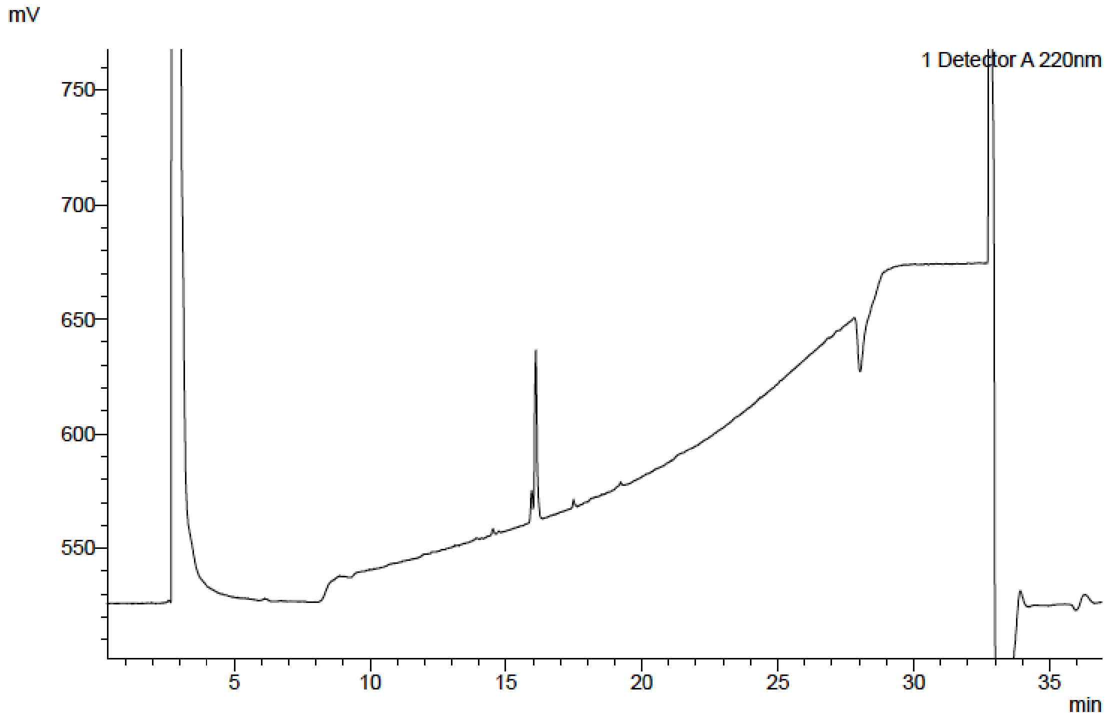

AZDye555-6E-C5-xlink

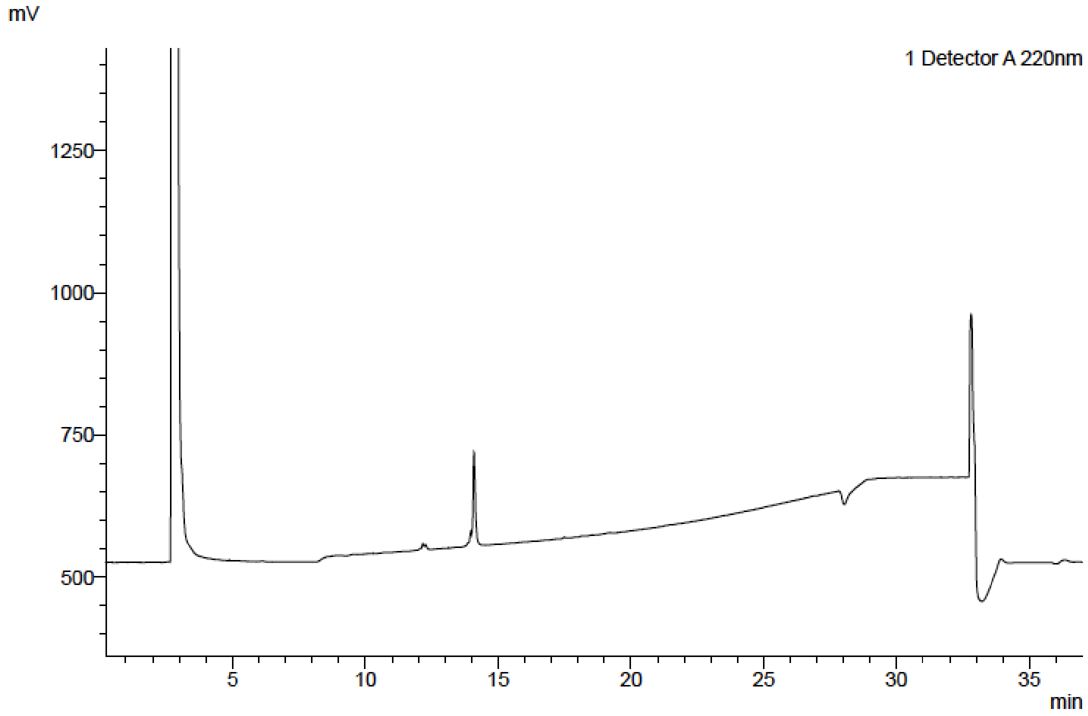

Mass spectrometry characterization of nanobodies and nanobody conjugates

Nb_{kappa}-Nb_{6E}

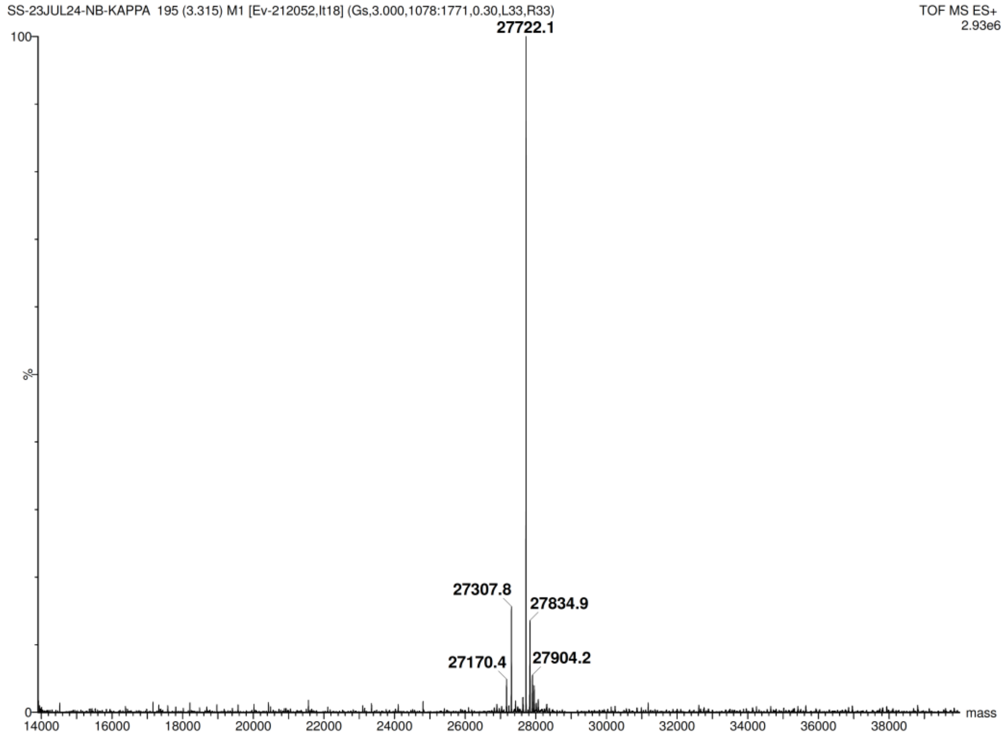

Nb_{Kappa}-Nb_{6E}-FAM-xlink

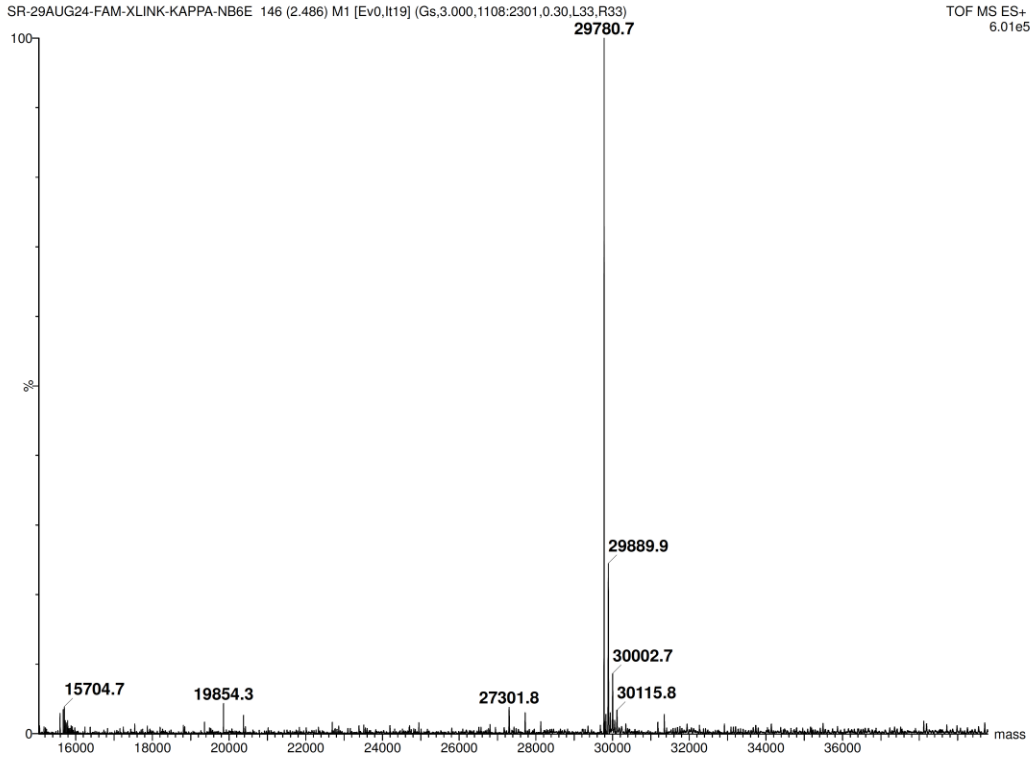

Nb_{Kappa}-Nb_{6E}-G_3-FAM

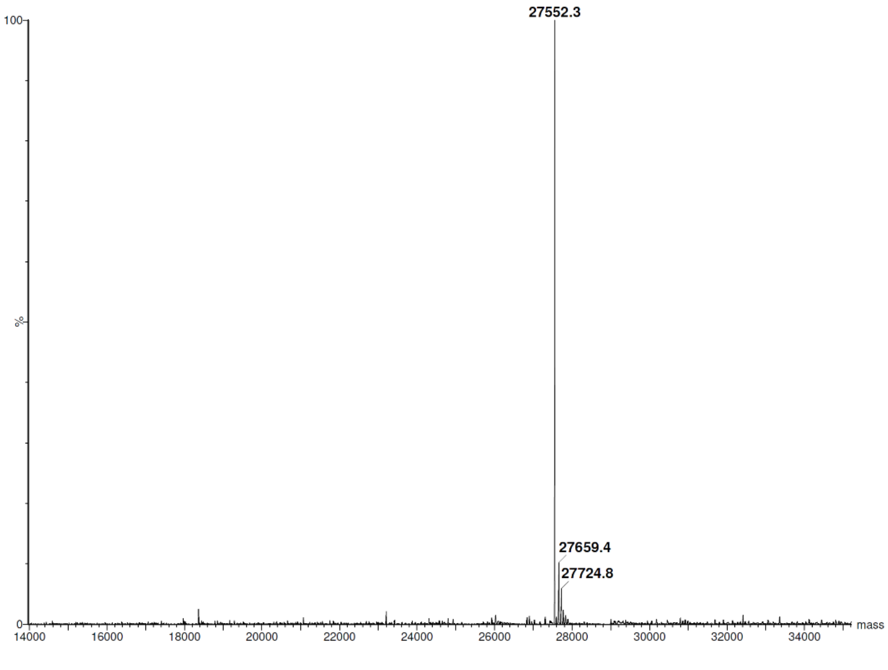

Nb$_{kappa}$-Nb$_{6E}$-DOTA-xlink

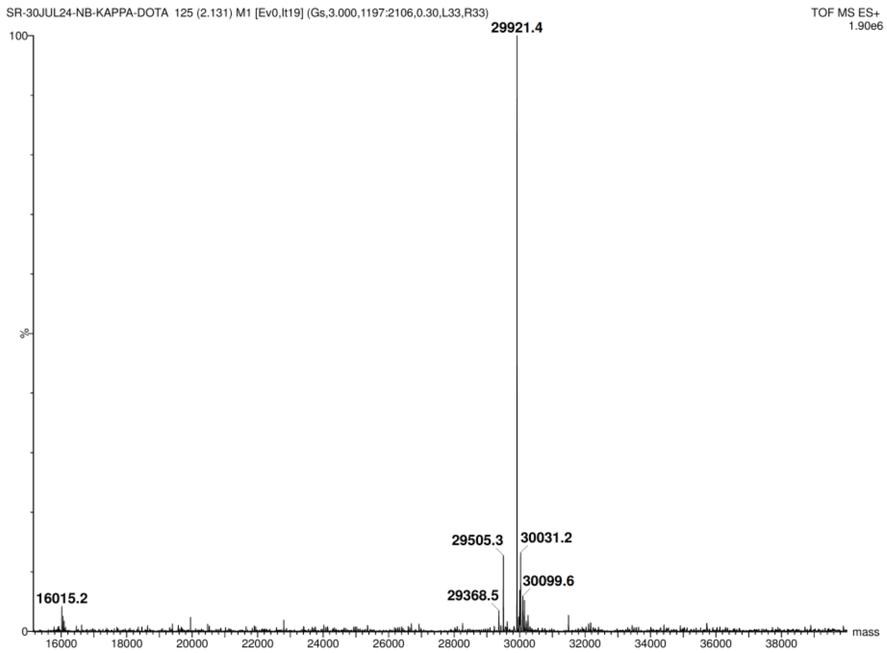

HN3-Nb$_{6E}$

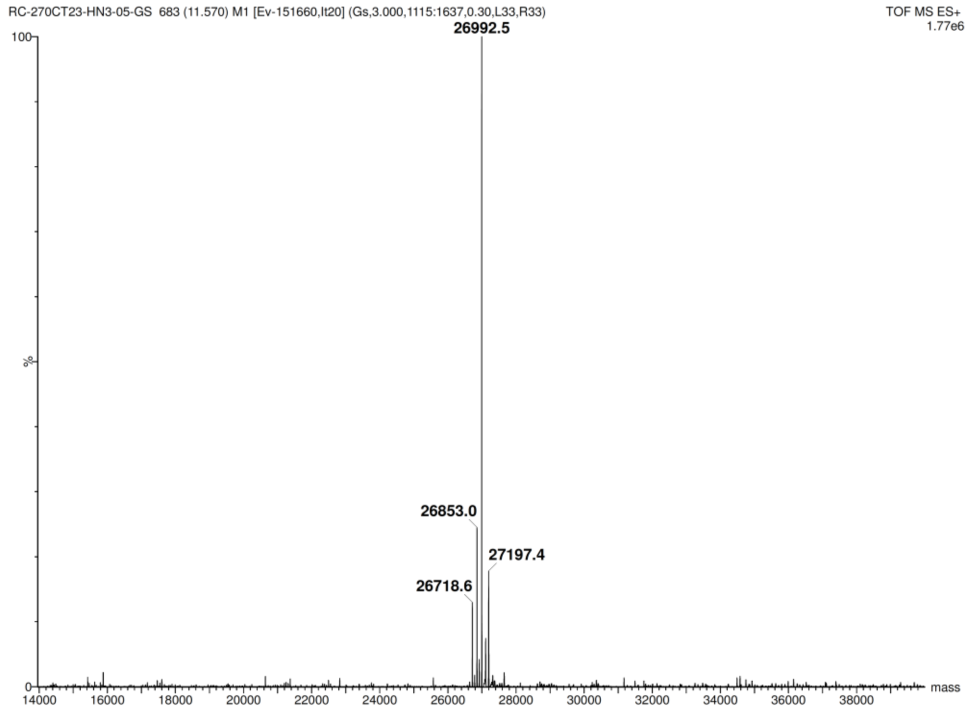

HN3-Nb$_{6E}$-DOTA-xlink in untreated 1x PBS

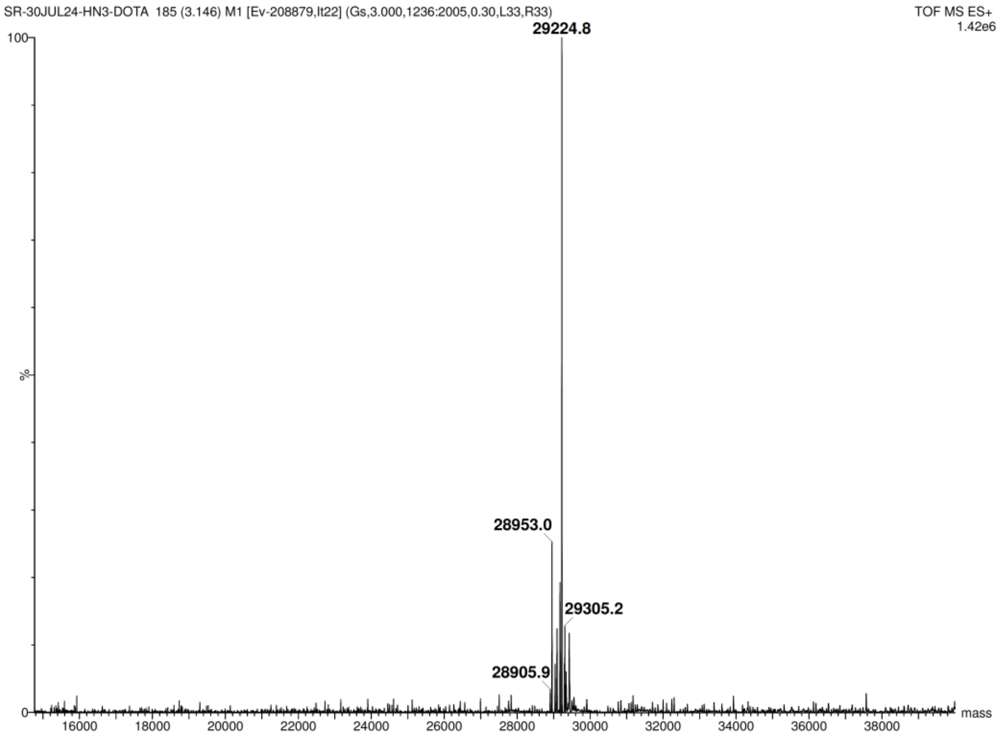

HN3-Nb$_{6E}$-DOTA-xlink with chelex treated 1xPBS buffer

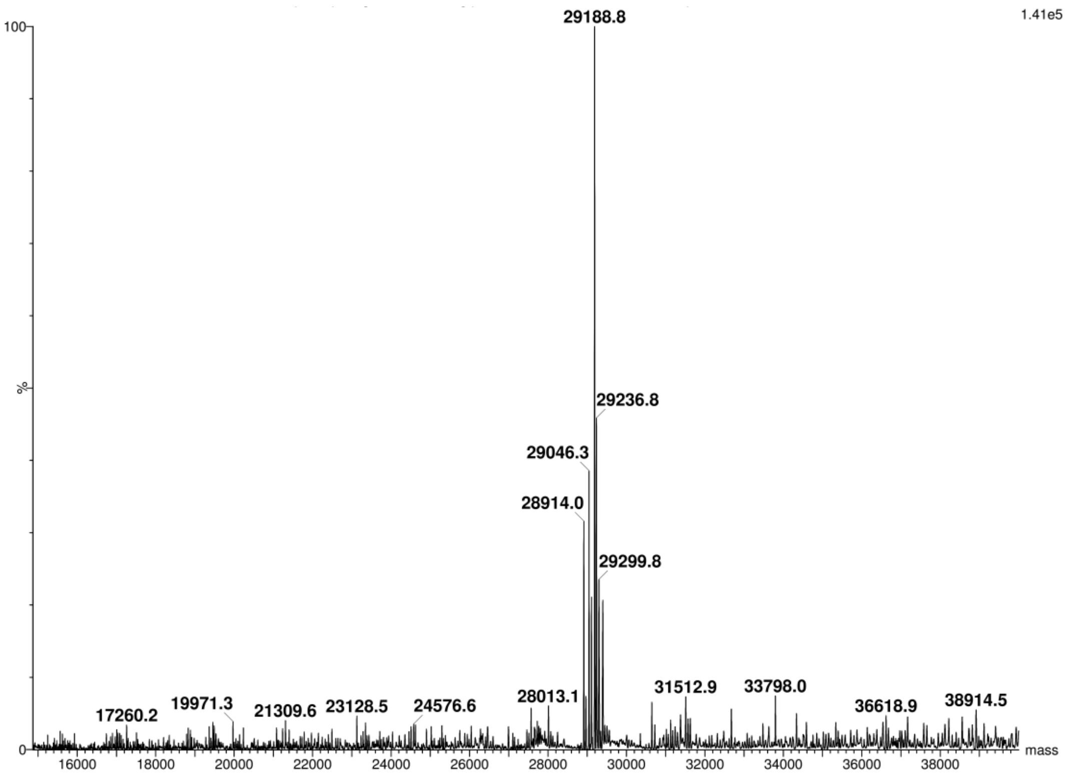

HN3-Nb_{6E}-LPET-G_3

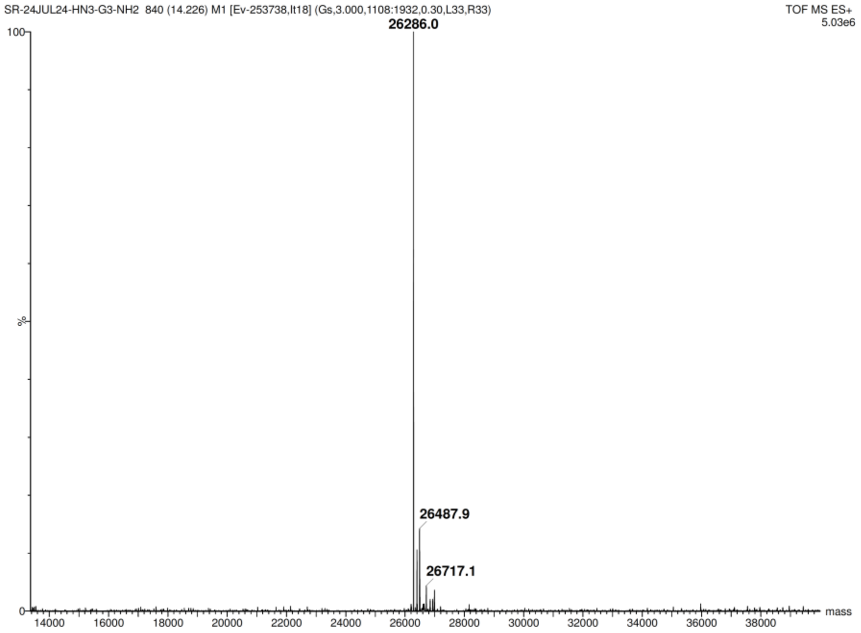

HN3-Nb_{6E}(DOTA)-LPET-G_3

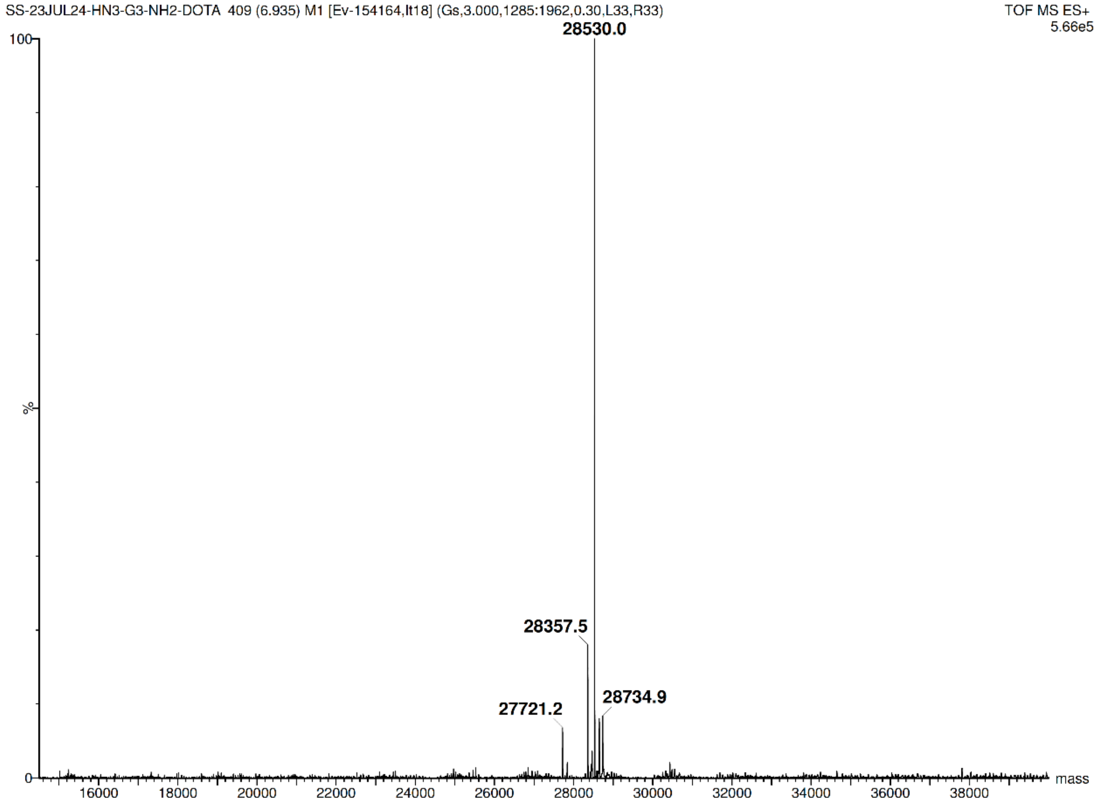

HN3-Nb6E + Azide-6E-xlink reaction (see Figure 3c)

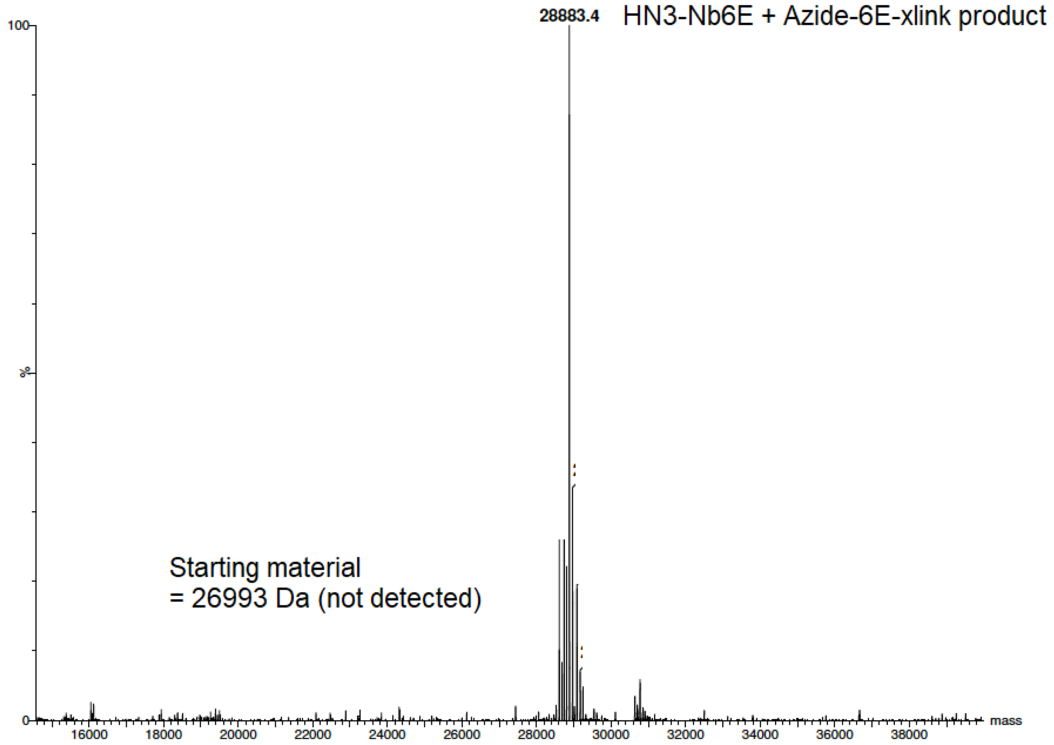

Uncropped Gel Images.

Uncropped Supporting Figure 2

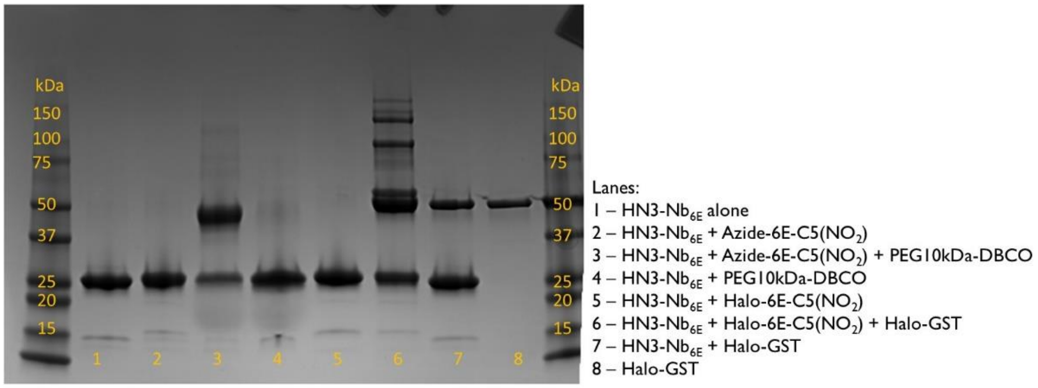

Uncropped gel image of Figure 2A and Supporting Figure 8

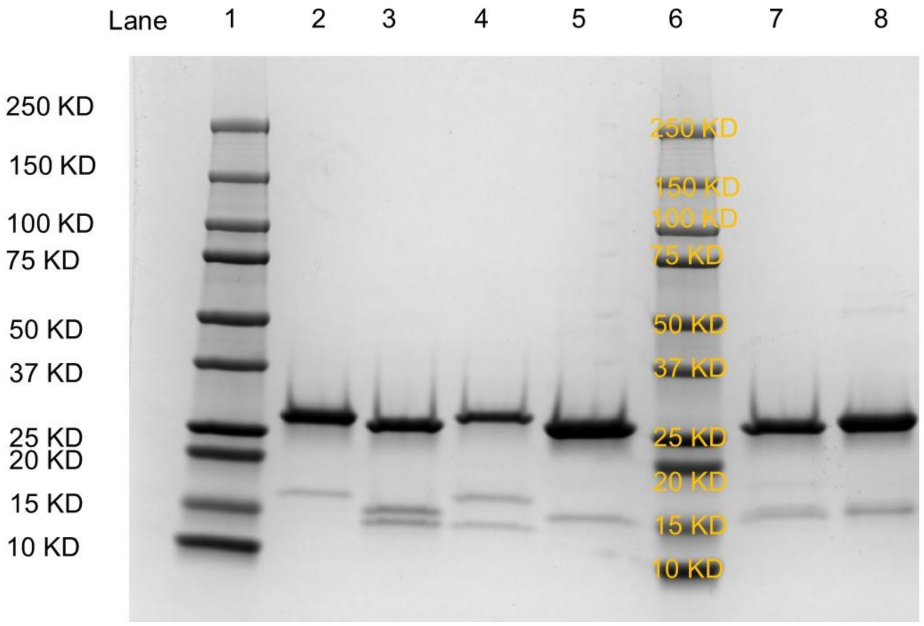

Lane #1- Marker
Lane #2- HN3-Nb_{6E}(DOTA)
Lane #3- Nb_{Kappa}-Nb_{6E}
Lane #4 Nb_{Kappa}-Nb_{6E}(DOTA)
Lane #5 HN3-Nb_{6E}
Lane #6 Marker
Lane #7 HN3-Nb_{6E}-G3
Lane #8 HN3-Nb_{6E}(DOTA)-G3

Uncropped Figure 3B

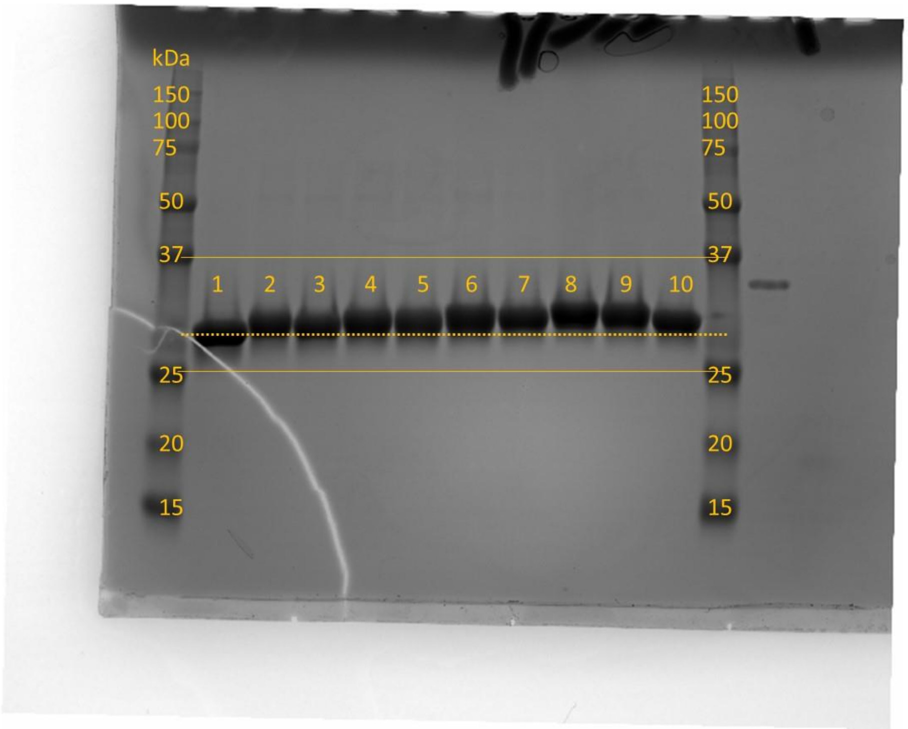

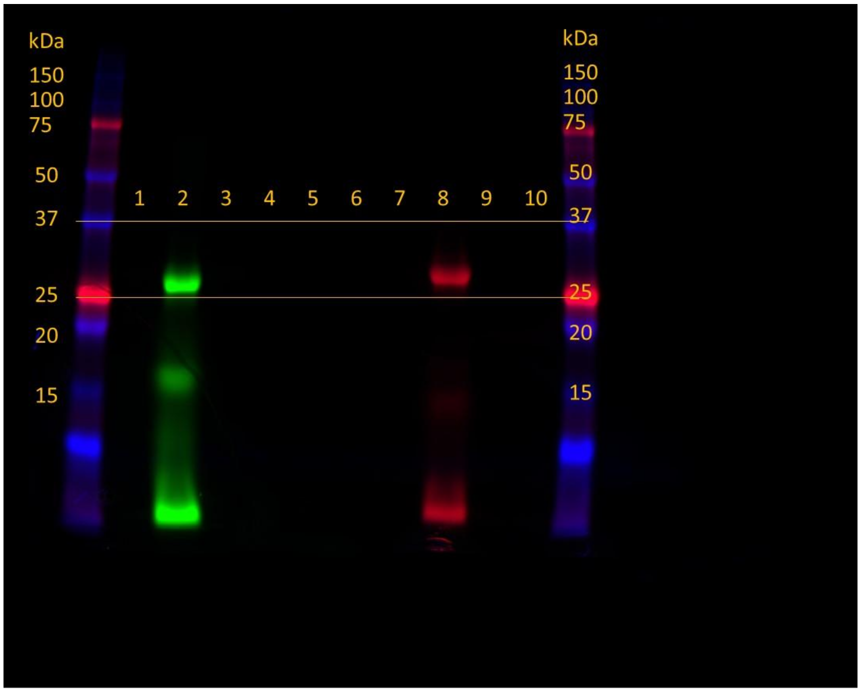

Uncropped Figure 3C

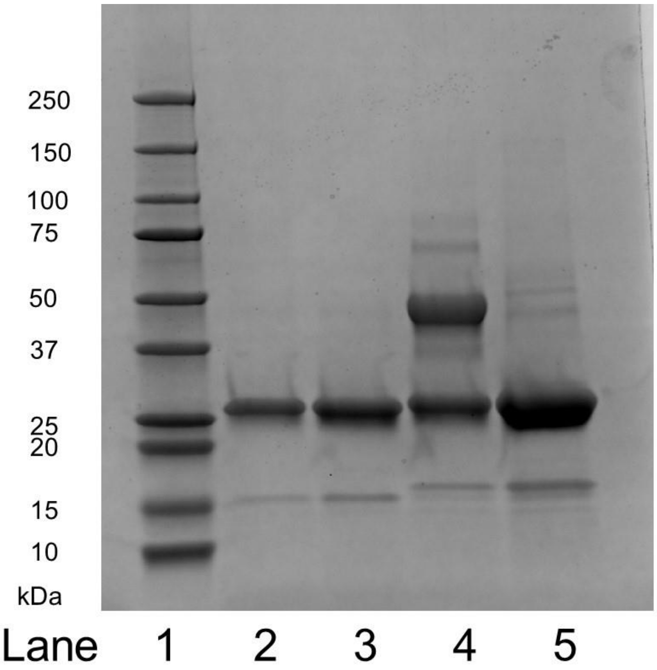

Lane#1 Ladder
Lane#2 HN3-Nb_{6E}
Lane#3 HN3-Nb_{6E}+PEG10kDa-DBCO
Lane#4 HN3-Nb6E+Azide-6E-xlink+PEG10kDa-DBCO
Lane#5 HN3-Nb_{6E}+Azide-xlink

Uncropped Supporting Figure 2

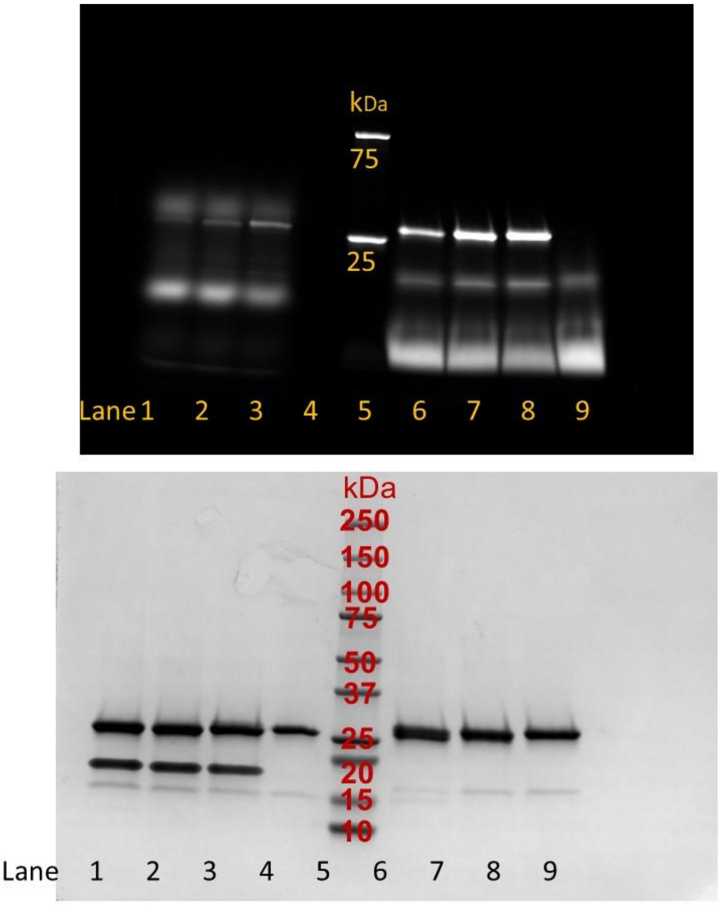

Lane#1 HN3-Nb6E+G3-FAM 10 min
Lane#2 HN3-Nb6E+G3-FAM 30 min
Lane#3 HN3-Nb6E+G3-FAM; 2 hrs
Lane#4 HN3-Nb6E
Lane#5 Ladder
Lane#6 HN3-Nb6E+FAM-6E- 10 min
Lane#7 HN3-Nb6E+FAM-6E-30 min
Lane#8 HN3-Nb6E+FAM-6E-2 hrs
Lane#9 FAM-6E-C5-xlink

Uncropped **Supporting Figure 4**

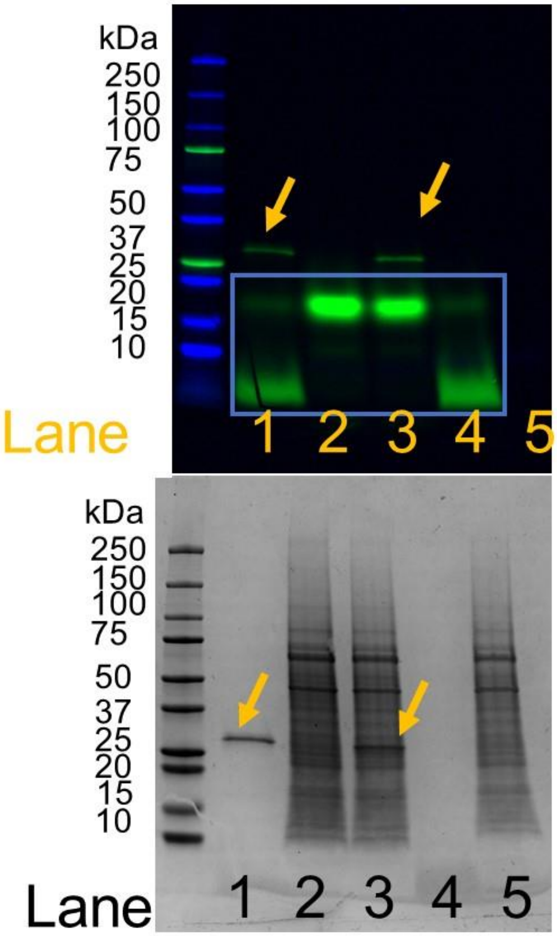

Lane:
1: HN3-Nb$_{6E}$ + FAM-6E-xlink
2: Lysate+ FAM-6E-xlink
3: HN3-Nb6E +Lysate + FAM-6E-xlink
4: FAM-6E-xlink only
5: Lysate only

Note that the fluorescence bands in the blue box correspond to signals from FAM-6E-xlink only (see lane 4).

Supporting References.

(1) Cabalteja, C. C.; Sachdev, S.; Cheloha, R. W. Rapid Covalent Labeling of Membrane Proteins on Living Cells Using a Nanobody–Epitope Tag Pair. *Bioconjug. Chem.* **2022**, *33* (10), 1867–1875. https://doi.org/10.1021/acs.bioconjchem.2c00334.
(2) Duan, Z.; Buffington, J.; Hong, J.; Ho, M. Production and Purification of Shark and Camel Single-Domain Antibodies from Bacterial and Mammalian Cell Expression Systems. *Curr. Protoc.* **2022**, *2* (6), e459. https://doi.org/10.1002/cpz1.459.
(3) Fayn, S.; King, A. P.; Gutsche, N. T.; Duan, Z.; Buffington, J.; Olkowski, C. P.; Fu, Y.; Hong, J.; Sail, D.; Baidoo, K. E.; Swenson, R. E.; Cheloha, R. W.; Ho, M.; Choyke, P.; Escorcia, F. Site-Specifically Conjugated Single-Domain Antibody Successfully Identifies Glypican-3–Expressing Liver Cancer by Immuno-PET. *J. Nucl. Med.* **2023**, jnumed.122.265171. https://doi.org/10.2967/jnumed.122.265171.
